# Synthesis and Regioselective Functionalization of Tetrafluorobenzo-[α]-Fused BOPYPY Dyes

**DOI:** 10.1021/acs.inorgchem.4c00499

**Published:** 2024-05-08

**Authors:** Sebastian O. Oloo, Guanyu Zhang, Petia Bobadova-Parvanova, Seleen Al Horani, Masa Al Horani, Frank R. Fronczek, Kevin M. Smith, Maria da Graça H. Vicente

**Affiliations:** † Department of Chemistry, Louisiana State University, Baton Rouge, Louisiana 70803, United States; ‡ Department of Chemistry and Fermentation Sciences, Appalachian State University, Boone, North Carolina 28608, United States

## Abstract

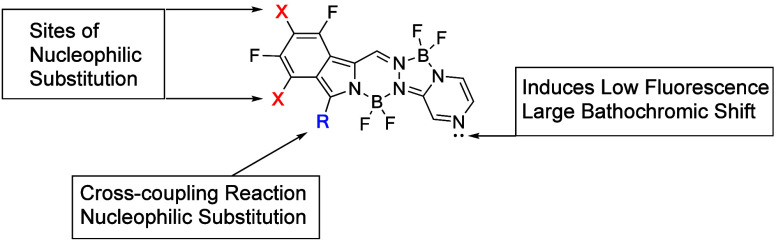

The synthesis of a new bis-BF_2_ tetrafluorobenzo-[α]-fused BOPYPY dye from 4,5,6,7-tetrafluoroisoindole and 2-hydrazinopyrazine is reported. The regioselectivity of nucleophilic substitution reactions at the periphery of the tetrafluorinated BOPYPY and its α-bromo derivative were investigated using N-, O-, S-, and C-based nucleophiles. Among the aromatic fluorine atoms, the F^2^ atom is consistently regioselectively substituted, except when the α-position contains a thiophenol group; in this case, F^4^ is substituted instead due to stabilizing π–π-stacking between the two aromatic groups. The α-bromo BOPYPY derivative also reacts under Stille cross-coupling reaction conditions to produce the corresponding α-substituted product. The spectroscopic properties of these new fluorinated BOPYPYs were investigated and compared with nonfluorinated analogs.

## Abstract 2

The synthesis and regioselective functionalization of unsymmetric tetrafluorobenzo-[α]-fused-BOPYPYs are reported. These fluorophores feature dual absorptions bands in the region 457−559 nm and relatively large Stokes shifts, in the order of 3000 cm^−1^. These BOPYPY dyes display low fluorescence quantum yields, due to internal conversion to an excited dark state, due to the lone pair of electrons on the pyrazine nitrogen atom.

## 1 Introduction

**Figure 1:**
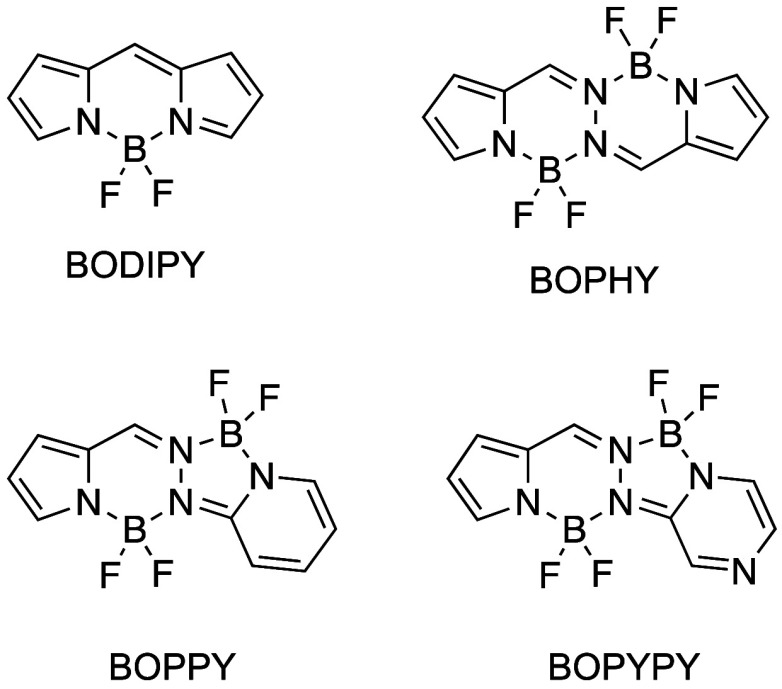
Core structures of BODIPY and bisBF_2_ derivatives BOPHY, BOPPY, and BOPYPY.

Heterocyclic chromophores have recently been a center of widespread investigations due to their extensive applications in biomedicine and material science.^1−6^ The diverse applications of these molecules demand unique optoelectronic properties and desirable chemical, biological and physical features. As a result, many useful organic fluorophores have been developed in pursuit of new molecules with desired properties.^3,7,8^ Among the most recently explored dyes, the 4,4-difluoro-4-bora-3a,4a-diaza-s-indacene or boron dipyrromethene dyes (abbreviated BODIPY, Figure 1) have attracted intense research interest owing to their favorable photostability, easy synthesis and structural modifications, and their tunable photophysical properties.^5,9^ In particular, BODIPY derivatives bearing two BF_2_-complexed groups were recently explored and observed to exhibit near unity fluorescence quantum yields and enhanced photostability as a result of their rigid planar structures.^9,10^ Among these, the bis(difluoroboron)1,2-bis((1H-pyrrol-2-yl)methylene)-hydrazine dyes (known as BOPHY, Figure 1) have found applications in multiple areas, including energy-transfer cascades, fluorescence imaging, solar cells, and cancer therapy.^11−14^ However, some BOPHYs show self-quenching effects and limited solubility as a result of their rigid symmetric cores, leading to reduced fluorescence quantum yields.^9^ To address this challenge, unsymmetric bisBF_2_ derivatives, known as BOPPY^15^ and BOPYPY^16^ (Figure 1), were recently synthesized and observed to display large Stokes shifts and high fluorescence quantum yields both in solution and in the solid state. These unsymmetric bisBF_2_ fluorophores are readily prepared in moderate to good yields from condensation of an α-formylpyrrole ring with 2-hydrazinylpyridine (in the case of BOPPY) or with 2-hydrazinylpyrazine (in the case of BOPYPY).^15,16^ Both BOPPY and BOPYPY dyes in general show large molar absorptivities, dual absorptions and emissions, and high photostability. In addition, these dyes are reported to exhibit strong solid-state emissions and two-photon absorptions in the near-IR region.^15^ Due to the presence of the additional N atom in the pyrazine ring, the absorption and emission of BOPYPY dyes are red-shifted by about 40–60 nm compared with those of the BOPPY analogs.^16^ To achieve bathochromic shifts in the absorption and emission bands, benzo-fused BOPPYs with extended π-systems have been recently reported;^17^ however, benzo-fused BOPYPY dyes have not yet been investigated.

Herein, we report the synthesis of new unsymmetric tetrafluorobenzo-[α]-fused-BOPYPYs **1** and **2**, and their regioselective functionalization by nucleophilic substitution reactions (S_N_Ar) at both the halogenated α-position and the aromatic fluorine sites. The electron-withdrawing fluorine atoms in these molecules further stabilize this type of chromophore relative to the currently known compounds.^18^ In addition to improving stability, advantages of introducing a tetrafluorobenzo-fused unit in the chromophore include (1) extending the chromophore π-system leading to longer wavelength absorptions and emissions, (2) allowing direct attachment of molecules via nucleophilic substitutions of one or more fluorine atoms, (3) allowing for^19^ F-radiolabeling for dual fluorescence and MRI imaging, and (4) improving solubility.^19,20^ In addition, a Stille coupling reaction was investigated on the α-bromo-BOPYPY derivative **2**, demonstrating the versatility of regioselective functionalization of this type of chromophore.

## 2 Results and Discussion

### 2.1 Synthesis and Structure Characterization

**Scheme 1:**
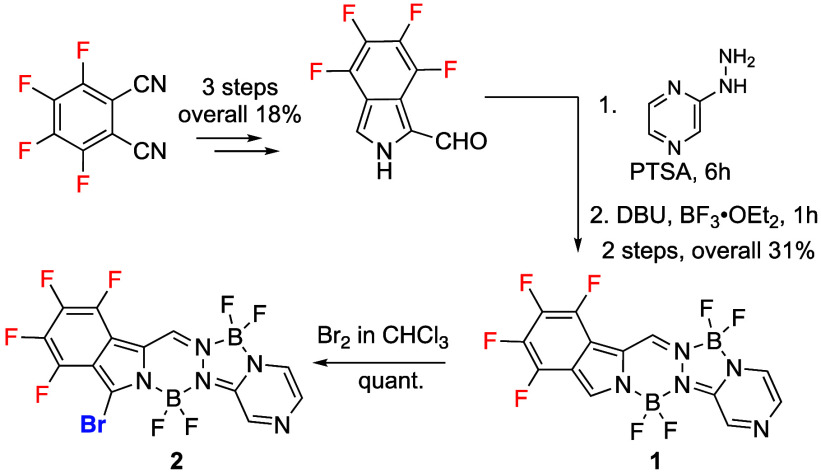
Synthesis of BOPYPY **1** and Its Brominated Derivative **2**

4,5,6,7-Tetrafluorobenzo[c]pyrrole-2-carbaldehyde is a useful starting material for the synthesis of fluorinated benzoporphyrins,^18^ perfluoro-benzo[α]-fused BODIPYs,^20^ and BOPHYs.^19^ Since this isoindole 2-carbaldehyde can readily be prepared from commercially available tetrafluorobenzonitrile in 3 steps with an overall yield of 18%, we decided to use it as starting material for the preparation of tetrabenzo-[α]-fused BOPYPY **1** (Scheme 1). We hypothesized that the benzo-fused moiety, as well as the pyrazine unit, would induce large bathochromic shifts on the absorption and emission wavelengths compared with currently known BOPPY and BOPYPYs. Furthermore, it would allow the investigation of aromatic fluorine substitution regioselectivity that is useful for direct conjugation with a variety of molecules to the chromophore for diverse applications.^19,20^

As shown in Scheme 1, the reaction of the tetrafluoroisoindole-2-carbaldehyde with 2-hydrazinopyrazine in the presence of *p*-toluenesulfonic acid (PTSA), followed by complexation with boron trifluoride etherate (BF_3_•OEt_2_) in 1,8-diazabicyclo[5.4.0]undec-7-ene (DBU), yielded BOPYPY **1** in 31% yield (Scheme 1). Regioselective bromination of BOPYPY **1** using bromine in chloroform^21^ occurred at the most electron-rich α-pyrrolic position, affording **2** in quantitative yield.

**Figure 2:**
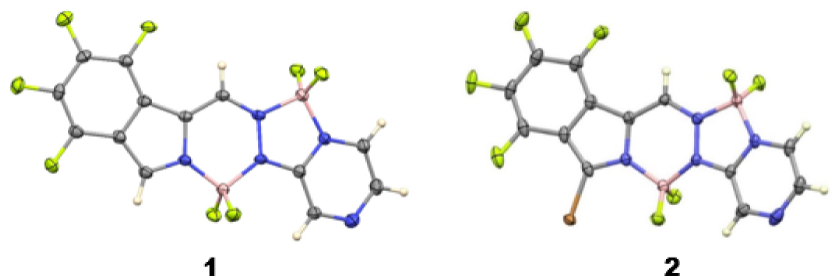
X-ray crystal structures of BOPYPY **1** (left) and its brominated derivative **2** (right) with 50% ellipsoids.

The structures of BOPYPYs **1** and **2** were characterized by^19^F, ^11^B, and ^1^H NMR spectroscopy (see Figures S1–S6) and HRMS (Figures S68–S69). The ^19^F NMR of BOPYPY **1** showed the four aromatic fluorines at −144.31, −147.83, −152.76, and −160.06 ppm, and the two distinct BF_2_ groups appear at −141.53 and −144.40 ppm. In the ^11^B NMR spectrum, two sets of triplets at 1.02 and 3.02 ppm were observed, corresponding to the two BF_2_ groups on the 6-membered and the 5-membered rings, respectively, based on previous studies.^19^ The ^1^H NMR of BOPYPY **1** showed five aromatic protons at 9.16, 8.34, 8.33, 8.26, and 7.86 ppm; the α-pyrrolic proton at 8.34 ppm disappeared upon bromination, to afford BOPYPY **2**. Crystals of BOPYPY **1** and its α-bromo derivative **2** suitable for X-ray analysis were obtained by slow diffusion of hexane into dichloromethane (Figure 2). The structures of **1** and **2** (as the substitutionally disordered crystal with the Br atom 67% occupied) have distinct differences and clearly show the asymmetry in the BOPYPY chromophore. In **1**, the molecule has a slightly bowed shape, with the central 6-membered C_2_BN_3_ ring being planar to within an average deviation of 0.02 Å, the F_4_Ph group forming a dihedral angle of 2.9° with it, and the pyrazine ring forming a 9.2° dihedral angle with it. The two boron atoms differ in their coordination geometry, with the one in the 6-membered ring having slightly shorter B–N distances (av. 1.556 Å) and slightly longer B–F distances (av. 1.378 Å) than those in the 5-membered ring (1.573 and 1.363 Å, respectively). On the other hand, the bowed shape in **2** is more pronounced than in **1**. The central 6-membered ring is nonplanar, with the boron atom lying 0.38 Å out of the plane of the other five atoms, the F_4_Ph group forming a dihedral angle of 9.2° with it, and the pyrazine ring forming a 12.4° dihedral angle with it. Furthermore, in the brominated derivative **2**, all B–N distances are similar, in the range 1.563(4)–1.568(4) Å, and all B–F distances are equal, in the range 1.363(4)–1.374(4) Å. The C–Br distance is 1.822(3) Å, slightly shorter than C(3)-Br distances on BODIPYs. TD-DFT calculations on the structures of **1** and **2** confirm that the two boron atoms in each compound differ in their geometry, with the 6-membered ring having slightly shorter B–N bond lengths compared to the 5-membered ring. In addition, since the two BF_2_ units are on different size rings, our calculations show that the N–B–N angles are around 10° smaller in the 5-membered ring compared with those in the larger 6-membered ring while the F–B–F angles differ only by 1–2°, in agreement with the crystal structures measurements.

### 2.2 Nucleophilic Substitution Reactions

**Scheme 2:**
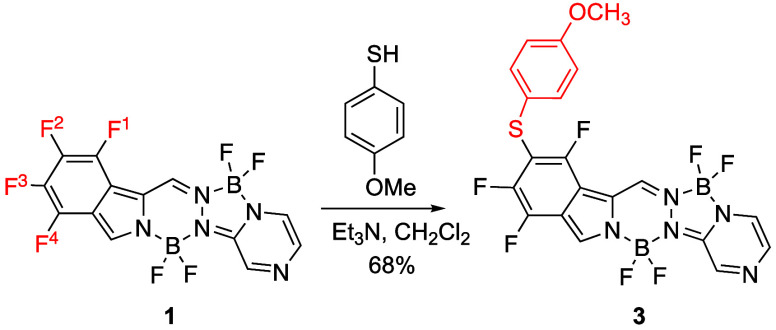
Regioselective S_N_Ar Reactions of BOPYPY **1** Using 4-Methoxythiophenol

Nucleophilic substitutions of halogenated BODIPY derivatives and other chromophores are a convenient methodology for the introduction of various types of nucleophiles, including S-, N-, O-, and C-centered examples, onto a chromophore to modulate its chemical and photophysical properties.^22,23^ In particular, the introduction of aromatic fluorines provides a useful strategy for direct conjugation of molecules via S_N_Ar reactions, under mild conditions. These reactions normally occur via a concerted two-step addition–elimination mechanism by nucleophilic attack on the electron-deficient benzo ring. We have previously reported that a symmetric bis(tetrafluorobenzo-[α]-fused) BOPHY derivative reacts in the presence of S-centered nucleophiles giving the corresponding substituted products with high regioselectivity.^19^ Therefore, we investigated the reactivity of unsymmetric BOPYPY **1** in the presence of the reactive 4-methoxythiophenol as the nucleophile (Scheme 2). The reaction occurred smoothly at room temperature overnight with high regioselectivity, giving BOPYPY **3** as the sole product in 68% yield. Minor byproducts containing two nucleophiles were also produced, albeit in low yield, as detected by mass spectrometry. The structure of **3** was confirmed by ^19^F, ^11^B, ^1^H NMR, and 2D ^1^H–^19^F HOESY spectroscopy (see Figures S7–S9,S49) and HRMS (Figure S70). In the ^19^F NMR of **3,** the three aromatic fluorines appear at −114.66 (F^1^), −135.30 (F^3^), and −145.84 ppm (F^4^) and the two BF_2_ groups on the 6- and 5-membered rings at −140.37 and −143.44 ppm, respectively. In the 2D ^1^H–^19^F HOESY, cross-peaks between F^1^ and the meso-H, and between F^4^ and the α-H were observed. The chemical shifts of the α-pyrrolic and meso-protons of BOPYPY **3** did not change significantly upon introduction of the *p*-methoxythiophenol substituent. In the ^11^B NMR spectrum, the boron atoms appear as triplets at 1.04 and 2.98 ppm.

We have previously observed similar regioselectivity in the S_N_Ar reactions of a tetrafluorobenzo-[α]-fused BOPHY in the presence of thiols, which occurred exclusively at the F^2^ position.^19^ To further understand the experimentally observed regioselectivity, we used DFT calculations to evaluate the charges on the tetrafluoro aromatic carbon atoms using two different schemes: NPA (natural population analysis) and MK (Merz–Singh–Kollman). However, neither of these schemes produced results that were able to explain the experimentally observed regioselectivity. As shown previously,^24,25^ the molecular electrostatic potentials (MESPs) at atomic nuclei might be better reactivity descriptors, since these are calculated directly from the electron densities without any additional approximations. These calculations showed that for BOPYPY **1** (see Figure S63), the least negative MESPs are located on the carbons attached to F^1^ and F^2^, suggesting that these are the most susceptible to nucleophilic attack, with the F^2^ carbon being slightly more reactive. In agreement with this result, our calculations indicate that the F^2^-substituted BOPYPY **3** and its F^1^-substituted analog have similar energies, with BOPYPY **3** being slightly more stable by approximately 0.5 kcal/mol.

**Scheme 3:**
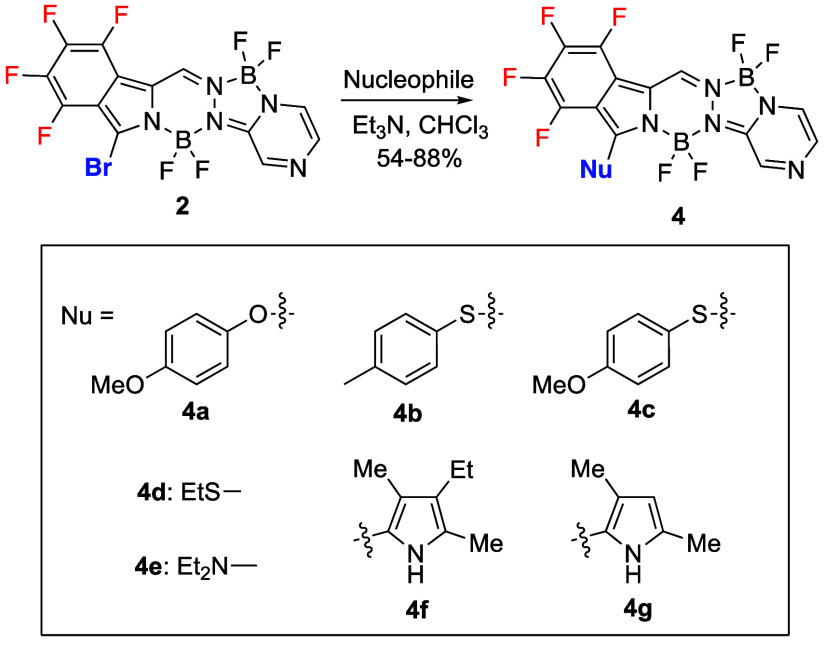
Regioselective S_N_Ar Reactions of BOPYPY **2** with S, O, N, and C Nucleophiles

Since increasing the concentration of nucleophile and the reaction temperature resulted in mixtures of products in low yields, we next investigated the regioselectivity of S_N_Ar reactions on the α-bromo BOPYPY **2**, in the presence of S-, N-, O-, and C-centered nucleophiles (Scheme 3).^26^ As expected, these reactions occurred with high selectivity at the brominated carbon atom bearing the best leaving group.

The corresponding mono-α-substituted BOPYPYs **4** were isolated after purification by silica gel column chromatography followed by recrystallization. The reaction conditions and the yields obtained in these reactions are summarized in Table 1 and reflect the reactivity of the nucleophile. The most reactive S- and N-centered nucleophiles readily reacted at room temperature in chloroform, while the less reactive O- and C-centered nucleophiles required heating in toluene, affording the corresponding products in moderate to high yields. 4-Methoxythiophenol reacted the fastest with BOPYPY **2** giving BOPYPY **4b** in 78% isolated yield, after 1 h at room temperature in chloroform solution. 4-Methylthiophenol and diethylsulfide gave similar high yields of **4c** and **4d** upon heating at 50 °C for 1 or 3 h, respectively. On the other hand, diethylamine reacted smoothly with BOPYPY **2** at room temperature overnight, affording BOPYPY **4e** in 88% yield, while 4-methoxyphenol required higher temperature (80 °C), giving BOPYPY **4a** in 54% isolated yield. The carbon nucleophiles 3-ethyl-2,4-dimethylpyrrole and 2,4-dimethylpyrrole also reacted with BOPYPY **2** in toluene at 80 °C for 3–5 h to afford the corresponding BOPYPY derivatives **4f** and **4g** in 63 and 79% yields, respectively.

**Table 1:** S_N_Ar Reaction Conditions and Yields for α-Substituted BOPYPYs **4**

BOPYPY	temp (°C)	time (h)	Nu equiv	solvent	yield (%)
**4a**	80	3	3.0	toluene	54
**4b**	r.t.	1	1.1	chloroform	78
**4c**	50	1	1.5	chloroform	77
**4d**	50	3	1.5	chloroform	83
**4e**	r.t.	16	2.0	chloroform	88
**4f**	80	3	4.0	toluene	63
**4g**	80	5	4.0	toluene	79

**Figure 3:**
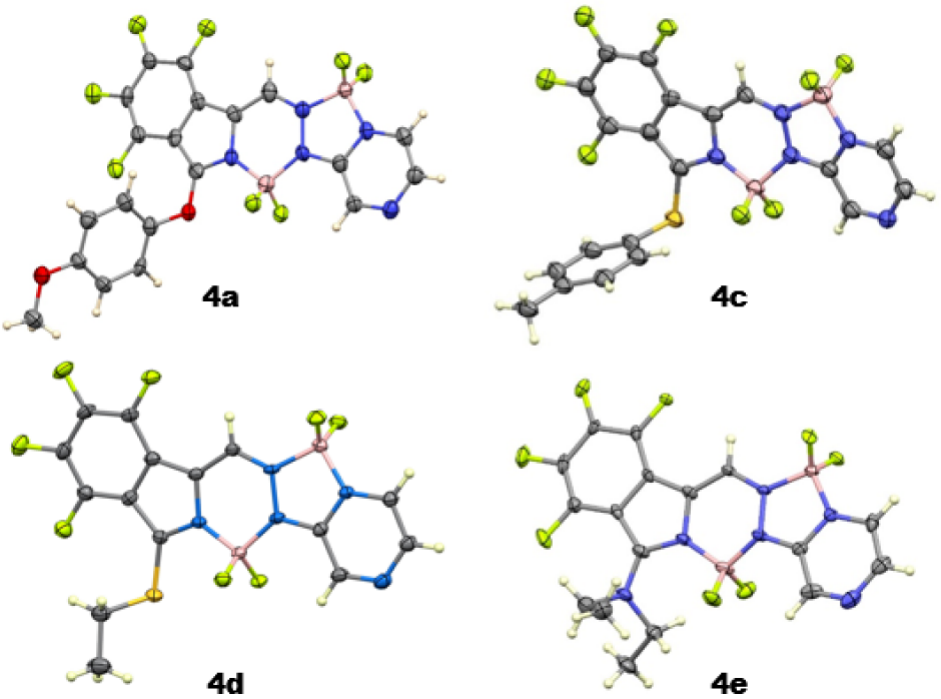
X-ray crystal structures of BOPYPYs **4a, 4c, 4d**, and **4e** with 50% ellipsoids.

All the monosubstituted derivatives **4** were stable under light, humidity, and air, and their structures were characterized by ^19^F, ^11^B, and ^1^H NMR spectroscopy (see Figures S10–S30) and HRMS (Figures S71–S77). In all the ^19^F NMR spectra of BOPYPYs **4**, the four distinct aromatic fluorines appeared consistently between −138.03 and −160.79 ppm, and the two BF_2_ groups at around −136.10 and −145.09 ppm, with the exception of **4f** and **4g**; in these cases, due to the hydrogen bond interaction between the pyrrolic NH and the neighboring BF_2_, the two fluorines on the 6-membered ring BF_2_ group show distinct chemical shifts in ^19^F NMR spectra. In the ^11^B NMR spectra, the two distinct triplets appeared at around 0.80 and 3.06 ppm for all the compounds. Slight shifts were observed in the ^1^H NMR chemical shifts of the meso-protons. Furthermore, the X-ray crystal structures for BOPYPYs **4a**, **4c, 4d**, and **4e** were obtained and are shown in Figure 3. Interestingly, the **4a** molecule has a bowed shape that differs from those of **1** and **2**. Its central C_2_BN_3_ ring is planar to within an average deviation of 0.01 Å, the F_4_Ph group forms a dihedral angle of 10.7° with it, and the pyrazine ring is nearly coplanar with it, forming a dihedral angle of only 1.1°. The methoxy substituent is distinctly twisted with respect to the core of the molecule, with a N–C–O–C torsion angle 125.2(7)°. Furthermore, the B–N and B–F distances in **4a** do not show the asymmetry seen in **1**, with mean values of 1.550 and 1.370 Å, respectively. The shape of the BOPYPY ring system of the **4c** molecule is like that of **1**, except that the central C_2_BN_3_ ring in this case is nonplanar, with the B atom 0.29 Å out of the plane of the other five atoms. The dihedral angles between the central ring and F_4_Ph, and between the central ring and the pyrazine are 0.9° and 9.7°, respectively. The N–C–S–C torsion angle to the ethylthio substituent is 114.9(6)°, and the B–N and B–F distances are not asymmetric, with average values of 1.574 and 1.364 Å, respectively. The C–S distance from the ring system to the S atom is 1.752(6) Å. The crystal structure of **4e** is unusual in that there are 13 molecules in the asymmetric unit. The largest differences among them are the different conformation of the ethyl groups of the diethylamine substituent. In a typical molecule, the central C_2_BN_3_ ring is nearly planar, with average deviation of 0.05 Å. The 5-ring system is closer to planarity than the previously described molecules, with the F_4_Ph group forming a dihedral angle of 3.7° with the central ring and the pyrazine forming a dihedral angle of 5.4°. Although the precision is not high for this structure, there does not appear to be any difference between the B–N and B–F distances for the two boron atoms.

**Scheme 4:**
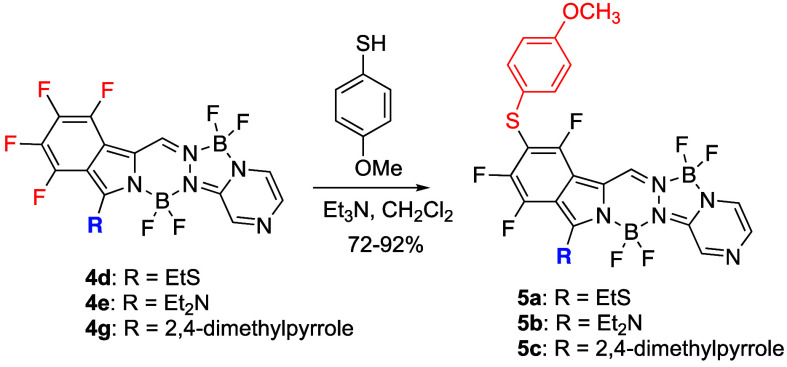
Regioselective S_N_Ar Reactions on Select BOPYPYs **4**

**Figure 4:**
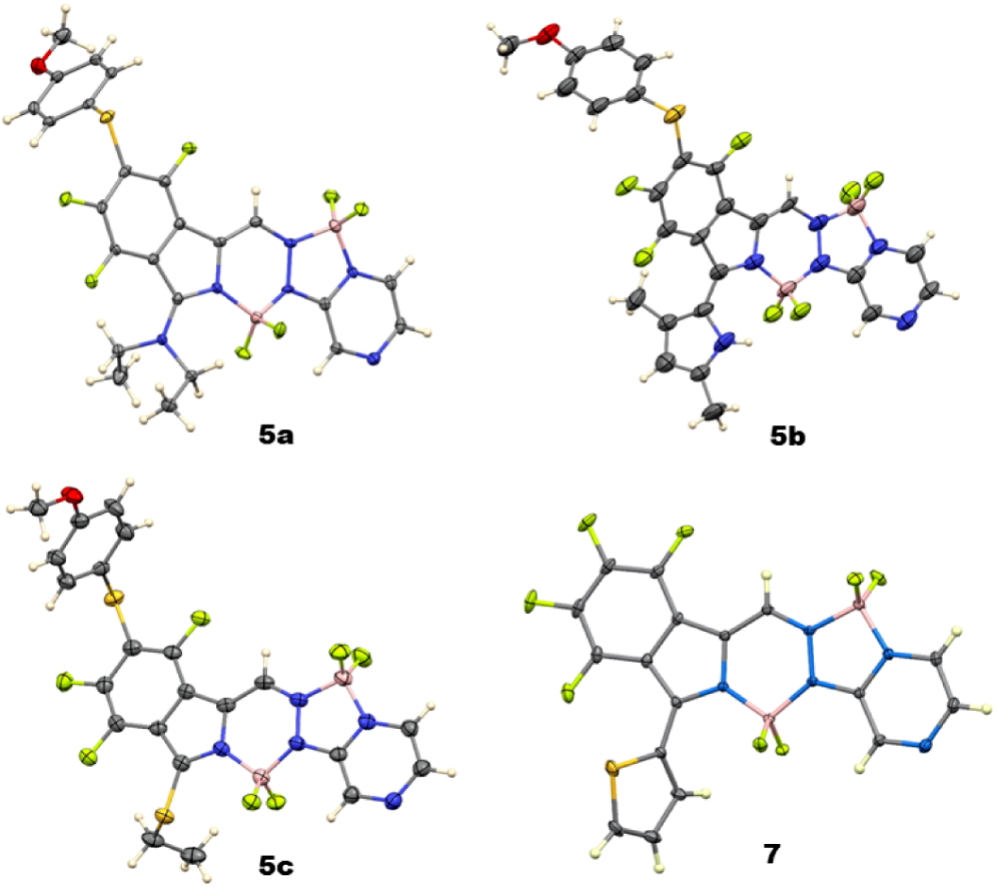
X-ray crystal structures of disubstituted BOPYPYs **5a**, **5b, 5c**, and **7** with 50% ellipsoids.

The regioselectivity of fluoride substitution on select BOPYPYs **4d, 4e**, and **4g** was then investigated in the presence of the most reactive 4-methoxythiophenol, as shown in Scheme 4. When BOPYPYs **4d, 4e**, and **4g** were reacted with a slight excess of the nucleophile at room temperature in dichloromethane solution, the corresponding BOPYPYs **5a, 5b**, and **5c** were isolated in 72, 92, and 84% yields, respectively. These BOPYPYs could also be prepared from **3** by first performing α-bromination followed by nucleophilic substitution using the appropriate nucleophile, albeit in lower overall yield. The structures of BOPYPYs **5a, 5b**, and **5c** were investigated using ^19^F, ^11^B, ^1^H NMR spectroscopy and 2D ^1^H–^19^F HOESY (see Figures S31–S39, S50 and S51), HRMS (Figures S78–S80), and by X-ray crystallography, as shown in Figure 4. As expected, as previously observed with a tetrafluorobenzo-[α]-fused BOPHY,^19^ the nucleophilic substitution occurs regioselectivity at the F^2^ position. These results are supported by the calculated MESP reactivity descriptors, which indicate that the F^2^ site is the most reactive toward nucleophilic substitution, except in the case of **4e**. Indeed, the performed calculations for compounds **4b**, **4d**, **4e**, and **4g** (see Figure S64) indicate that the α-substituent on BOPYPY **4** influences the values of the carbon MESPs. Interestingly, the effect is more pronounced on the bis-BF_2_ and pyrazine moieties; however, the tetrafluoro aromatic carbon MESPs also change upon α-substitution. In the case of **4e**, the F^4^ site bears the least negative MESP, but the proximity of the diethylamine group and its involvement in a hydrogen bonding interaction with F^4^ (Figure S64) reduce the reactivity at this site, making the F^2^ position the most favored site for reaction, in agreement with the experimental findings. It is also worth noting that our calculations indicate that the N-substituents tend to increase the regioselectivity for F^2^ substitution compared to the S-substituents.

As shown in Figure 4, the central C_2_BN_3_ ring in **5a** is slightly nonplanar, with the boron atom lying 0.25 Å out of the plane of the other five atoms. The fluorinated phenyl group is almost coplanar with it, forming a dihedral angle of 1.4°. The pyrazine ring is tilted by 8.7° from the central ring. In **5b**, the BOPYPY ring system is similar to that in **4e**, not deviating much from coplanarity. The central 6-membered ring has a mean deviation 0.07 Å, the phenyl ring forms a dihedral angle of 1.7° with it, and the pyrazine forms a dihedral angle of 5.7° with it. The B–N and B–F distances do not differ between the two boron atoms, and the C–S distance to the ring system is 1.775(3) Å. The precision of the structure determination of **5c** is lower, but it shows that the BOPYPY ring system is bowed. The central C_2_BN_3_ ring has a dihedral angle of 8.7° with the phenyl ring and a dihedral angle of 12.2° with the pyrazine ring. The dihedral angle between the BOPYPY core and the dimethylpyrrole substituent is 45.6°, and the N–H group forms an intramolecular hydrogen bond with the neighboring BF_2_ fluorine atom, having a N···F distance of 2.904 Å.

**Scheme 5:**
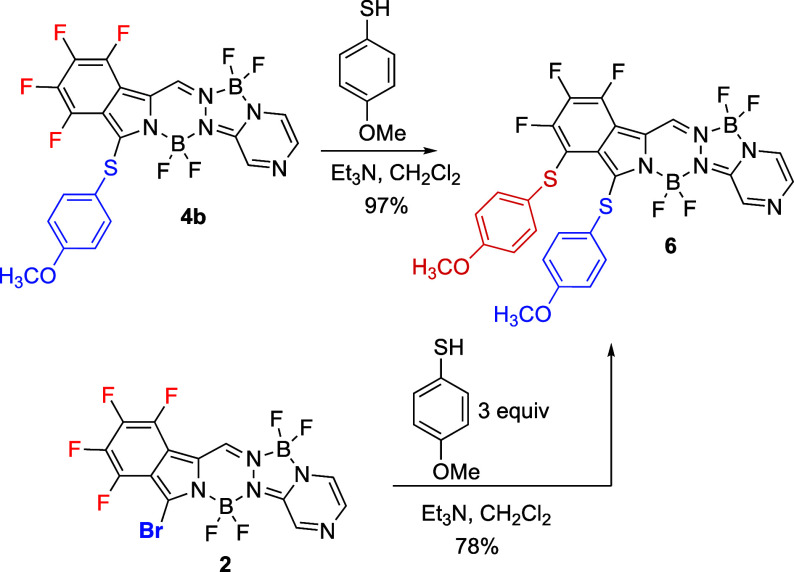
Regioselective S_N_Ar Reactions with Different Regioselectivity

Interestingly, different regioselectivity resulted when BOPYPY **4b** reacted with 4-methoxythiophenol at room temperature, as shown in Scheme 5. In this case, F^4^ was substituted instead, giving BOPYPY **6** as the only product, isolated in 97% yield. Similar results were also obtained when BOPYPY **2** was treated with an excess (3 equiv) of 4-methoxythiophenol; in this case, **6** was obtained in slightly lower yield (Scheme 5). A possible explanation for the observed regioselective substitution of F^4^ are the stabilizing π–π stacking interactions between the 4-methoxythiophenol molecules, which place the nucleophile in **4b** in very close proximity to F^4^ leading to its substitution, rather than at the more electronically favored F^2^. Indeed, we have observed similar regioselectively in the multisubstitution of a tetrafluorobenzo-[α]-fused BODIPY using an aromatic thiophenol as the nucleophile.^20^ Although the calculated MESPs for BOPYPY **4b** indicate that the F^2^ site is the most susceptible to nucleophilic substitution, compound **6** was found to be the most stable product among all possible regioisomers using the ωB97X-D DFT potential (Figures S64 and S65).^27^ Since the ωB97X-D method accounts for dispersion interactions, this result is in agreement with the hypothesized stabilizing π–π stacking interactions that lead to the formation of BOPYPY **6**.

The structure of BOPYPY **6** was further confirmed by ^19^F, ^11^B, ^1^H NMR and 2D ^1^H–^19^F HOESY spectroscopy (see Figures S40–S42 and S53). In the ^19^F NMR spectrum of **6**, the three fluorine atoms produced distinct signals at −115.23 (F^1^), −145.01 (F^2^), and −134.57 (F^3^) ppm; a cross-peak was observed in 2D ^1^H–^19^F HOESY between the meso-hydrogen at 8.18 ppm and the F^1^ atom of BOPYPY **6**, showing that the 4-methoxythiophenol group regioselectively replaced F^4^.

### 2.3 Stille Coupling Reaction

**Scheme 6:**
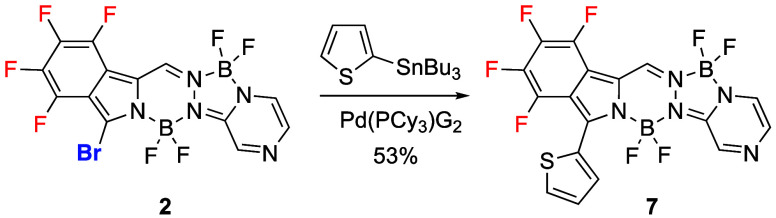
Regioselective Stille Cross-Coupling Reaction of BOPYPY **2**

The Stille cross-coupling reaction is an attractive methodology for the introduction of a variety of substituents under mild conditions onto halogenated BODIPYs and their derivatives.^26,28^ BOPYPY **2** reacted with an excess of 2-(tributylstannyl)thiophene in the presence of 5 mol % of chloro(tricyclohexylphosphine-2–2’aminobiphenyl) palladium [Pd(PCy_3_)G_2_] as the catalyst, giving BOPYPY **7** in 53% yield (Scheme 6). The structure of **7** was confirmed by^19^F, ^11^B, ^1^H NMR spectroscopy (see Figures S43–S45), HRMS (Figure S82), and X-ray crystallography, confirming the regioselectivity of the reaction. In BOPYPY **7 (**Figure 4), the 20-atom main ring system of is nearly planar, with mean deviation of only 0.034 Å. The thiophene plane makes a dihedral angle of 56.5° with it, and the B–N and B–F bond distances are like those of **4a**.

### 2.4 Spectroscopic Properties

**Figure 5:**
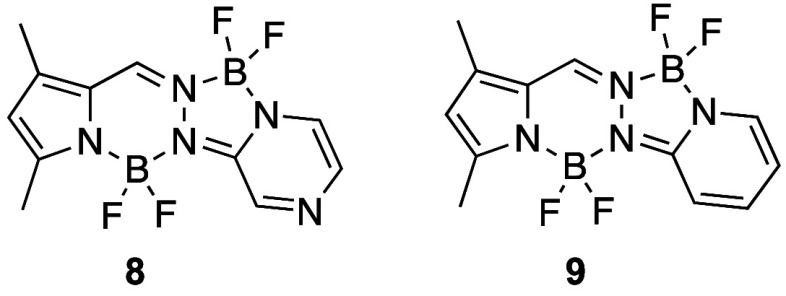
Chemical structures of previously reported BOPYPY **8**(16) and BOPPY **9**.^15^

The spectroscopic properties of the new fluorinated BOPYPYs were evaluated in dichloromethane and toluene solutions. The results obtained from these studies are summarized in Table 2, and the spectra are shown in the Figures S56–S62. For comparison purposes, the previously reported BOPYPY **8**^16^ and BOPPY **9**(15,17) (see Figure 5) were synthesized, and their spectroscopic properties evaluated under the same conditions.

**Table 2:** Spectroscopic Properties of BOPYPYs at Room Temperature, in Both CH_2_Cl_2_ and Toluene Solutions

	dichloromethane		toluene	
BOPYPY	λ_abs_^max^/nm (log ε)	λ_em_^max^ /nm (Φ_f_, %)^a^	λ_abs_^max^/nm (log ε)	λ_em_^max^ /nm (Φ_f_, %)^a^
**1**	457(4.25), 474(4.24)	523, 545(6.08)	459(4.22), 486(4.20)	522, 548(18.1)
**3**	462(4.61), 485(4.66)	553, 570(2.83)	475(4.50), 503(4.54)	540, 568(13.3)
**4a**	467(4.73), 484(4.72)	567, 614(0.84)	474(4.61), 489(4.59)	552, 584(3.75)
**4b**	472(4.52), 493(4.57)	576, 606(0.48)	481(4.51), 509(4.54)	562, 605(3.60)
**4c**	471s(4.53), 485(4.57)	556, 600s(2.54)	472s(4.48), 496(4.53)	554, 602(8.77)
**4d**	485(4.64), 471(4.60)	552, 612s(1.18)	470(4.56), 494(4.62)	559, 607(2.51)
**4e**	496(4.55), 518s(4.50)	-	500s(4.50), 526(4.40)	618, 673(0.23)
**4f**	537(4.30), 553(4.31)	562, 600s(1.24)	541(4.25), 566(4.28)	550, 598(5.08)
**4g**	518(4.16), 551(4.06)	546, 596s(2.38)	532(4.19), 563(4.23)	526, 565(6.71)
**5a**	488s(4.13), 517(4.21)	570, 613(0.53)	489s(4.11), 524(4.24)	567, 620(1.99)
**5b**	511(4.54), 537(4.48)	-	527(4.43), 553(4.35)	540, 628(0.31)
**5c**	538(4.27), 559(4.29)	565(0.24)	549(4.26), 574(4.28)	544, 583(3.56)
**6**	483s(4.54), 507(4.65)	589, 631s(0.46)	488s(4.44), 512(4.52)	570, 619(3.23)
**7**	478 (4.57)	560, 605s(1.70)	498(4.53), 519 (4.44)	563, 603(5.52)
**8**^b^	443(4.45), 468s(4.29)	528, 556(1.14)	440(4.50), 461s(4.41)	555, 520(2.74)
**9**^c^	410(4.62), 430(4.59)	461, 485(77.0)	413(4.60), 436(4.58)	460, 481(79.1)

a Fluorescence quantum yields (Φ_f_) determined using rhodamine 6G in methanol (Φ = 0.86) as standard.

b Reported fluorescence quantum yield in dichloromethane is Φ_f_ = 0.57.

c Reported fluorescence quantum yield in dichloromethane is Φf = 0.57.

**Figure 6:**
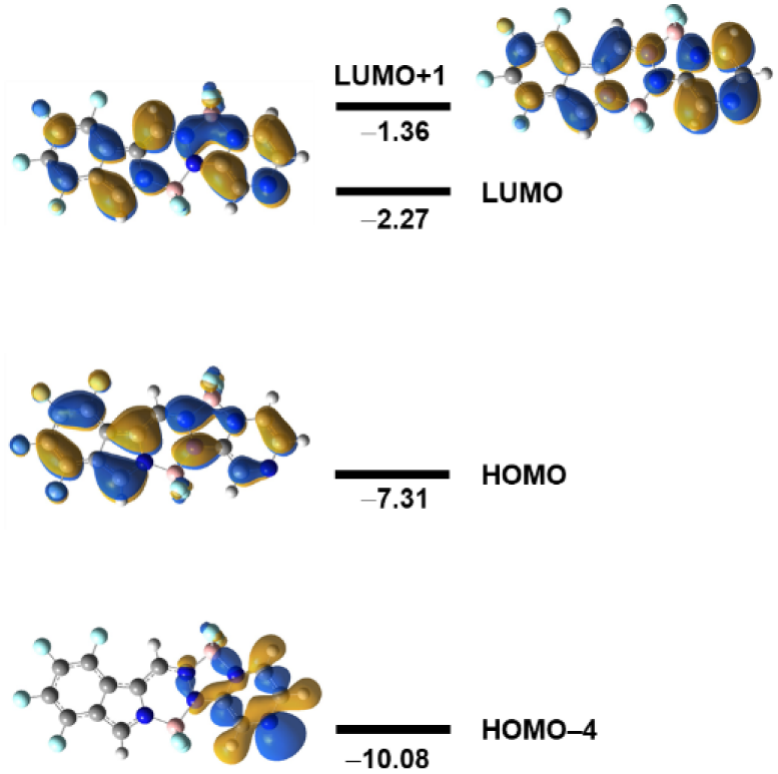
Frontier MO diagram for BOPYPY **1**. Energies in eV. Calculated at the TD-DFT M06-2*X*/6-31+G(d,p) level in dichloromethane.

As expected, BOPYPY **1** showed dual absorptions in both solvents with maximum absorption peaks at 457 and 474 nm in dichloromethane, which are 6–14 nm red-shifted from those of previously reported compound **8**. Larger red shifts of up to 110 nm were observed for the α-substituted BOPYPYs, as previously observed, due to the extension of the π-conjugation system of the chromophore. The larger red shift observed for BOPYPYs **4f** and **5c** is due to their smaller HOMO–LUMO gaps, as shown by DFT calculations (Table S1). The observed red shift is influenced by the smaller dihedral angle between the pyrrolic substituent and the BOPYPY core compared with **7**, as a result of hydrogen bonding between the pyrrolic NH and the adjacent BF_2_ group (see Figure 4). The split absorption bands of the BOPYPYs in both solvents are likely due to vibronic progressions of the same excited state, as previously suggested.^15^ Indeed, in agreement with these previous studies, our DFT calculations confirm a significant shortening of the N–N bond length upon excitation (see Table S1), varying from 0.032 to 0.058 Å. This is consistent with the more N–N antibonding character of the HOMO compared to the LUMO (see Figure 6). The strengthening of the N–N bond length in the current series of BOPYPYs is accompanied by weakening of the adjacent B–N bond lengths in both the 5-membered and the 6-membered rings and corresponding shortening of the neighboring B–N bond length. The vibronic progression explanation is consistent with the experimentally observed difference in the absorption and emission spectra in both dichloromethane and toluene. The increased interactions that occur in the more polar solvent result in smearing of the double peaks and the appearance of shoulders for several of the BOPYPYs, while the peaks are sharper and the split bands more easily observable in nonpolar toluene.

BOPYPY **1** shows a broad emission in dichloromethane with a maximum wavelength at 545 nm, red-shifted by about 23 nm relative to that previously reported for compound **8**.^16^ However, the fluorescence quantum yields of BOPYPY **8** in both solvents are very low, in contrast with the previously reported values.^16^ Indeed, the replacement of the pyridine ring of BOPPY with a pyrazine ring in BOPYPY results in large red shifts in the wavelengths of absorption and emission and also has a dramatic effect on the fluorescence quantum yields of the compounds. To further investigate these observations, we performed TD-DFT calculations of the ground and the excited states of the entire series of compounds and analyzed their molecular orbitals. For all studied compounds, the leading transition is between HOMO and LUMO. The experimentally observed red shifts in the absorption and emission wavelengths are due to stabilization of both HOMO and LUMO, with significantly greater effect on LUMO (Table S1). This results in a significantly smaller HOMO–LUMO gap in BOPYPY **8** compared to BOPPY **9** and therefore explains the observed red shift. One major difference between the electronic structure of the pyridine ring in BOPPYs and the pyrazine ring in BOPYPYs is the existence of the pyrazine N-lone pair MO that is absent in pyridine. This MO is present in the entire series of BOPYPYs studied. For example, Figure 6 shows the molecular orbital diagram in the case of BOPYPY **1**. The pyrazine N-lone pair molecular orbital appears as HOMO–4 and is localized mainly on the pyrazine. Just a small part of the 6-membered BF_2_ ring contributes to this MO, leaving the majority of the BOPYPY core with little to no electron density, resulting in a charge-transfer quenching of the fluorescence. Our TD-DFT calculations show that this charge-transfer transition occurs between HOMO–4 and LUMO and LUMO+1 and corresponds to excitation to S_3_. We hypothesize that the lower observed fluorescence in BOPYPYs compared to BOPPYs is the result of an internal conversion from S_1_ to the optically dark state (S_3_, in the case of BOPYPY **1**). This is in agreement with previously published detailed experimental and theoretical study of pyrazine,^29^ suggesting an internal conversion process involving a similar dark state. To further investigate this hypothesis, we performed TD-DFT calculations of the first 10 excited states for compounds **1**, and **3**–**9**. We found that similar charge-transfer transitions between the pyrazine N-lone pair molecular orbital and LUMO and LUMO+1 are observed for the entire series of BOPYPYs studied. As shown in Figure S67, the dark excited state appears as S_3_, S_4_, or S_5_, depending on the BOPYPY substituent(s). Such an excited state does not exist in the case BOPPY **9**. As expected, the energy of the dark excited state is relatively independent of the substituent on the isoindole moiety because the electron density is localized mainly on the pyrazine ring. For all BOPYPY compounds studied (**1**, **3**–**8**), we observed the existence of multiple excited states that are very close in energy, thus supporting the internal conversion hypothesis. The substituted BOPYPYs **3**, **4**, and **5** derivatives displayed even lower fluorescence quantum yields compared with BOPYPY **1**. Indeed, the substituents introduce additional excited levels that are very close in energy to the dark state, as shown in Figure S67. Our results are also in agreement with previous studies on 8-halo-BODIPYs and their 8-(C, N, O, S)-substituted analogs.^30^

Another characteristic of BOPYPY dyes is their large Stokes shifts, in the order of ca. 3000 cm^–1^. These values are larger than those reported for symmetric BODIPY systems and their derivatives, such as for aza-BODIPYs and BOPHYs.^31,32^ The performed TD-DFT calculations (Table S1) also demonstrate larger Stokes shifts for unsymmetric BOPYPYs compared with symmetric BOPHYs.^19^ The reason for the large Stokes shifts might be the significant change in the N–N and associated B–N bond lengths upon excitation. Except for **4f**, **4g**, and **5c**, the Stokes shifts in these compounds were similar to those observed for BOPYPY **1**.

In toluene, small red shifts were observed in the absorption spectra for most of the BOPYPYs synthesized, as previously observed for BODIPY derivatives. The relative fluorescence quantum yields of the BOPYPYs also increased in the nonpolar toluene relative to dichloromethane, as previously observed in the case of BOPPY dyes,^15,17^ due to the stronger interactions with the more polar solvent.

Although BOPYPYs display low fluorescence quantum yields, their strong absorptions (ε = 14300–53,700 M^–1^.cm^–1^) in the visible region of the optical spectrum warrant their investigation as potential optical sensors. Interestingly, we observed that upon addition of an excess of DBU (>50 equiv) to a dilute solution (ca. 10^–5^ M) of each BOPYPYs caused immediate loss of color. We hypothesize that the base attacks the BOPYPYs’ electron deficient meso-position leading to disruption of conjugation, similar to that observed in the case of BOPHYs.^19^ Studies on the potential application of these BOPYPYs as base sensors are currently underway in our laboratory.

## 3 Conclusions

We report the synthesis of a new series of fluorinated benzo-[α]-fused BOPYPY dyes with extended π-conjugated systems from those previously reported. These unsymmetric bisBF_2_ compounds display nearly planar or slightly bowed structures, with the two BF_2_ moieties having distinct coordination geometries in a 6- or 5-membered ring. The reactivity of α-bromo-BOPYPY **2** was investigated in nucleophilic substitutions with N-, O-, S-, and C-centered nucleophiles, and in a Pd(0)-catalyzed Stille cross-coupling reaction. The regioselectivity of nucleophilic substitution reactions at the aromatic fluorine atoms was investigated on the α-free BOPYPY **1** and on the α-substituted BOPYPYs **4b**, **4d**, **4e**, and **4g**. With the exception of **4b**, the F^2^ position is electronically and/or sterically favored, giving the corresponding product with high regioselectivity. In the case of BOPYPY **4b**, the F^4^ is the favored substitution site due to stabilizing π–π-interactions between the two aromatic groups.

The new BOPYPYs show dual absorptions due to vibronic progressions in the same excited state, as suggested by DFT calculations, and 6–55 nm red-shifted from those of previously reported compounds. They also show broad emissions and large Stokes shifts, in the order of 3000 cm^–1^, which are attributed to changes in the N–N and associated B–N bond lengths upon excitation. The relative fluorescence quantum yields of the BOPYPYs are lower than those observed for the related BOPPYs because of an internal conversion transition to a dark excited state due to the presence of the pyrazine N-lone pair molecular orbital. Our results show for the first time the drastic effect of presence of the N-lone pair on the pyrazine ring of BOPYPYs on the fluorescence properties of this type of dye. Nevertheless, BOPYPYs might find applications as optical sensors, for example for bases such as DBU.

## 4 Experimental Section

### 4.1 General

All reagents and solvents were purchased from commercial vendors and used as received without further purification, unless otherwise stated. Reactions were conducted in oven-dried glassware and monitored by plastic backed thin-layer chromatography plates. The plates were viewed by 254/365 nm UV indicator. Purification of the compounds was performed on silica backed preparative TLC plates from Sorbtech or by silica gel column chromatography (60 Å, 40–63 μm). All NMR spectra were recorded using a Bruker spectrometer operating at 400 MHz for ^1^H, 128 MHz for ^11^B, and 376 MHz for ^19^F. Chemical shifts (δ) are given in ppm relative to CDCl_3_ (7.26 ppm for ^1^H), acetone-d_6_ (2.05 ppm for ^1^H), CF_3_COOH in CDCl_3_ (−76.6 ppm for ^19^F), and BF_3_•OEt_2_ in CDCl_3_ (0.00 ppm for ^11^B). Coupling constants (*J*) are reported in Hz. Peak multiplicity is indicated as follows: s (singlet), d (doublet), t (triplet), q (quartet), dd (doublet of doublets), td (triplet of doublets), and m (multiplet). High-resolution mass spectra (HRMS) data were obtained at the LSU Mass Spectrometry Facility (MSF) using an Agilent 6230 ESI TOF, Waters Synapt XS and Bruker rapifleX MALDI TOF/TOF. 4,5,6,7-Tetrafluoroisoindole was prepared following a previously reported procedure, and the characterization data were in agreement with reported data.^18^ BOPYPYs **8**(16) and **9**(15) were also prepared according to the published procedure. While the spectroscopic data agree with that previously published for **9**, it is not in agreement with the data published for **8**. For BODIPY **8**: ^1^H NMR (400 MHz, CDCl_3_) δ9.06 (s, 1H), 8.12 (d, J = 3.8 Hz, 1H), 7.73–7.68 (m, 2H), 6.20 (s, 1H), 2.51 (s, 3H), 2.32 (s, 3H). HRMS (ESI-TOF) *m*/*z* [M + H]^+^ calcd for C_11_H_12_B_2_F_4_N_5_, 312.1215; found 312.1222. X-ray structures were obtained for both **8** and **9**, as shown in the Figures S53 and S54. Caution: the synthetic procedures use flammable organic solvents (acetone, chloroform, dichloromethane, toluene, ethyl acetate, and hexane) both as reaction solvents and for purification of the products.

### 4.2 Synthesis and Characterization

#### 4.2.1 BOPYPY **1**

*p*-Toluenesulfonic acid (4.76 mg, 0.028 mmol) was added to 4,5,6,7-tetrafluorobenzo[c]pyrrole-1-carbaldehyde (200 mg, 0.921 mmol) and 2-hydrazinopyrazine (111.6 mg, 1.01 mmol) in 1,2-dichloroethane (25 mL). The reaction mixture was left refluxing for 6 h. Upon complete disappearance of the starting material, 1.30 mL of DBU was added and the reaction left stirring for 10 min before slowly adding 1.20 mL of BF_3_•OEt_2_. The reaction mixture was further heated under reflux for 1 h, then cooled to room temperature before adding sat. NaHCO_3_ (50 mL) then extracting three times with CH_2_Cl_2_ (50 mL). The organic layers were combined, washed with water, dried over anhydrous Na_2_SO_4_, and the solvent was removed under reduced pressure. The crude product was purified by column chromatography using ethyl acetate/*n*-hexane (1:6) to afford **1** (114 mg, 31%) as a yellow solid. ^1^H NMR (400 MHz, CDCl_3_) δ 9.16 (s, 1H), 8.36–8.29 (m, 2H), 8.26 (s, 1H), 7.86 (dd, *J* = 3.8, 1.6 Hz, 1H). ^11^B NMR (128 MHz, CDCl_3_) δ 3.62–2.59 (m), 1.02 (t, *J* = 28.4 Hz). ^19^F NMR (376 MHz, CDCl_3_) δ −141.53 (dd, *J* = 56.1, 27.8 Hz, 2F), −144.31 (td, *J* = 18.7, 3.5 Hz, 1F), −144.40 (m, 2F) −147.83 (t, *J* = 19.3 Hz, 1F), −152.76 (td, *J* = 18.4, 3.5 Hz, 1F), −160.06 (t, *J* = 18.3 Hz, 1F). HRMS (ESI-TOF) *m*/*z* [M]^−^ calcd for C_13_H_5_B_2_F_8_N_5_, 405.0613; found 405.0649.

#### 4.2.2 BOPYPY **2**

Bromine liquid (157.9 μL, 0.988 mmol) was added dropwise to a stirring solution of BOPYPY **1** (100 mg, 0.247 mmol) in 50 mL of CHCl_3_ at 40 °C. After 12 h of stirring, the reaction was quenched with 50 mL of sat. Na_2_S_2_O_3_ solution, followed by extraction with CH_2_Cl_2_. The organic layers were combined, dried over anhydrous Na_2_SO_4_, and evaporated to dryness. The crude solid was recrystallized from hexane/dichloromethane to afford **2** (118 mg, 0.244 mmol) as an orange powder in quantitative yield. ^1^H NMR (400 MHz, CDCl_3_) δ 9.17 (s, 1H), 8.33 (d, *J* = 3.7 Hz, 1H), 8.17 (s, 1H), 7.86 (dd, *J* = 3.8, 1.6 Hz, 1H). ^11^B NMR (128 MHz, CDCl_3_) δ 3.03 (t, *J* = 23.7 Hz), 0.98 (t, *J* = 28.2 Hz). ^19^F NMR (376 MHz, CDCl_3_) δ −138.06 (dd, *J* = 56.3, 28.1 Hz, 2F), −144.12 to −144.48 (m, 2F), −147.72 (t, *J* = 19.1 Hz, 1F), −148.23 (td, *J* = 18.7, 4.1 Hz, 1F), −151.31 (td, *J* = 18.5, 4.1 Hz, 1F), −158.80 (t, *J* = 18.2 Hz, 1F). HRMS (ESI-TOF) *m*/*z* [M]^−^ calcd for C_13_H_4_B_2_BrF_8_N_5_, 482.9721; found 482.9725.

#### 4.2.3 BOPYPY **3**

One drop of NEt_3_ was added to a stirring solution of BOPYPY **1** (18 mg, 0.044 mmol) and 4-methoxythiophenol (12 μL, 0.098 mmol) in 3 mL of dichloromethane. The reaction mixture was left stirring at room temperature for 12 h after which the solvent was removed under reduced pressure, and the residue purified by column chromatography on silica gel using ethyl acetate/hexane 1/5 as eluent. Compound **3** (15.8 mg, 0.030 mmol) was collected as an orange powder in 68% yield. ^1^H NMR (400 MHz, CDCl_3_) δ 9.15 (s, 1H), 8.30 (d, *J* = 3.7 Hz, 1H), 8.28 (s, 1H), 8.25 (s, 1H), 7.84 (dd, *J* = 3.8, 1.6 Hz, 1H), 7.47 (d, *J* = 8.8 Hz, 2H), 6.84 (d, *J* = 8.8 Hz, 2H), 3.79 (s, 3H). ^11^B NMR (128 MHz, CDCl_3_) δ 2.98, 1.04 (t, *J* = 28.4 Hz). ^19^F NMR (376 MHz, CDCl_3_) δ −114.66 (d, *J* = 21.0 Hz, 1F), −135.30 (d, *J* = 20.3 Hz, 1F), −140.37 (dd, *J* = 56.3, 27.7 Hz, 2F), −143.44 (d, *J* = 31.2 Hz, 2F), −145.84 (t, *J* = 20.6 Hz, 1F). HRMS (ESI-TOF) *m*/*z* [M + H]^+^ calcd for C_20_H_13_B_2_F_7_N_5_OS, 526.0929; found 526.0829.

#### 4.2.4 BOPYPY **4a**

One drop of NEt_3_ was added to a stirring solution of BOPYPY **2** (15 mg, 0.031 mmol) and 4-methoxyphenol (11.6 mg, 0.093 mmol) in 3 mL of toluene. The solution was left stirring at 80 °C. for 3 h. The solvent was removed under reduced pressure, and the residue was purified by column chromatography on silica gel using ethyl acetate/hexane 1/6 as eluent. Compound **4a** (8.8 mg, 0.017 mmol) was obtained as an orange powder in 54% yield. ^1^H NMR (400 MHz, CDCl_3_) δ 9.08 (s, 1H), 8.22 (d, *J* = 3.8 Hz, 1H), 8.11 (s, 1H), 7.80 (dd, *J* = 4.0, 1.6 Hz, 1H), 7.07–7.01 (m, 2H), 6.92–6.85 (m, 2H), 3.81 (s, 3H). ^11^B NMR (128 MHz, CDCl_3_) δ 3.42–2.65 (m), 0.80 (t, *J* = 28.0 Hz). ^19^F NMR (376 MHz, CDCl_3_) δ −138.83 (td, *J* = 19.5, 5.0 Hz, 1F), −140.94 (dd, *J* = 55.0, 26.7 Hz, 2F), −143.68 to −143.96 (m, 2F), −147.36 (t, *J* = 19.2 Hz, 1F), −150.84 (td, *J* = 18.9, 5.0 Hz, 1F), −159.95 (t, *J* = 19.4 Hz, 1F). HRMS (ESI-TOF) *m*/*z* [M + H]^+^ calcd for C_20_H_12_B_2_F_8_N_5_O_2_, 528.1049; found 528.1050.

#### 4.2.5 BOPYPY **4b**

One drop of NEt_3_ was added to a stirring solution of BOPYPY **2** (15 mg, 0.031 mmol) and 4-methoxythiophenol (4.19 μL, 0.034 mmol) in 3 mL of chloroform. The solution was left stirring at room temperature for 1 h. The reaction solvent was removed under reduced pressure, and the residue was purified by column chromatography on silica gel using ethyl acetate/hexane 1/5 as eluent. Compound **4b** (13.2 mg, 0.024 mmol) was collected as an orange powder in 78% yield. ^1^H NMR (400 MHz, CDCl_3_) δ 9.22 (s, 1H), 8.31 (d, *J* = 3.8 Hz, 1H), 8.18 (s, 1H), 7.85 (dd, *J* = 3.8, 1.6 Hz, 1H), 7.48 (d, *J* = 8.8 Hz, 2H), 6.82 (d, *J* = 8.9 Hz, 2H), 3.78 (s, 3H). ^11^B NMR (128 MHz, CDCl_3_) δ 3.56–2.54 (m), 1.29 (t, *J* = 28.3 Hz). ^19^F NMR (376 MHz, CDCl_3_) δ −135.43 (dd, *J* = 56.8, 27.1 Hz, 2F), −143.09 (td, *J* = 19.2, 4.4 Hz, 1F), −144.13 to −144.52 (m, 2F), −148.17 (t, *J* = 19.5 Hz, 1F), −152.75 (td, *J* = 19.0, 4.3 Hz, 1F), −159.45 (t, *J* = 18.8 Hz, 1F). HRMS (ESI-TOF) *m*/*z* [M + H]^+^ calcd for C_20_H_12_B_2_F_8_N_5_OS, 544.0821; found 544.0815.

#### 4.2.6 BOPYPY **4c**

One drop of NEt_3_ was added to a stirring solution of BOPYPY **2** (10 mg, 0.021 mmol) and 4-methylbenzenethiol (3.85 mg, 0.031 mmol) in 3 mL of chloroform. The solution was left stirring at 50 °C for 1 h, and the reaction solvent was removed under reduced pressure; the residue was purified by column chromatography on silica gel using ethyl acetate/hexane 1/5 as eluent. Compound **4c** (8.4 mg, 0.016 mmol) was collected as an orange powder in 77% yield. ^1^H NMR (400 MHz, CDCl_3_) δ 9.21 (s, 1H), 8.31 (d, I = 3.8 Hz, 1H), 8.19 (s, 1H), 7.85 (dd, *J* = 3.9, 1.6 Hz, 1H), 7.34 (d, *J* = 8.0 Hz, 2H), 7.09 (d, *J* = 8.0 Hz, 2H), 2.30 (s, 3H). ^11^B NMR (128 MHz, CDCl_3_) δ 3.55–2.53 (m), 1.28 (t, *J* = 28.2 Hz). ^19^F NMR (376 MHz, CDCl_3_) δ −135.72 (dd, *J* = 56.6, 26.8 Hz, 2F), −143.47 (td, *J* = 19.2, 4.4 Hz, 1F), −144.20 to −144.52 (m, 2F), −148.16 (t, *J* = 19.5 Hz, 1F), −152.72 (td, *J* = 19.0, 4.3 Hz, 1F), −159.33 (t, *J* = 18.7 Hz, 1F). HRMS (ESI-TOF) *m*/*z* [M + H]^+^ calcd for C_20_H_12_B_2_F_8_N_5_S, 528.0872; found 528.0864.

#### 4.2.7 BOPYPY **4d**

One drop of NEt_3_ was added to a stirring solution of BOPYPY **2** (15 mg, 0.031 mmol) and ethanethiol (3.44 μL, 0.047 mmol) in 3 mL of chloroform. The solution was left stirring at 50 °C for 3 h. The solvent was removed under reduced pressure, and the residue purified by column chromatography on silica gel using ethyl acetate/hexane 1/6 as eluent. Compound **4d** (11.9 mg, 0.026 mmol) was collected as an orange powder in 83% yield. ^1^H NMR (400 MHz, CDCl_3_) δ 9.18 (s, 1H), 8.30 (d, *J* = 3.7 Hz, 1H), 8.16 (s, 1H), 7.84 (dd, *J* = 3.6, 1.8 Hz, 1H), 3.19 (q, *J* = 7.5 Hz, 2H), 1.32 (t, *J* = 7.4 Hz, 3H). ^11^B NMR (128 MHz, CDCl_3_) δ 3.59–2.48 (m), 1.15 (t, *J* = 28.5 Hz). ^19^F NMR (376 MHz, CDCl_3_) δ −136.10 (dd, *J* = 56.5, 27.4 Hz, 2F), −144.28 to −144.62 (m, 2F), −146.30 (td, *J* = 19.0, 4.3 Hz, 1F), −147.96 (t, *J* = 19.4 Hz, 1F), −152.89 (td, *J* = 18.9, 4.2 Hz, 1F), −159.70 (t, *J* = 18.5 Hz, 1F). HRMS (ESI-TOF) *m*/*z* [M + H]^+^ calcd for C_15_H_10_B_2_F_8_N_5_S, 466.0715; found 466.0717.

#### 4.2.8 BOPYPY **4e**

A solution of BOPYPY **2** (15 mg, 0.031 mmol) and HNEt_2_ (4.79 μL, 0.047 mmol) in 3 mL of chloroform were left stirring at room temperature for 16 h. The reaction solvent was removed under reduced pressure, and the residue was purified by column chromatography on silica gel using ethyl acetate/hexane 1/7 as eluent. Compound **4e** (13 mg, 0.027 mmol) was collected as a red powder in 88% yield. ^1^H NMR (400 MHz, CDCl_3_) δ 9.04 (s, 1H), 8.07 (d, *J* = 3.8 Hz, 1H), 7.89 (s, 1H), 7.72 (dd, *J* = 3.9, 1.6 Hz, 1H), 3.63 (q, *J* = 7.1 Hz, 4H), 1.21 (t, *J* = 7.1 Hz, 6H). ^11^B NMR (128 MHz, CDCl_3_) δ 3.00 (t, *J* = 24.9 Hz), 0.87 (t, *J* = 30.3 Hz). ^19^F NMR (376 MHz, CDCl_3_) δ −137.84 to −138.23 (m, 3F), −142.52 (dd, *J* = 48.5, 22.5 Hz, 2F), −146.73 (t, *J* = 19.2 Hz, 1F), −151.40 (td, *J* = 19.4, 5.6 Hz, 1F), −160.79 (t, *J* = 19.5 Hz, 1F). HRMS (ESI-TOF) *m*/*z* [M + H]^+^ calcd for C_17_H_15_B_2_F_8_N_6_, 477.1416; found 477.1424.

#### 4.2.9 BOPYPY **4f**

A solution of BOPYPY **2** (15 mg, 0.031 mmol) and 3-ethyl-2,4-dimethylpyrrole (16.5 μL, 0.124 mmol) in 3 mL of toluene were left stirring at 80 °C for 3 h. The reaction solvent was removed under reduced pressure and the residue was purified by column chromatography on silica gel using ethyl acetate/hexane 1/4 as eluent. Compound **4f** (10.3 mg, 0.020 mmol) was collected as a red powder in 63% yield. ^1^H NMR (400 MHz, CDCl_3_) δ 9.05 (s, 1H), 8.89 (s, 1H), 8.18 (d, *J* = 3.8 Hz, 1H), 8.11 (s, 1H), 7.78 (dd, *J* = 3.8, 1.5 Hz, 1H), 2.48 (q, *J* = 7.4 Hz, 2H), 2.35 (s, 3H), 2.07 (d, *J* = 4.3 Hz, 3H), 1.15 (t, *J* = 7.5 Hz, 3H). ^11^B NMR (128 MHz, CDCl_3_) δ 3.59–2.53 (m), 1.20 (t, *J* = 31.0 Hz). ^19^F NMR (376 MHz, CDCl_3_) δ −141.74 (td, *J* = 18.8, 4.7 Hz, 1F), −142.35 to −143.26 (m, 1F), −144.30 to −145.73 (m, 2F), −148.58 (t, *J* = 19.3 Hz, 1F), −152.89 (td, *J* = 19.3, 5.3 Hz, 1F), −160.69 (t, *J* = 18.7 Hz, 1F). HRMS (ESI-TOF) *m*/*z* [M + H]^+^ calcd for C_21_H_17_B_2_F_8_N_6_, 527.1575; found 527.1573.

#### 4.2.10 BOPYPY **4g**

A solution of BOPYPY **2** (15 mg, 0.031 mmol) and 2,4-dimethylpyrrole (12.8 μL, 0.124 mmol) in 3 mL of toluene were left stirring at 80 °C for 5 h. The reaction solvent was removed under reduced pressure, and the residue was purified by column chromatography on silica gel using ethyl acetate/hexane 1/4 as eluent. Compound **4g** (12.2 mg, 0.025 mmol) was collected as a brown powder in 79% yield. ^1^H NMR (400 MHz, CDCl_3_) δ 9.06 (s, 1H), 8.92 (s, 1H), 8.21 (d, *J* = 3.8 Hz, 1H), 8.14 (s, 1H), 7.80 (dd, *J* = 3.8, 1.6 Hz, 1H), 6.00 (d, *J* = 2.7 Hz, 1H), 2.39 (s, 3H), 2.12 (d, *J* = 3.7 Hz, 3H). ^11^B NMR (128 MHz, CDCl_3_) δ 3.02, 1.20 (t, *J* = 30.7 Hz. ^19^F NMR (376 MHz, CDCl_3_) δ −124.44 to −125.32 (m,1F), −141.51 (td, *J* = 18.5, 4.4 Hz, 1F), −141.69 to −142.26 (m, 1F), −143.18 to −144.59 (m, 2F), −147.55 (t, *J* = 19.2 Hz, 1F), −151.79 (td, *J* = 19.1, 5.1 Hz, 1F), −159.47 (t, *J* = 18.5 Hz, 1F). HRMS (ESI-TOF) *m*/*z* [M]^+^ calcd for C_19_H_12_B_2_F_8_N_6_, 498.1183; found 498.1191.

#### 4.2.11 BOPYPY **5a**

One drop of NEt_3_ was added to a stirring solution of BOPYPY **4d** (17 mg, 0.037 mmol) and 4-methoxythiophenol (5.40 μL, 0.044 mmol) in 4 mL of dichloromethane. The reaction mixture was left stirring at room temperature for 12 h before removing the solvent under reduced pressure, and the residue was then purified by column chromatography on silica gel using ethyl acetate/hexane 1/5 as eluent. Compound **5a** (12 mg, 0.021 mmol) was collected as an orange powder in 72% yield. ^1^H NMR (400 MHz, CDCl_3_) δ 9.18 (s, 1H), 8.28 (d, *J* = 3.8 Hz, 1H), 8.17 (s, 1H), 7.82 (dd, *J* = 3.8, 1.6 Hz, 1H), 7.48 (d, J = 8.8 Hz, 2H), 6.85 (d, *J* = 8.9 Hz, 2H), 3.80 (s, 3H), 3.18 (q, *J* = 7.4 Hz, 2H), 1.31 (t, *J* = 7.4 Hz, 3H). ^11^B NMR (128 MHz, CDCl_3_) δ 3.51–2.56 (m), 1.19 (t, *J* = 28.5 Hz). ^19^F NMR (376 MHz, CDCl_3_) δ −115.00 (d, *J* = 21.5 Hz, 1F), −134.65 to −135.13 (m, 3F), −143.27 to −143.66 (m, 2F), −147.79 (t, *J* = 20.6 Hz, 1F). HRMS (ESI-TOF) *m*/*z* [M + H]^+^ calcd for C_22_H_17_B_2_F_7_N_5_OS_2_, 586.0949; found 586.0950.

#### 4.2.12 BOPYPY **5b**

One drop of NEt_3_ was added to a stirring solution of BOPYPY **4e** (13 mg, 0.027 mmol) and 4-methoxythiophenol (7.05 μL, 0.057 mmol) in 3 mL of dichloromethane. The reaction mixture was left stirring at room temperature for 12 h before removing the solvent under reduced pressure, and the residue was then purified by column chromatography on silica gel using ethyl acetate/hexane 1/6 as eluent. Compound **5b** (14.9 mg, 0.025 mmol) was collected as a red powder in 92% yield. ^1^H NMR (400 MHz, CDCl_3_) δ 9.03 (s, 1H), 8.06 (d, *J* = 3.8 Hz, 1H), 7.89 (s, 1H), 7.70 (dd, *J* = 3.9, 1.6 Hz, 1H), 7.48 (d, *J* = 8.9 Hz, 2H), 6.85 (d, *J* = 8.9 Hz, 2H), 3.80 (s, 3H), 3.61 (qd, *J* = 7.1, 1.3 Hz, 4H), 1.19 (t, *J* = 7.1 Hz, 6H). ^11^B NMR (128 MHz, CDCl_3_) δ 3.56–2.51 (m), 0.89 (t, *J* = 30.4 Hz). ^19^F NMR (376 MHz, CDCl_3_) δ −116.03 (d, *J* = 20.8 Hz, 1F), −137.21 (d, *J* = 21.7 Hz, 1F), −138.62 to −139.01 (m, 2F), −141.39 (t, *J* = 21.3 Hz, 1F), −143.51 (dd, *J* = 46.4, 19.9 Hz, 2F). HRMS (ESI-TOF) *m*/*z* [M + H]^+^ calcd for C_24_H_22_B_2_F_7_N_6_OS, 597.1650; found 597.1660.

#### 4.2.13 BOPYPY **5c**

One drop of NEt_3_ was added to a stirring solution of BOPYPY **4g** (12 mg, 0.024 mmol) and 4-methoxythiophenol (8.00 μL, 0.051 mmol) in 3 mL of dichloromethane. The reaction mixture was left stirring at room temperature for 12 h before removing the solvent under reduced pressure, and the residue was then purified by column chromatography on silica gel using ethyl acetate/hexane 1/5 as eluent. Compound **5c** (12.5 mg, 0.020 mmol) was collected as a red powder in 84% yield. ^1^H NMR (400 MHz, CDCl_3_) 9.05 (s, 1H), 8.89 (s, 1H), 8.18 (d, *J* = 3.8 Hz, 1H), 8.14 (s, 1H), 7.78 (dd, *J* = 3.8, 1.6 Hz, 1H), 7.50 (d, *J* = 8.8 Hz, 2H), 6.86 (d, *J* = 8.9 Hz, 2H), 5.99 (d, *J* = 2.7 Hz, 1H), 3.80 (s, 3H), 2.38 (s, 3H), 2.11 (d, *J* = 3.6 Hz, 3H). ^11^B NMR (128 MHz, CDCl_3_) δ 3.59–2.63 (m), 1.22 (t, *J* = 31.1 Hz). ^19^F NMR (376 MHz, CDCl_3_) δ −115.65 (d, *J* = 20.7 Hz, 1F), −124.18 to −125.10 (m, 1F), −135.37 (d, *J* = 20.0 Hz, 1F), −141.52 to −142.28 (m, 1F), −143.52 to −144.44 (m, 2F), −144.19 (td, *J* = 20.4, 3.7 Hz, 1F). MALDI TOF/TOF *m*/*z* [M]^+^ calcd for C_26_H_19_B_2_F_7_N_6_OS, 618.1419; found 618.1415.

#### 4.2.14 BOPYPY **6**

One drop of NEt_3_ was added to a stirring solution of BOPYPY **2** (15 mg, 0.031 mmol) and 4-methoxythiophenol (11 μL, 0.093 mmol) in 3 mL of dichloromethane. The solution was left stirring at room temperature for 1 h. The solvent was removed under reduced pressure, and the residue was purified by column chromatography on silica gel using ethyl acetate/hexane 1/4 as eluent. Compound **6** (16 mg, 0.024 mmol) was collected as an orange powder in 78% yield. ^1^H NMR (400 MHz, CDCl_3_) δ 9.21 (s, 1H), 8.29 (d, *J* = 3.8 Hz, 1H), 8.18 (s, 1H), 7.83 (dd, *J* = 3.8, 1.6 Hz, 1H), 7.49 (d, *J* = 8.8 Hz, 2H), 7.44 (d, *J* = 8.8 Hz, 2H), 6.84–6.80 (m, 4H), 3.78 (s, 3H), 3.77 (s, 3H). ^11^B NMR (128 MHz, CDCl_3_) δ 3.61–2.34 (m), 1.31 (t, J = 28.6 Hz). ^19^F NMR (376 MHz, CDCl_3_) δ −115.23 (d, *J* = 21.4, 1.6 Hz, 1F), −134.17 (dd, *J* = 56.2, 26.3 Hz, 2F), −134.57 (d, *J* = 20.2, 1.5 Hz, 1F), −143.26 to −143.61 (m, 2F), −145.01 (t, *J* = 20.8 Hz, 1F). HRMS (ESI-TOF) *m*/*z* [M + H]^+^ calcd for C_27_H_19_B_2_F_7_N_5_O_2_S_2_, 664.1049; found 664.1018.

#### 4.2.15 BOPYPY **7**

Compound **2** (15 mg, 0.031 mmol), tributyl(thiophen-2-yl)stannane (19.7 μL, 0.062 mmol), and 3% mol Pd(PCy3)G2 (0.886 mg, 0.002 mmol) were combined in a flask. The flask was evacuated and refilled with nitrogen three times. Dry toluene (5 mL) was added and the reaction mixture stirred at 85 °C for 5 h under N_2_. Toluene was removed under reduced pressure, and the resulting crude product was purified by column chromatography using ethyl acetate/hexane 1/5 as eluent. BOPYPY **7** was collected as an orange powder in 53% yield (8.1 mg, 0.017 mmol). ^1^H NMR (400 MHz, *d*_6_-acetone) δ 8.98 (s, 1H), 8.66 (s, 1H), 8.47 (d, *J* = 3.8 Hz, 1H), 8.33 (dd, *J* = 3.8, 1.6 Hz, 1H), 7.90 (dd, *J* = 5.0, 1.2 Hz, 1H), 7.71–7.66 (m, 1H), 7.30 (dd, *J* = 5.1, 3.7 Hz, 1H). ^11^B NMR (128 MHz, *d*_6_-acetone) δ 3.08 (t, *J* = 23.4 Hz), 1.31 (t, *J* = 28.9 Hz). ^19^F NMR (376 MHz, *d*_6_-acetone) δ −134.30 (dd, *J* = 58.1, 28.9 Hz, 2F), −145.28 to −145.65 (m, 3F), −149.26 (t, *J* = 19.1 Hz, 1F), −157.31 (td, *J* = 18.2, 3.6 Hz, 1F), −163.36 (t, *J* = 18.1 Hz, 1F). HRMS (ESI-TOF) *m*/*z* [M + H]^+^ calcd for C_17_H_8_B_2_F_8_N_5_S, 488.0565; found 488.0572.

### 4.3 X-ray Analyses

Data for compounds **1**, **2**, **4a**, **4c**, **4e**, **5a**, and **5b** were collected on a Bruker Kappa ApexII DUO diffractometer, while those for **4d**, **5c**, **7**, **8**, and **9** were collected on a Bruker D8 Venture DUO Photon III diffractometer. Compound **5b** used MoKα radiation, **7**–**9** used AgKα, and all others used CuKα. All data were collected at 100 K except for **9** at 296 K because of a phase change at low temperature. Crystals of **4c** and **5a** were twinned. **4d** and **8** had two independent molecules, while **4e** had 13. Compound **5b** contained a small amount of a chlorinated impurity, while **2** and **7** contained small amounts of brominated impurities.

### 4.4 Spectroscopic Analyses

UV–vis absorption and emission spectra were collected at room temperature on a Varian Cary 50 spectrophotometer or on a PerkinElmer LS55 spectrophotometer, respectively. Dilute solutions (1.0 × 10^–6^ to 5.0 × 10^–6^ M) using spectrophotometric grade solvents in quartz cuvettes (1 cm path length) were used to minimize reabsorption effects. The relative fluorescence quantum yields (Φ_F_) were calculated using rhodamine 6G (Φ_F_ = 0.86 in MeOH) as reference using the following equation:^33^ Φ_X_ = Φ_ST_ × Grad_X_/Grad_ST_ × (η_X_/η_ST_)^2^, where the Φ_X_ and Φ_ST_ are the quantum yields of the sample and standard, Grad_X_ and Grad_ST_ are the gradients from the plot of integrated fluorescence intensity vs absorbance, and η represents the refractive index of the solvent.

### 4.5 Theoretical Calculations

All structures were optimized without symmetry constrains, and the stationary points were confirmed with frequency calculations. Solvent effects were taken into account using the polarized continuum model (PCM).^34^ The reported energies, atomic charges, and molecular electrostatic potentials were evaluated at the ωB97X-D/6-311++G(d,p) level to include empirical dispersion.^27^ The atomic charges were calculated using the NPA^35^ and MK schemes.^36^ The UV–vis absorption data were calculated using the TD-DFT method^37^ at the M06-2*X*/6-31+G(d,p) level^38^ as recommended in the literature.^39,40^ All calculations were performed using the Gaussian 09 program package.^41^

## Supporting Information Available

1D and 2D NMR data (PDF) for all BOPYPYs, absorption and emission spectra (PDF), and X-ray data (CIF). The Supporting Information is available free of charge. CIFs for **1**, **2**, **4a**, **4c**, **4e**, **5b**, and **5c** have been deposited at the Cambridge Crystallographic Data Center with deposition numbers CCDC 2309699–2309705. CIFs for **5a**, **4d**, and **7** have been deposited as CCDC 2324980–2324982. CIFs for **8** and **9** have been deposited as CCDC 2325327–2325328 (PDF)

## Abbreviations

PTSA: *p*-toluenesulfonic acid
DBU: 1,8 diazabicyclo[5.4.0]undec-7-ene
TD-DFT: time-dependent density-functional theory
HOESY: heteronuclear overhauser effect spectroscopy
NMR: nuclear magnetic resonance
TLC: thin layer chromatography
HRMS: high resolution mass spectrometry
MESPs: molecular electrostatic potentials
NPA: natural population analysis
HOMO: highest occupied molecular orbital
LUMO: lowest unoccupied molecular orbital
