# Supplementary material for: Synthesis and Regioselective Functionalization of Tetrafluorobenzo-[α]-Fused BOPYPY Dyes
Source: Inorg Chem. 2024 May 8;63(20):9164–74. doi: 10.1021/acs.inorgchem.4c00499 (PMC11110013; doi:10.1021/acs.inorgchem.4c00499)
Supplement: Supplementary file 1 — ic4c00499_si_001.pdf [file ic4c00499_si_001.pdf]

Supporting information

Synthesis and Regioselective Functionalization of Tetrafluorobenzo-[α]-fused BOPYPY Dyes

Sebastian Oloo, Guanyu Zhang, Petia Bobadova-Parvanova, Seleen Al Horani, Masa Al Horani, Frank R. Fronczek, Kevin M. Smith and Maria da Graça H. Vicente*

[1]Department of Chemistry, Louisiana State University, Baton Rouge, LA 70803

(*vicente@lsu.edu)

[2]Department of Chemistry and Fermentation Sciences, Appalachian State University, Boone, NC 28608

Table of Contents

1. 1D NMR SPECTRA.. 2

2. 2D NMR SPECTRA.. 34

3. X-ray Crystallography structures.. 39

4. Spectroscopic data.. 40

5. Computational studies data.. 44

6. HRMS Data... 49

1. 1D NMR SPECTRA

BOPYPY 1

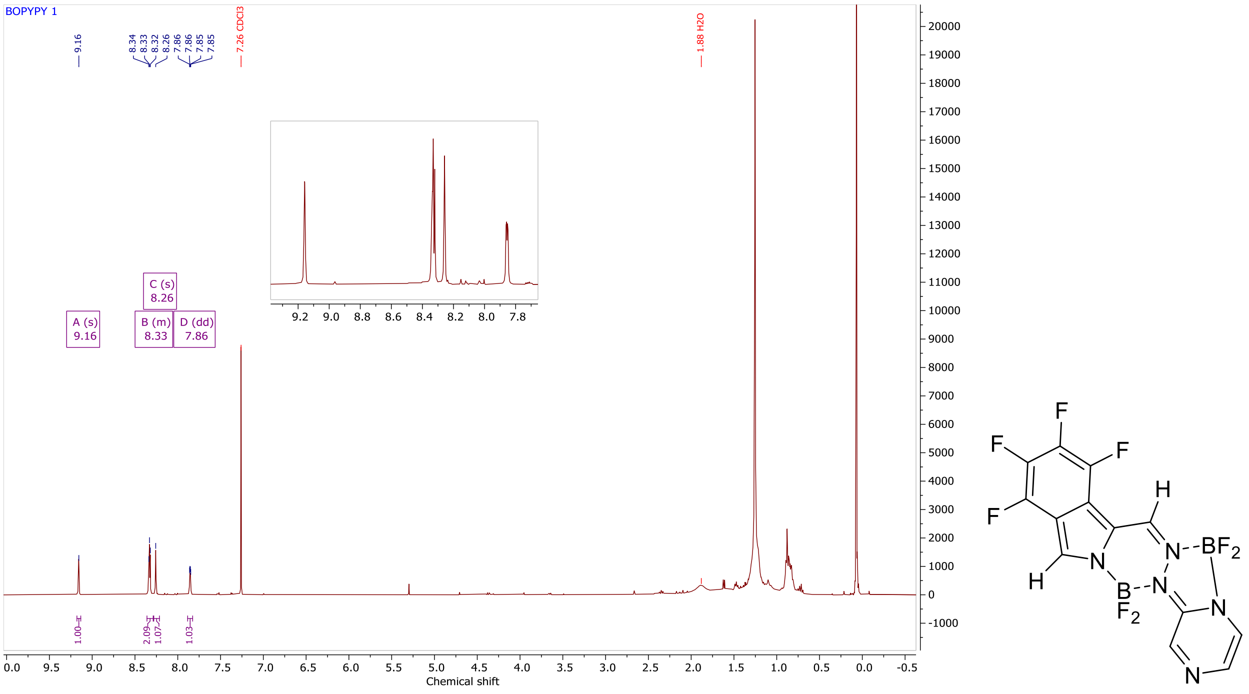

Figure S 1. ^{1}H NMR Spectrum

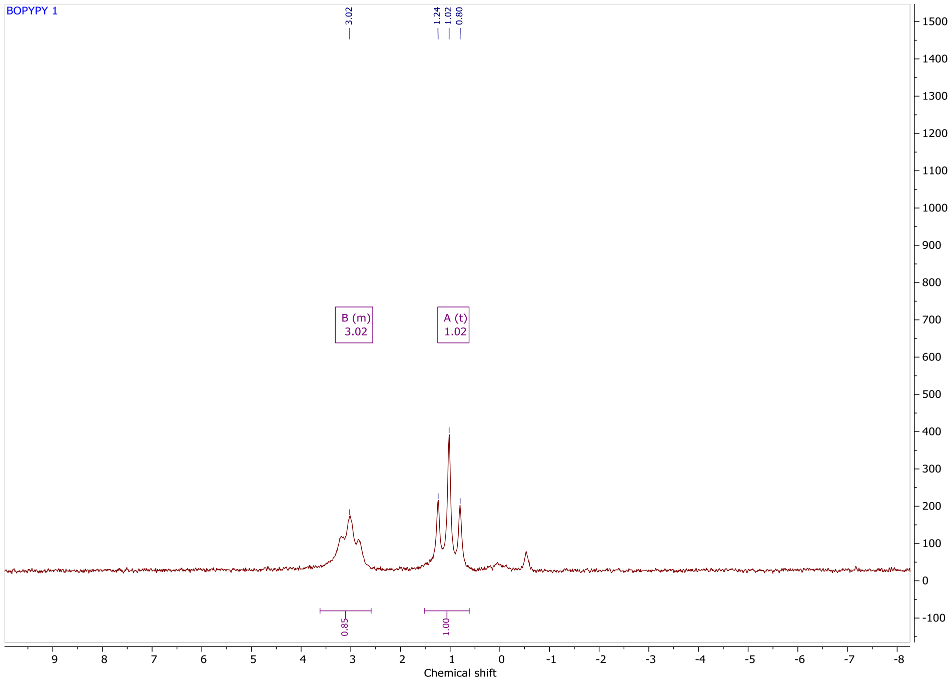

Figure S 2. ^{11}B NMR Spectrum

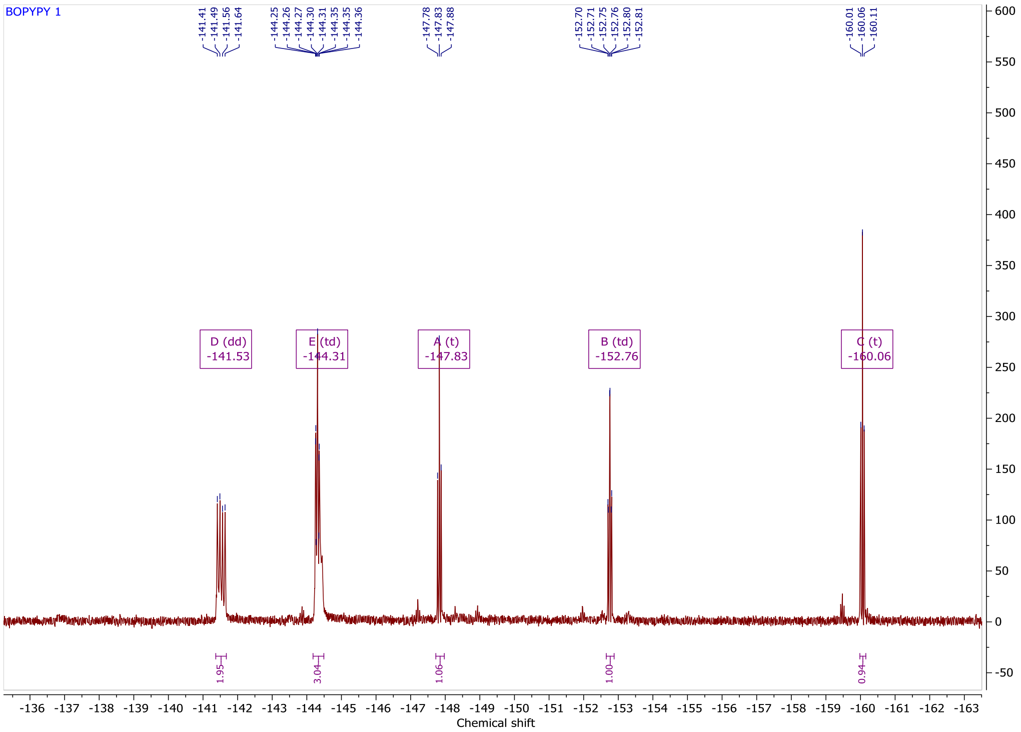

Figure S 3. ^{19}F NMR Spectrum

BOPYPY 2

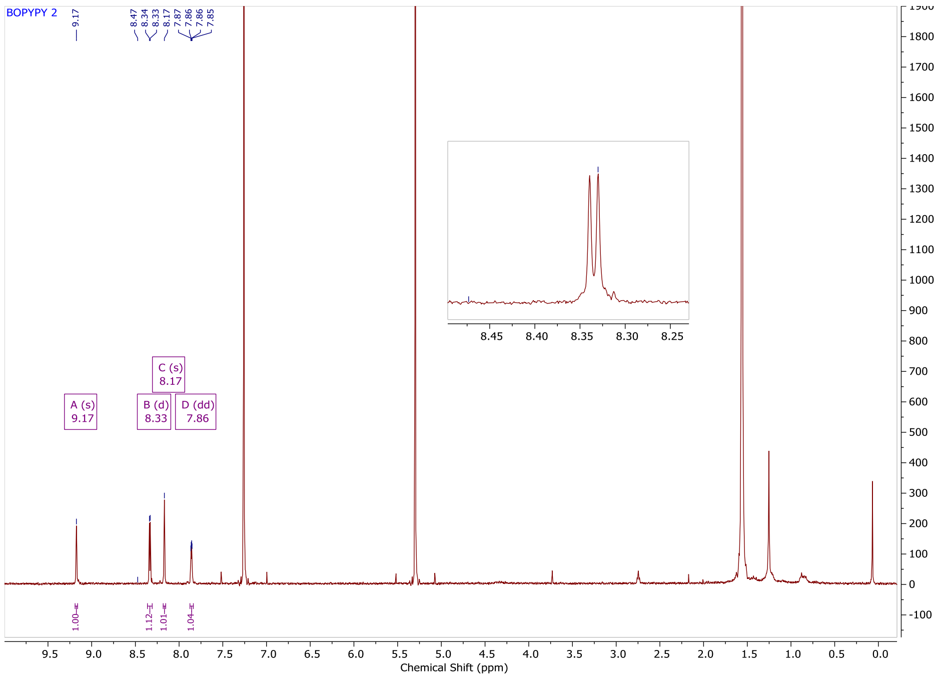

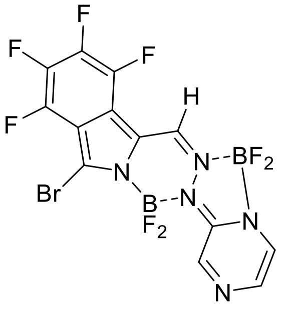

Figure S 4. ^{1}H NMR Spectrum

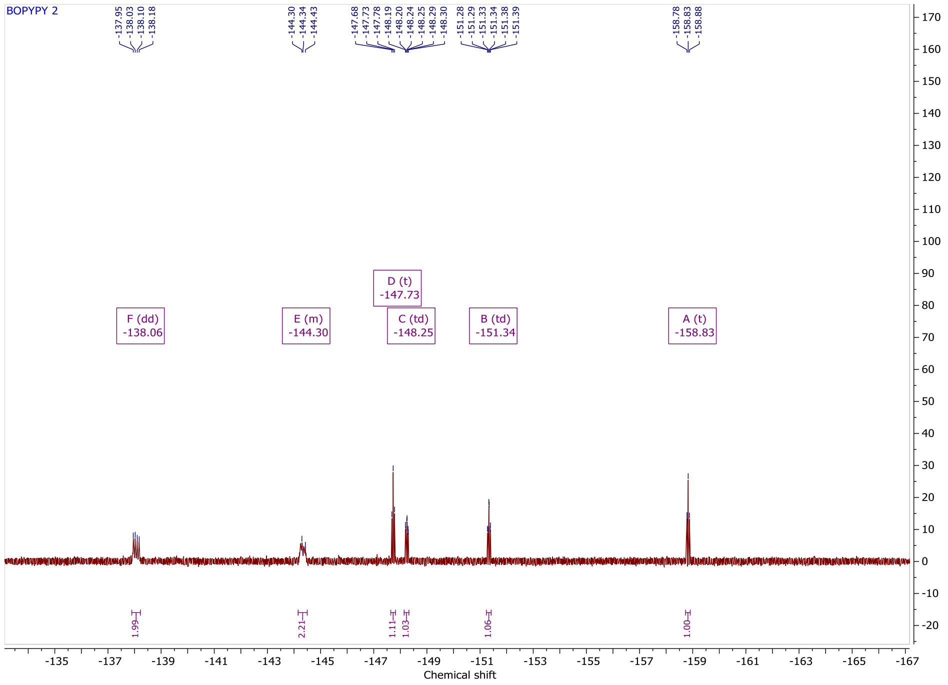

Figure S 5. ^{19}F NMR Spectrum

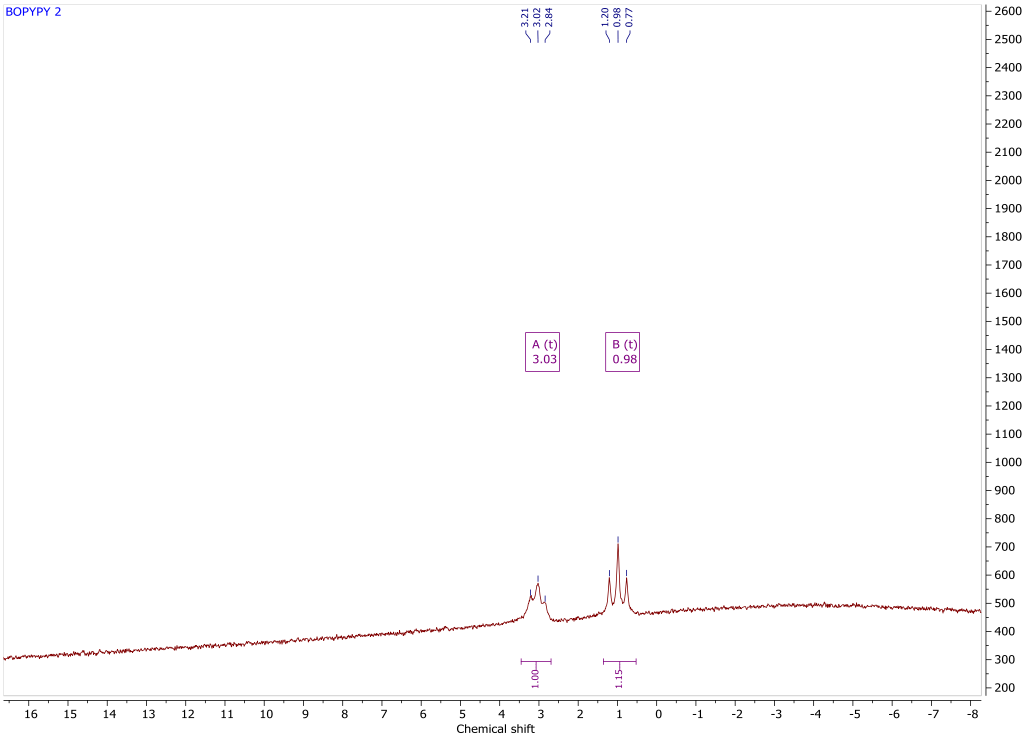

Figure S 6. ^{11}B NMR Spectrum

BOPYPY 3

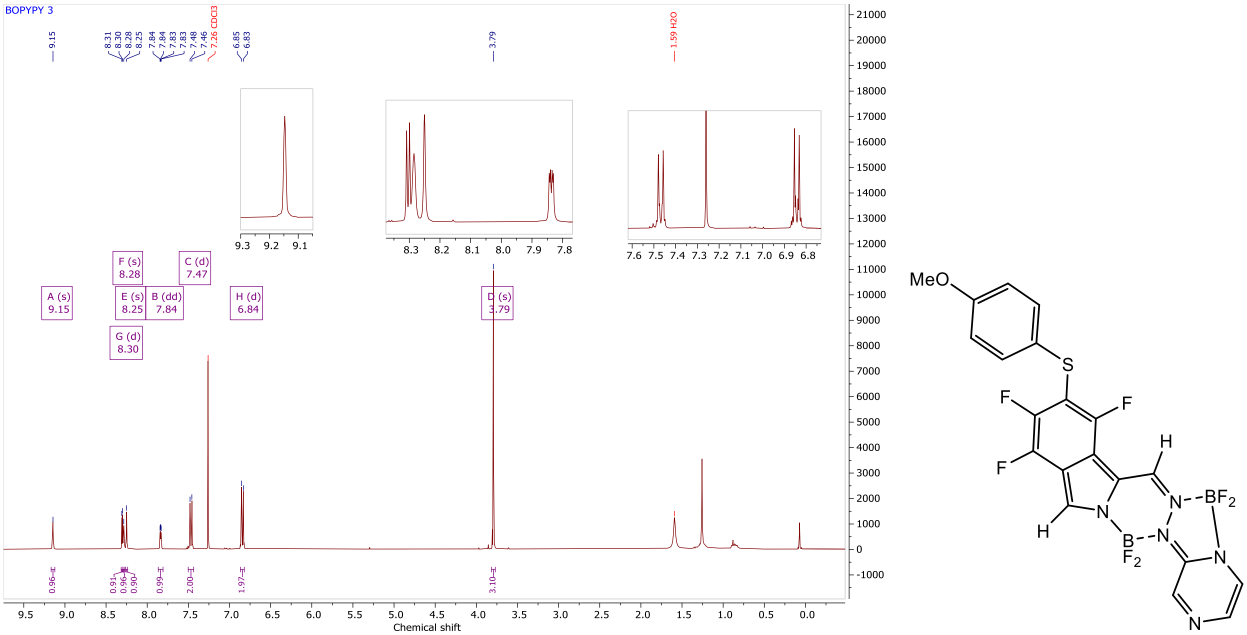

Figure S 7. ^{1}H NMR Spectrum

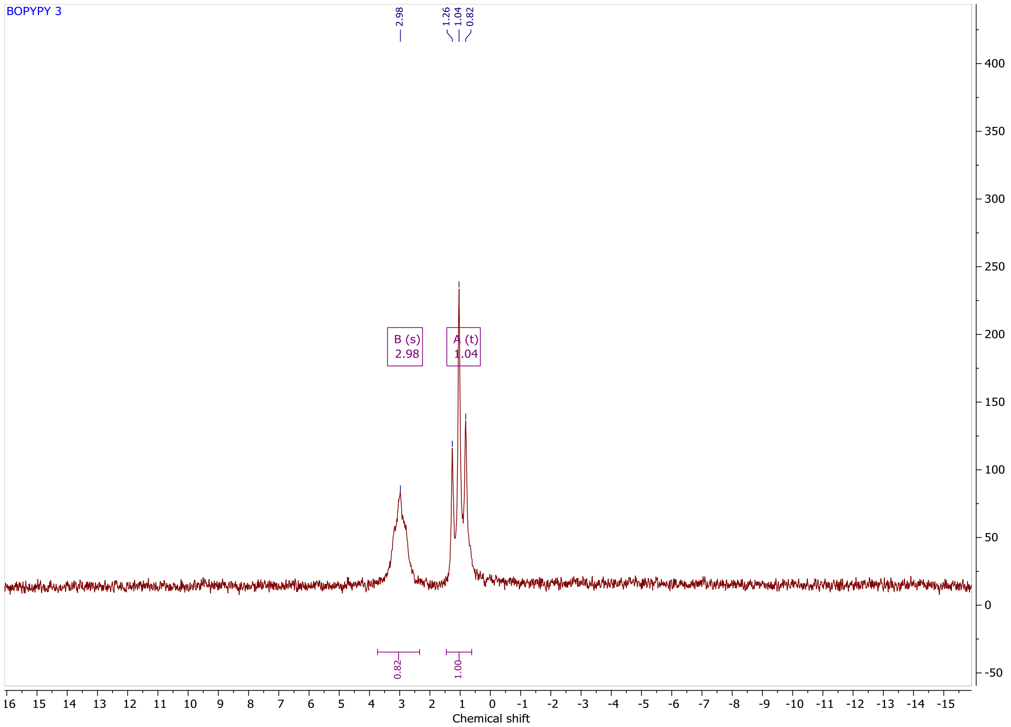

Figure S 8. ^{11}B NMR Spectrum

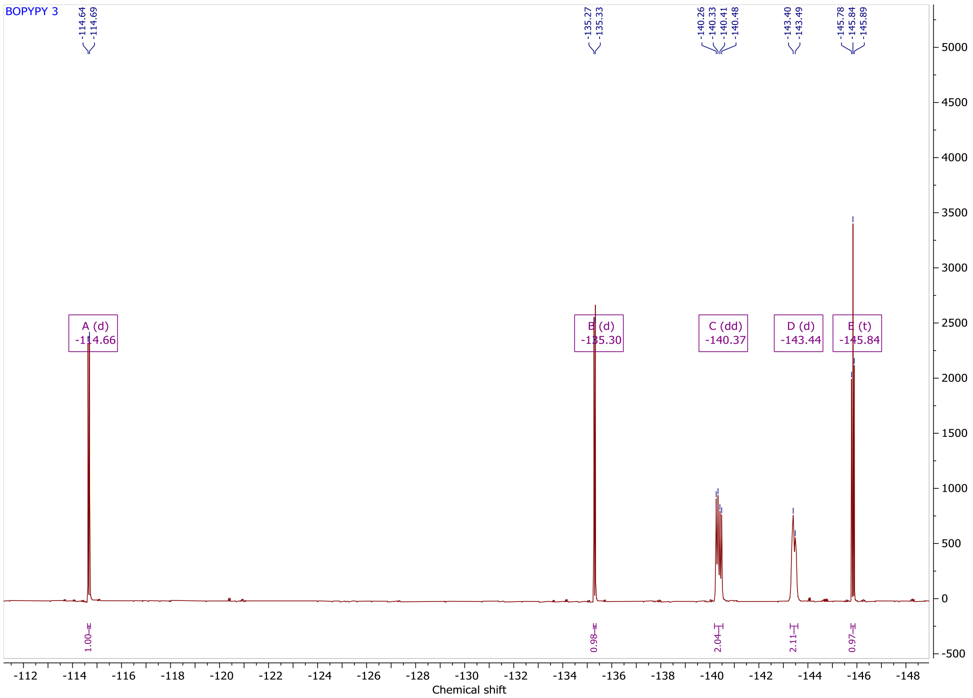

Figure S 9. ^{19}F NMR Spectrum

BOPYPY 4a

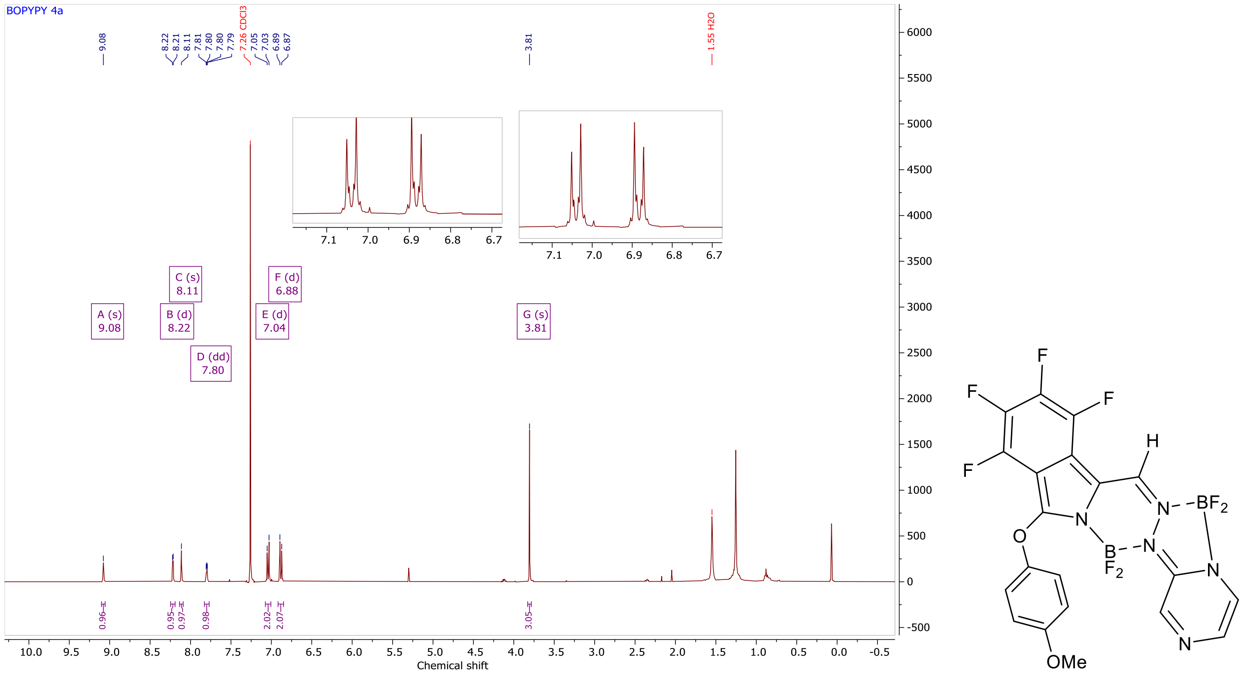

Figure S 10. ^{1}H NMR Spectrum

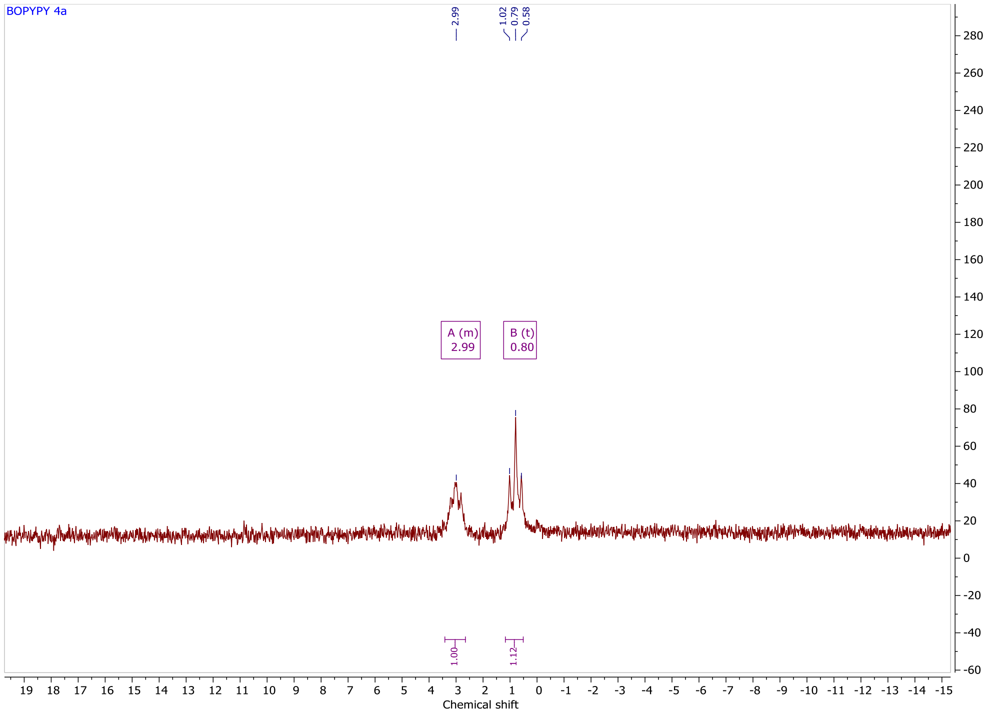

Figure S 11. ^{11}B NMR Spectrum

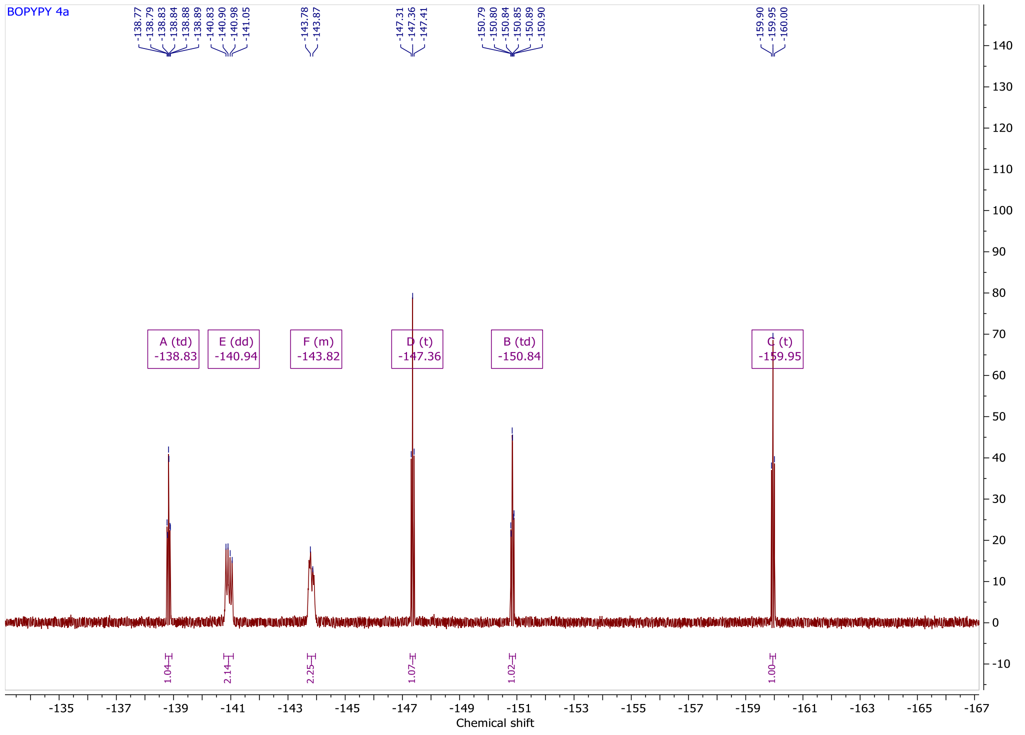

Figure S 12. ^{19}F NMR Spectrum

BOPYPY 4b

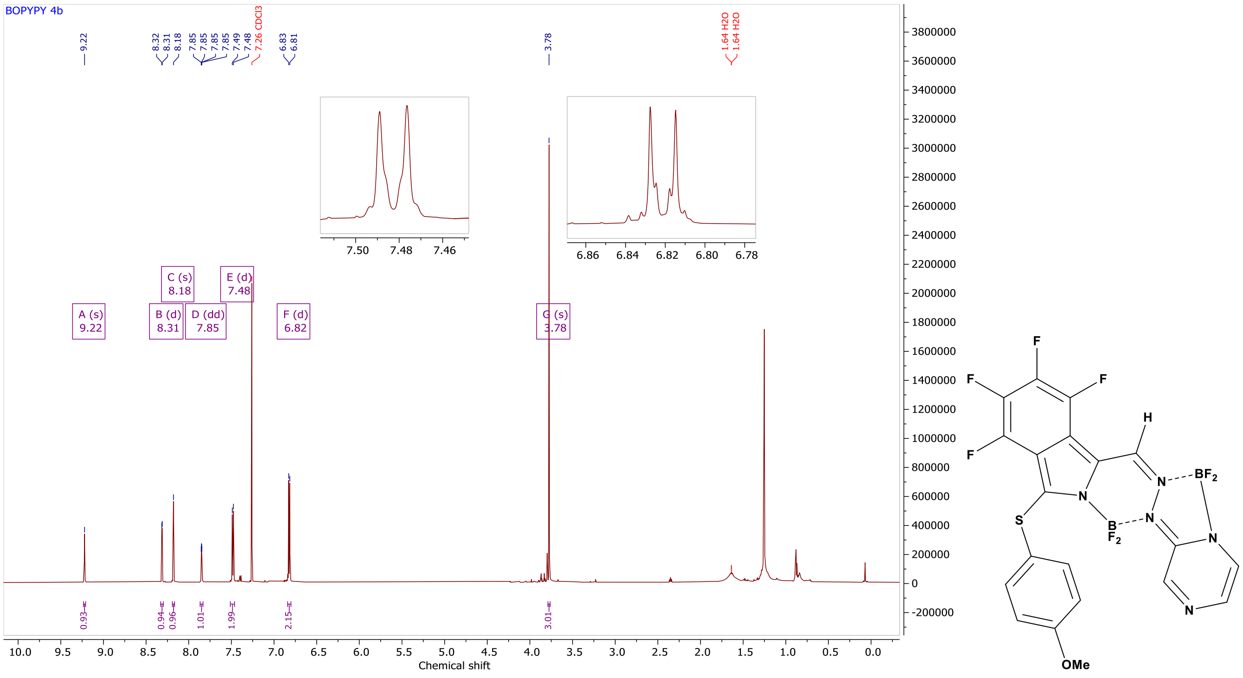

Figure S 13. ¹H NMR Spectrum

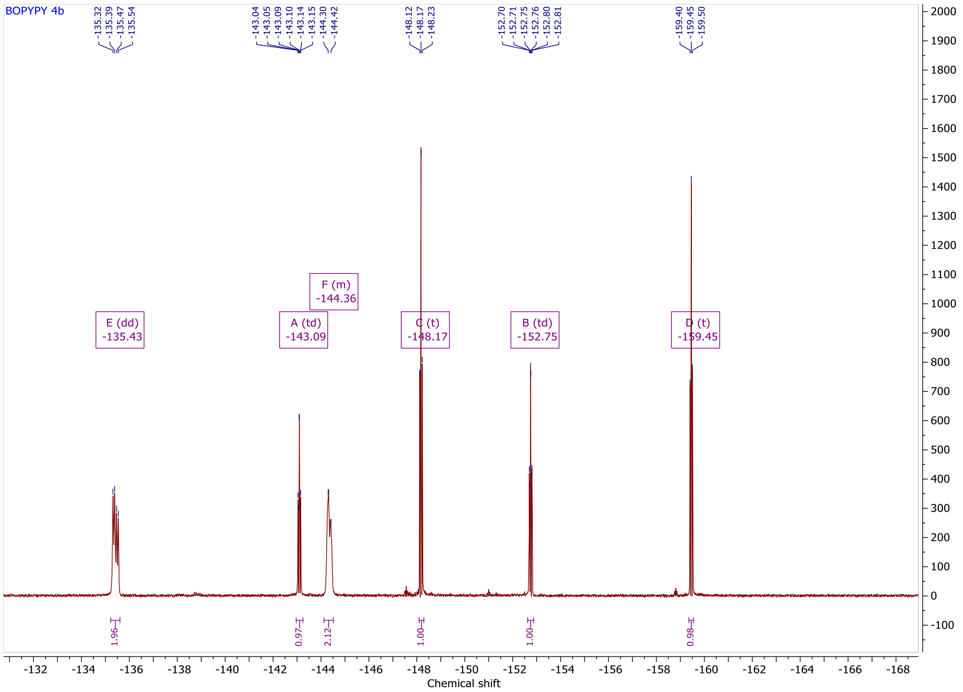

Figure S 14. ¹⁹F NMR Spectrum

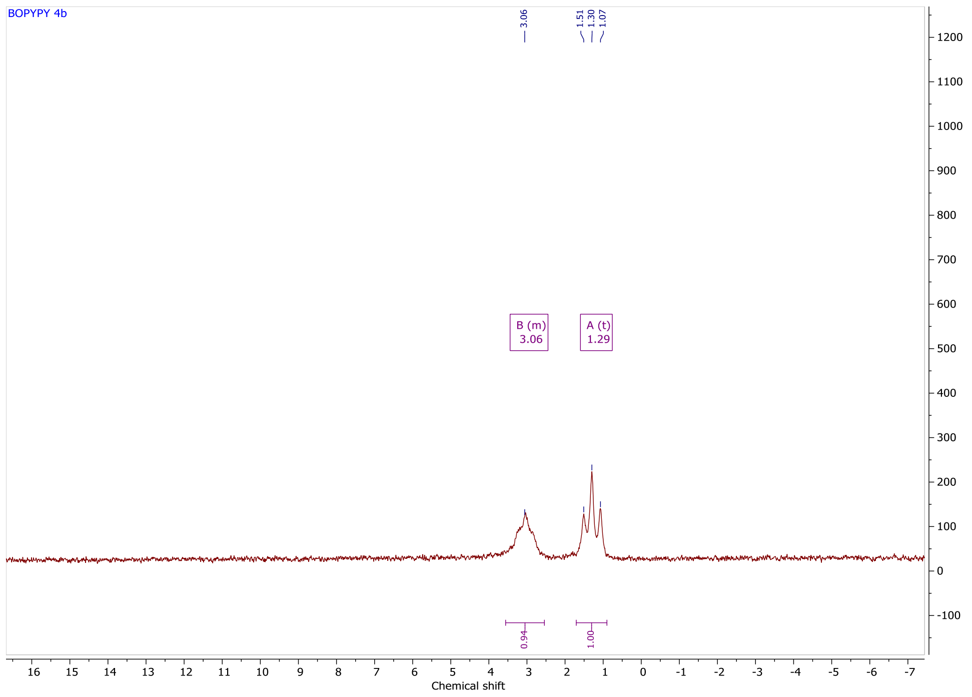

Figure S 15. ^{11}B NMR Spectrum

BOPYPY 4c

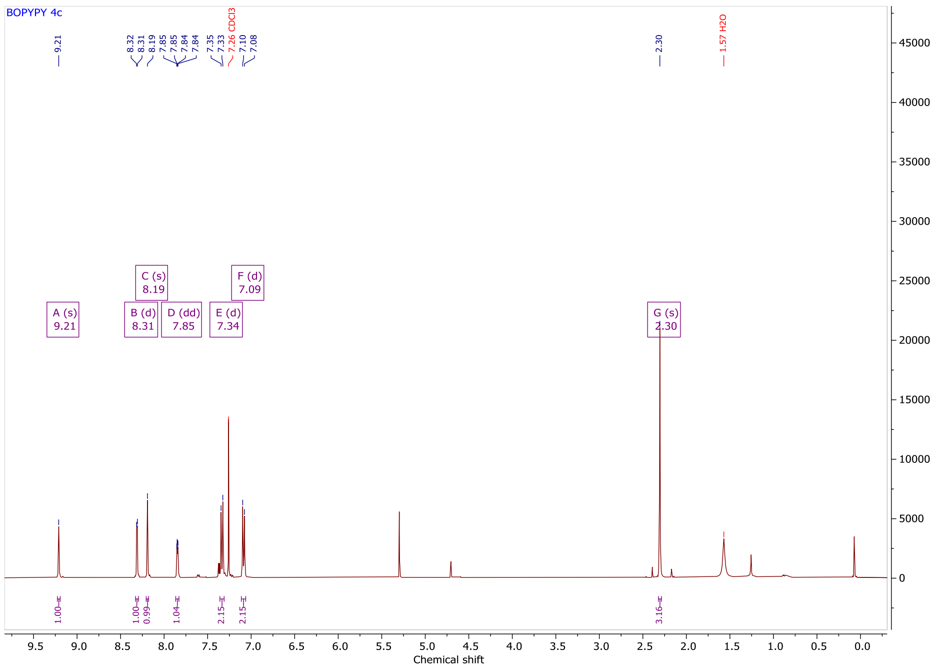

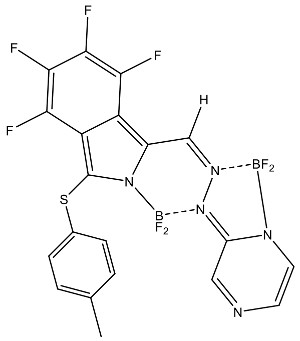

Figure S 16. ^{1}H NMR Spectrum

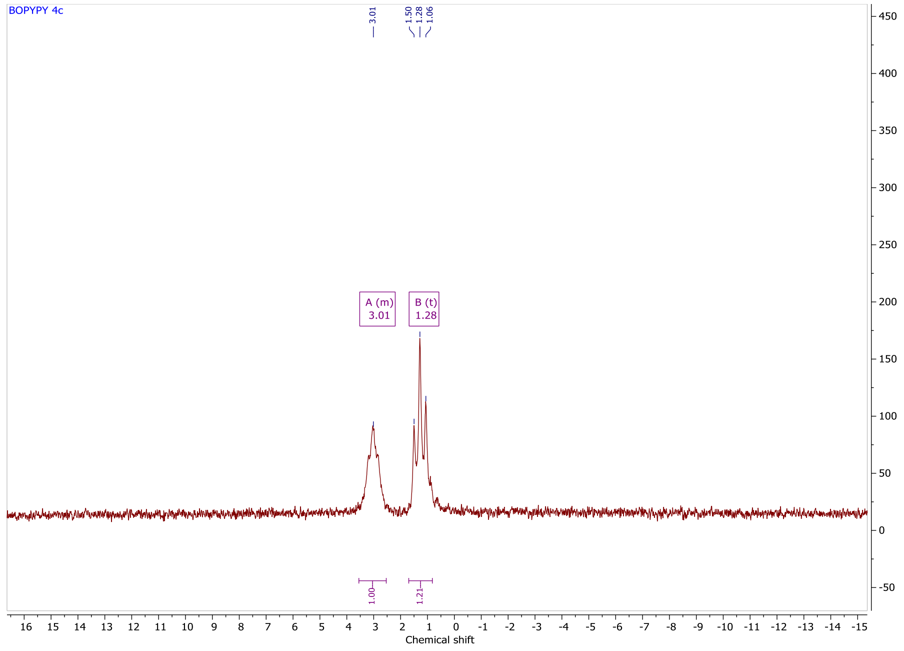

Figure S 17. ^{11}B NMR Spectrum

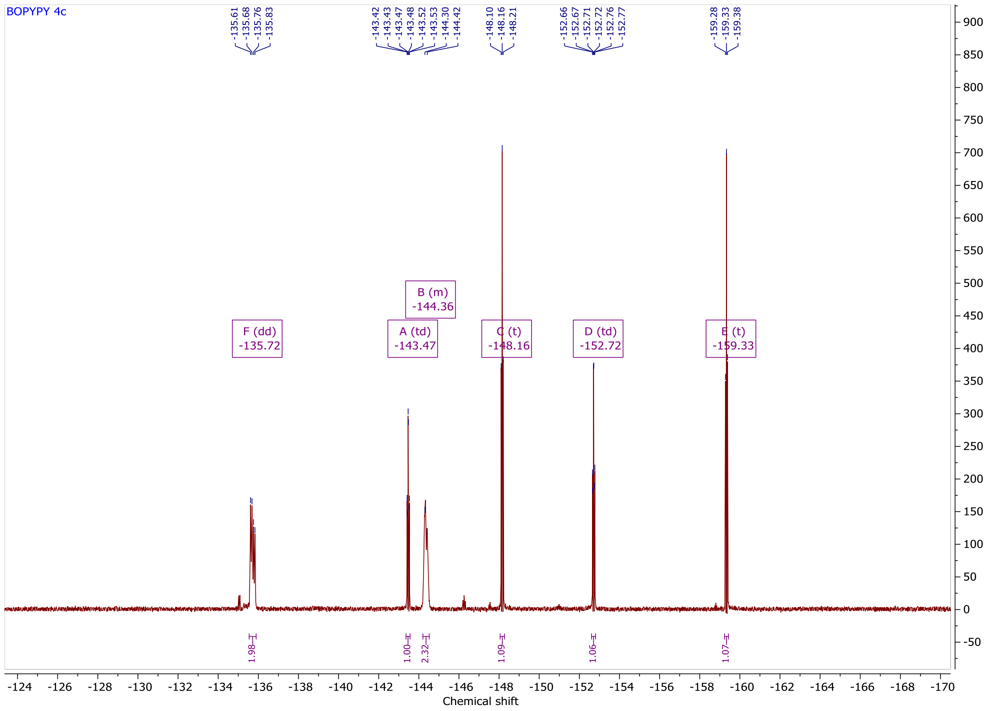

Figure S 18. ^{19}F NMR Spectrum

BOPYPY 4d

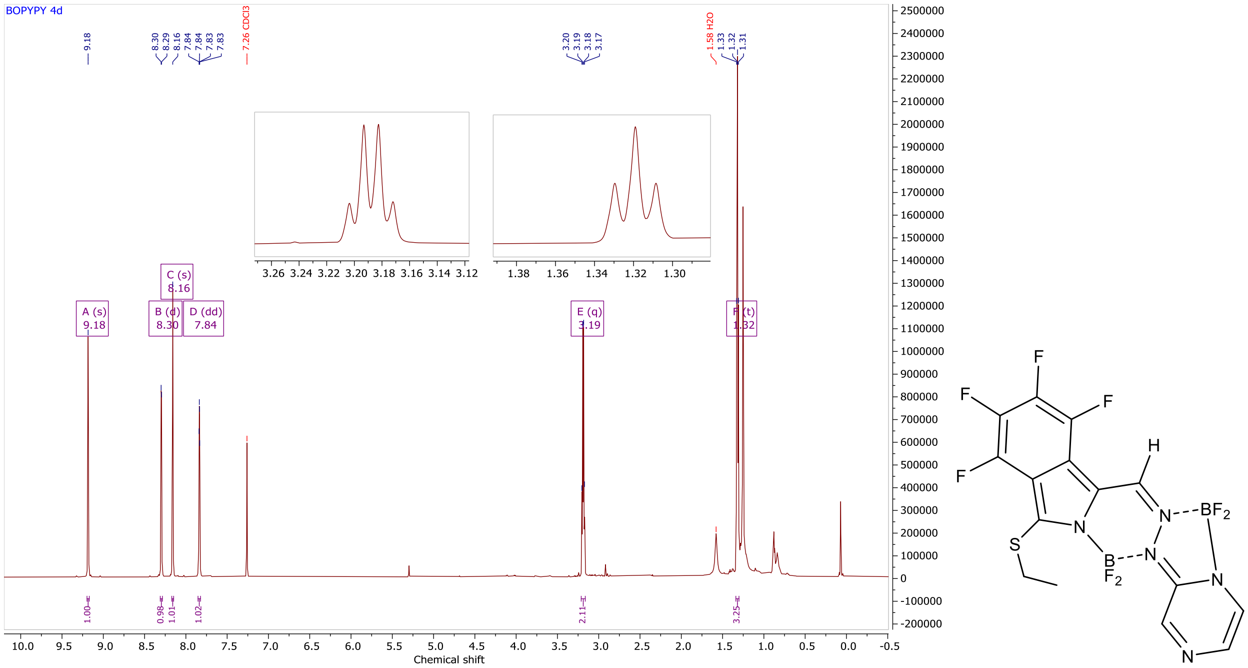

Figure S 19. ^{1}H NMR Spectrum

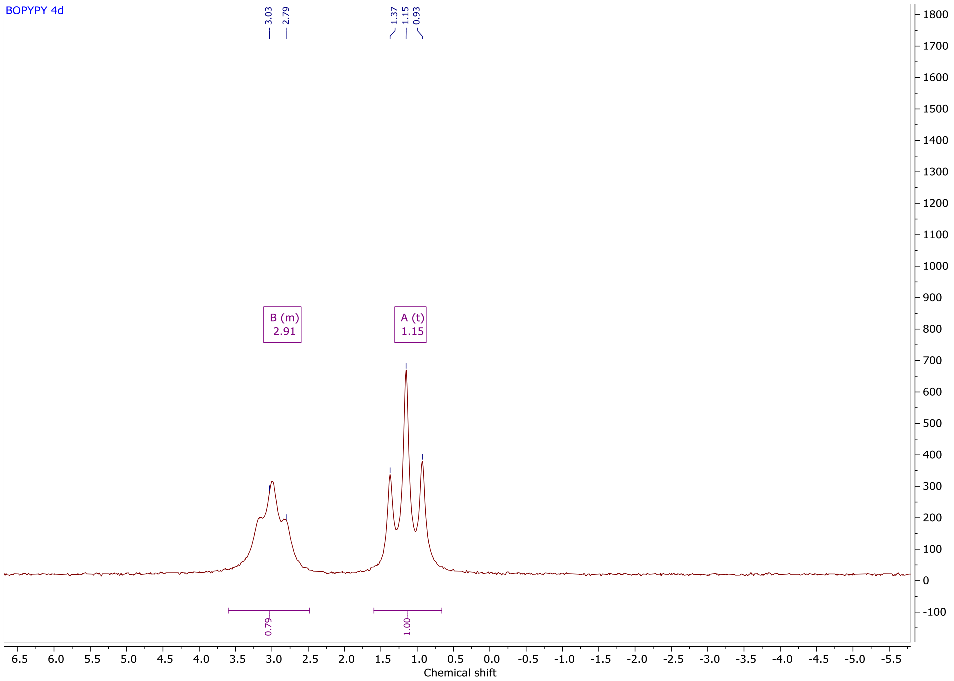

Figure S 20. ^{11}B NMR Spectrum

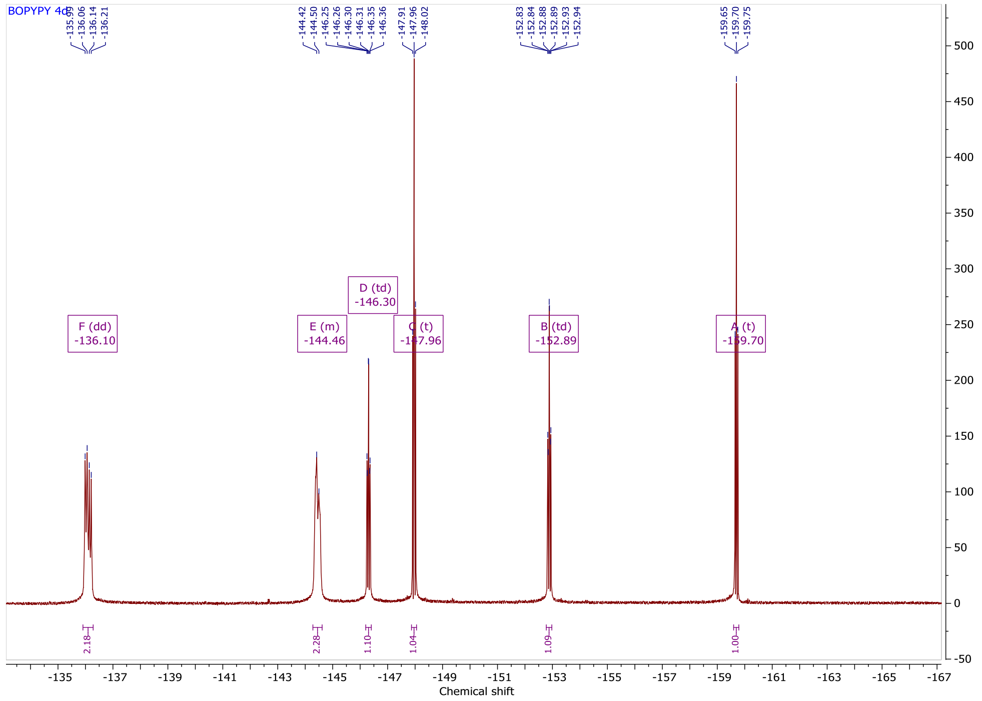

Figure S 21. ^{19}F NMR Spectrum

BOPYPY 4e

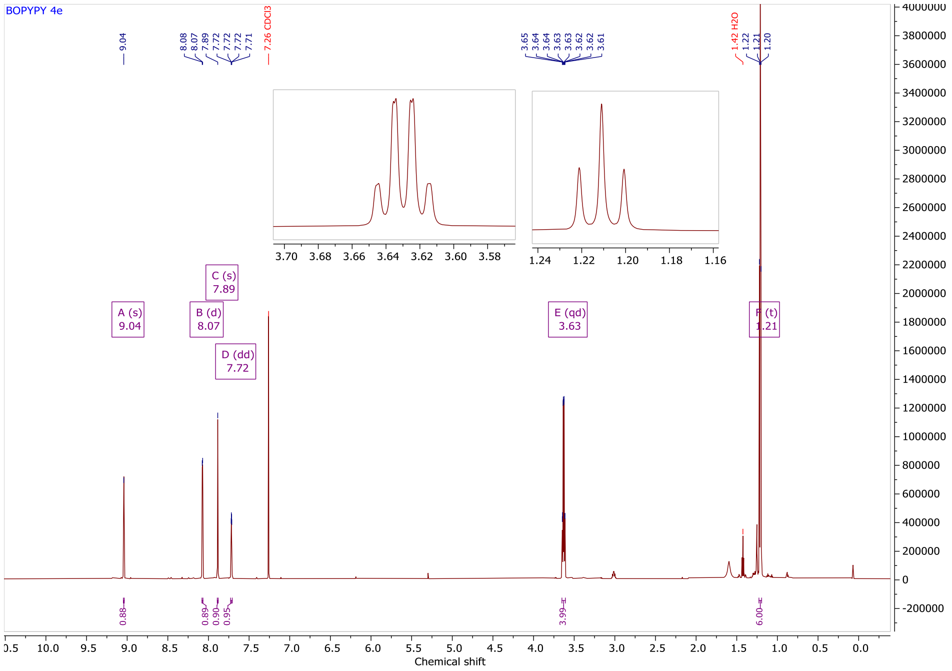

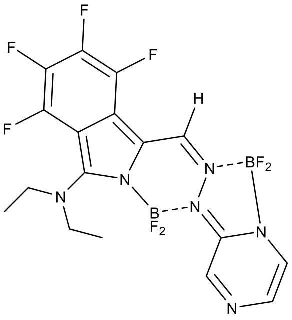

Figure S 22. ^{1}H NMR Spectrum

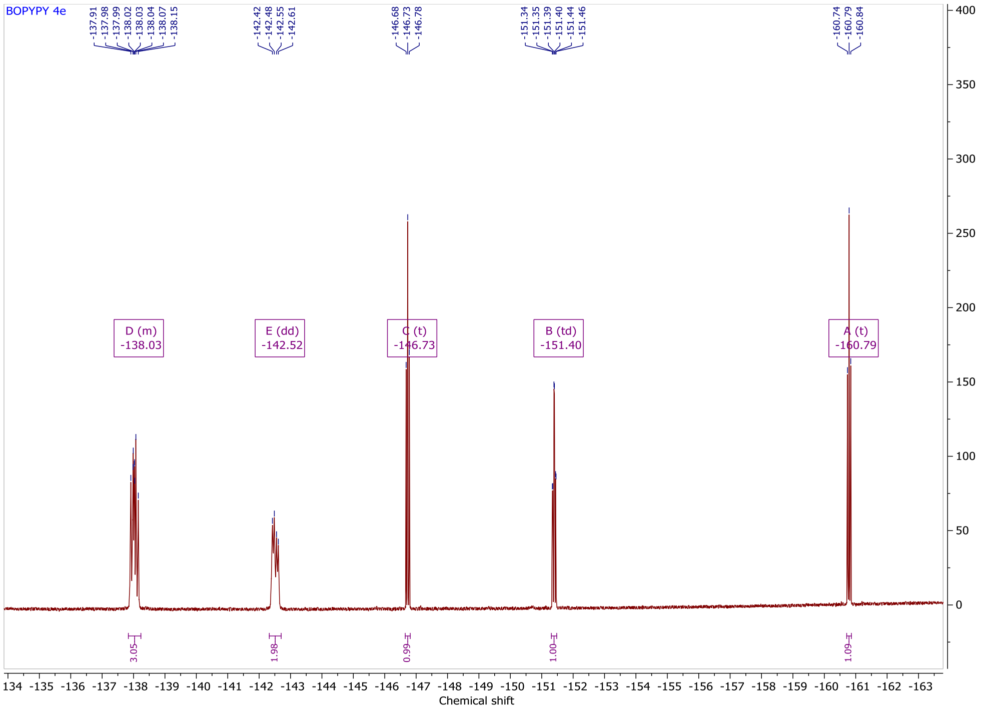

Figure S 23. ^{19}F NMR Spectrum

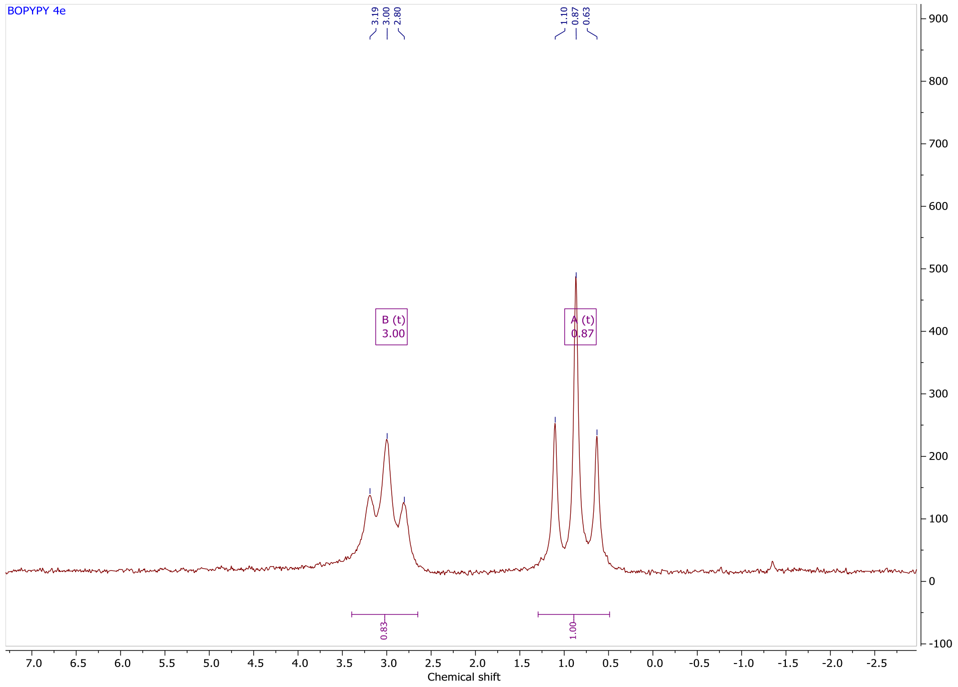

Figure S 24. ^{11}B NMR Spectrum

BOPYPY 4f

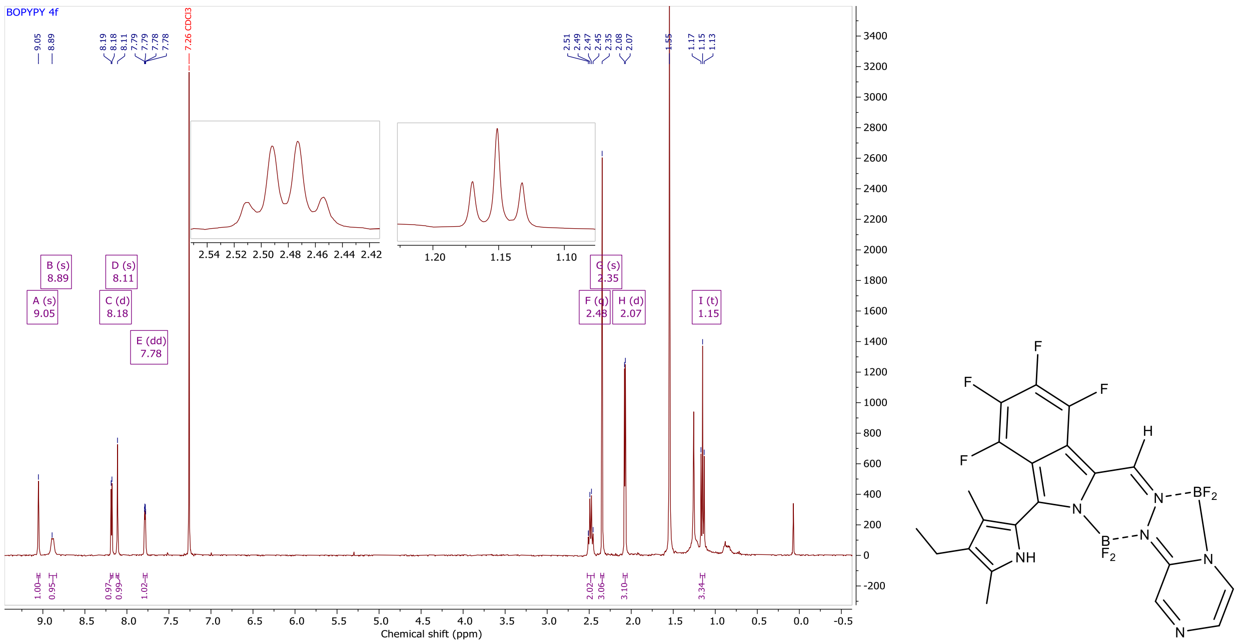

Figure S 25. ^{1}H NMR Spectrum

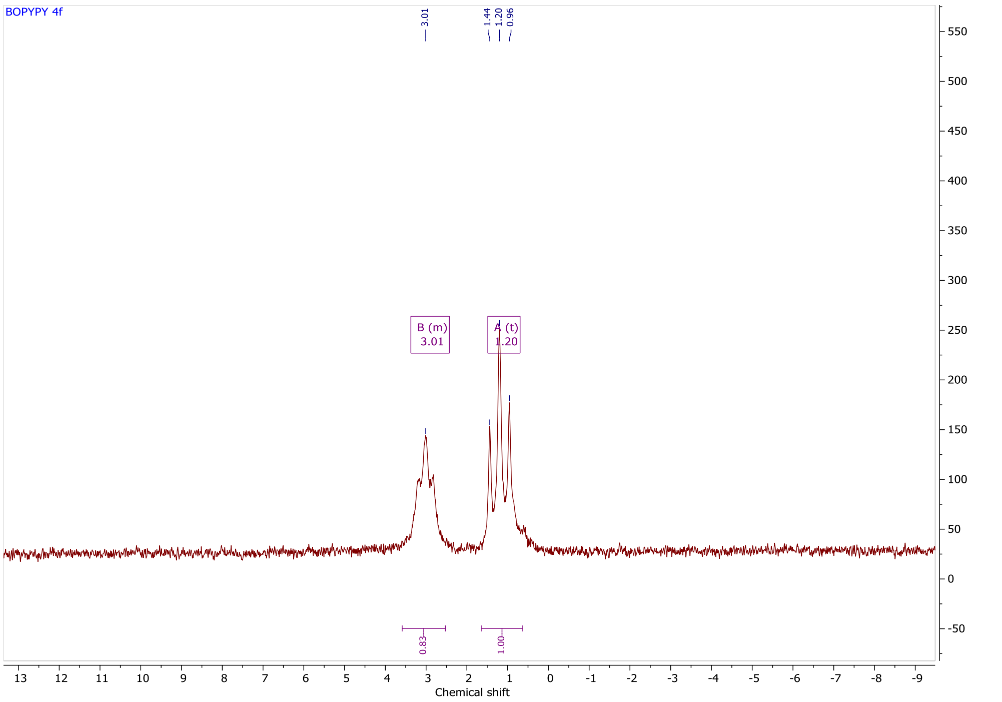

Figure S 26. ^{11}B NMR Spectrum

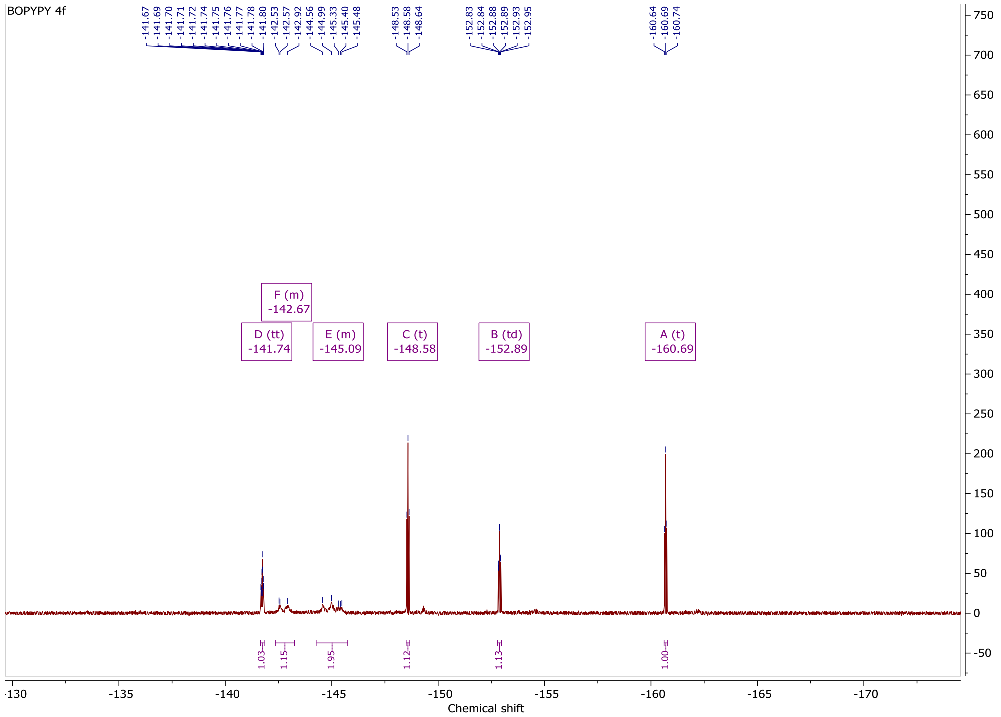

Figure S 27. ^{19}F NMR Spectrum

BOPYPY 4g

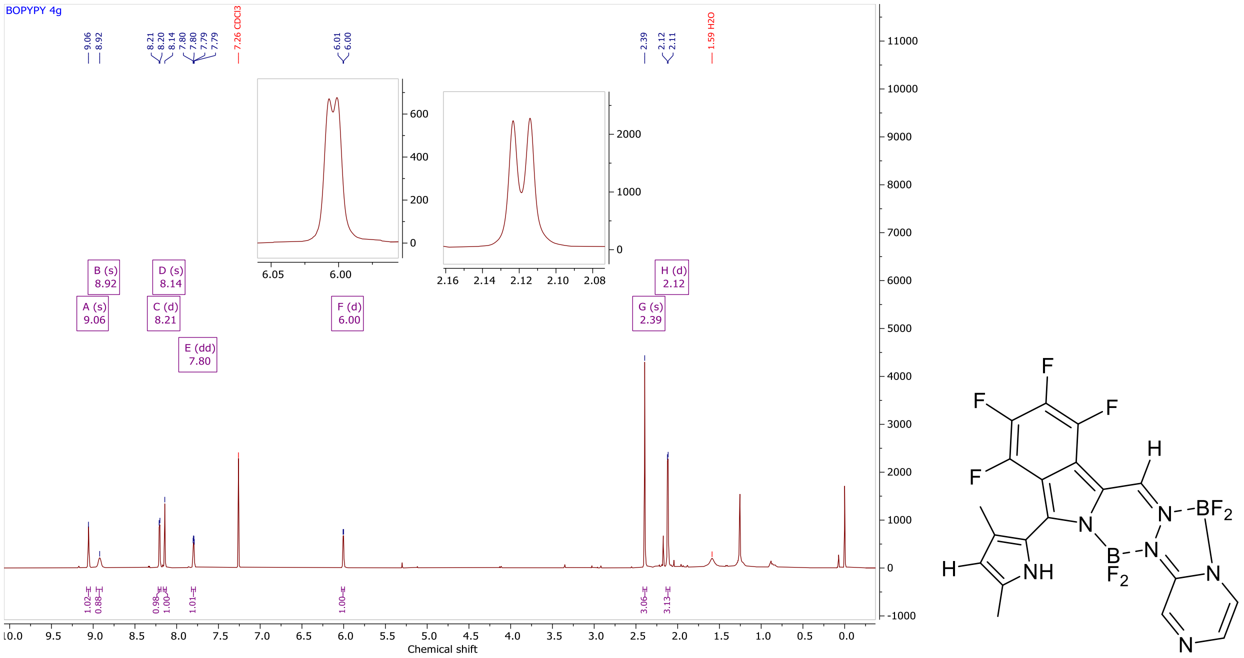

Figure S 28. ^{1}H NMR Spectrum

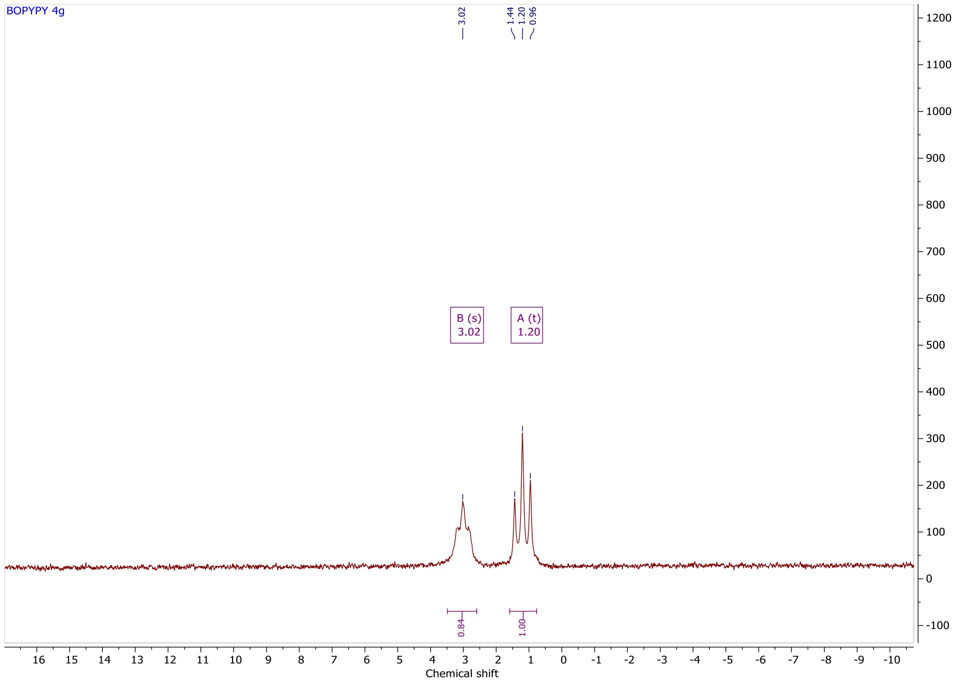

Figure S 29. ^{11}B NMR Spectrum

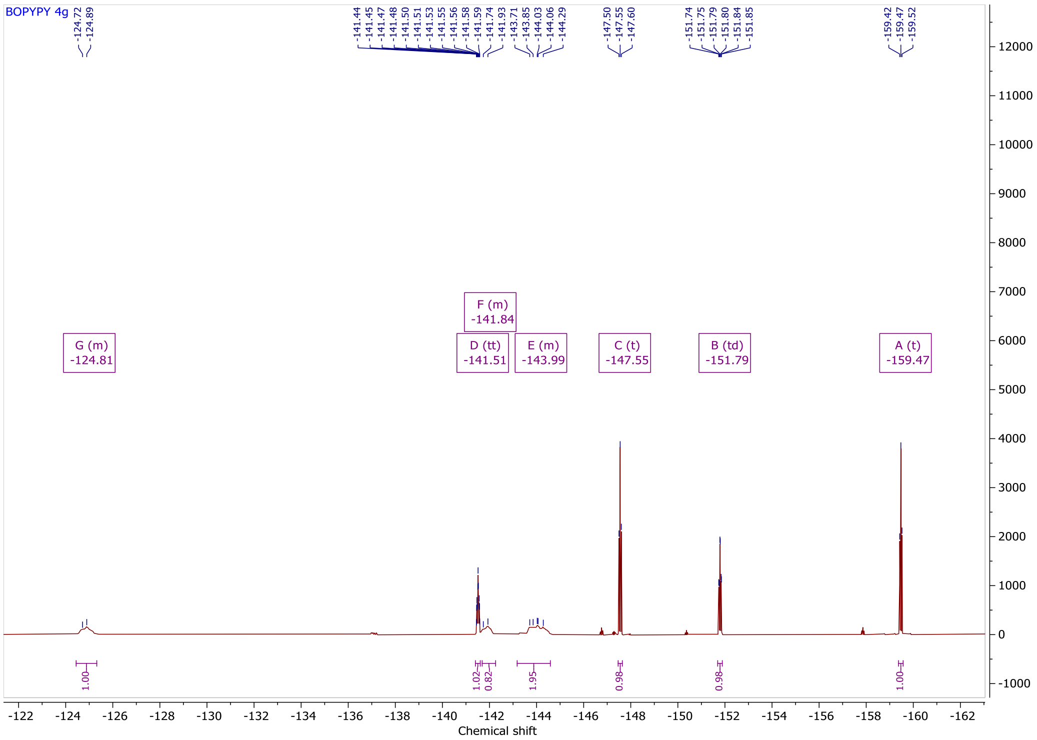

Figure S 30. ^{19}F NMR Spectrum

BOPYPY 5a

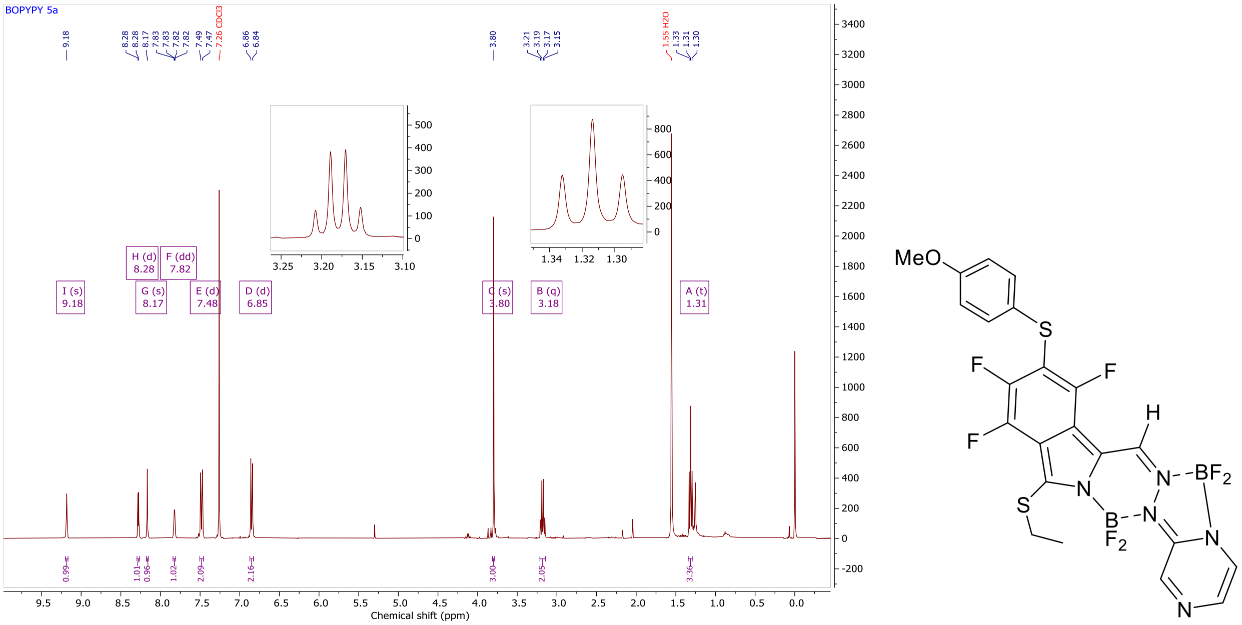

Figure S 31. ^{1}**H NMR Spectrum**

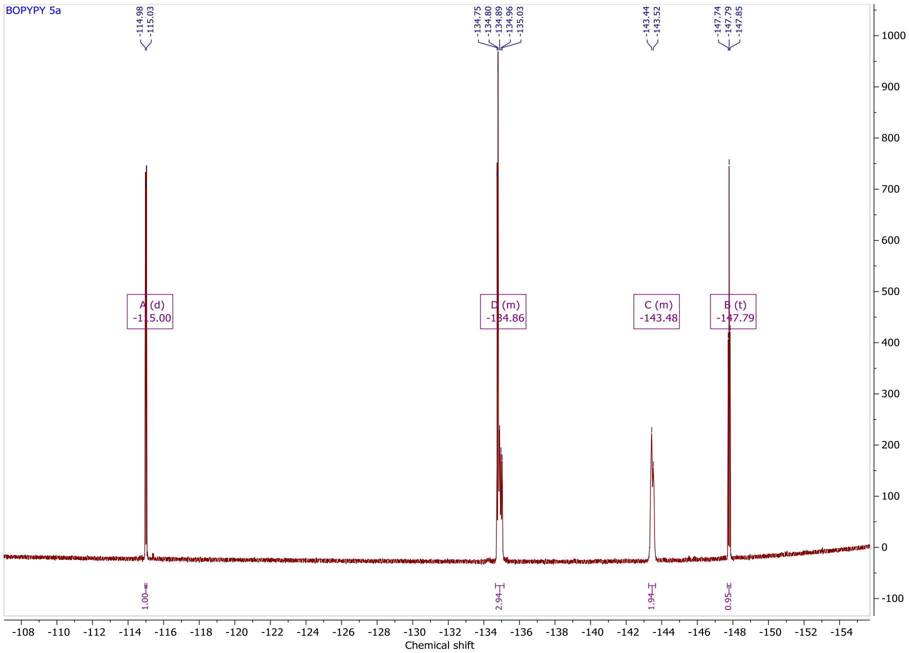

Figure S 32. ^{19}**F NMR Spectrum**

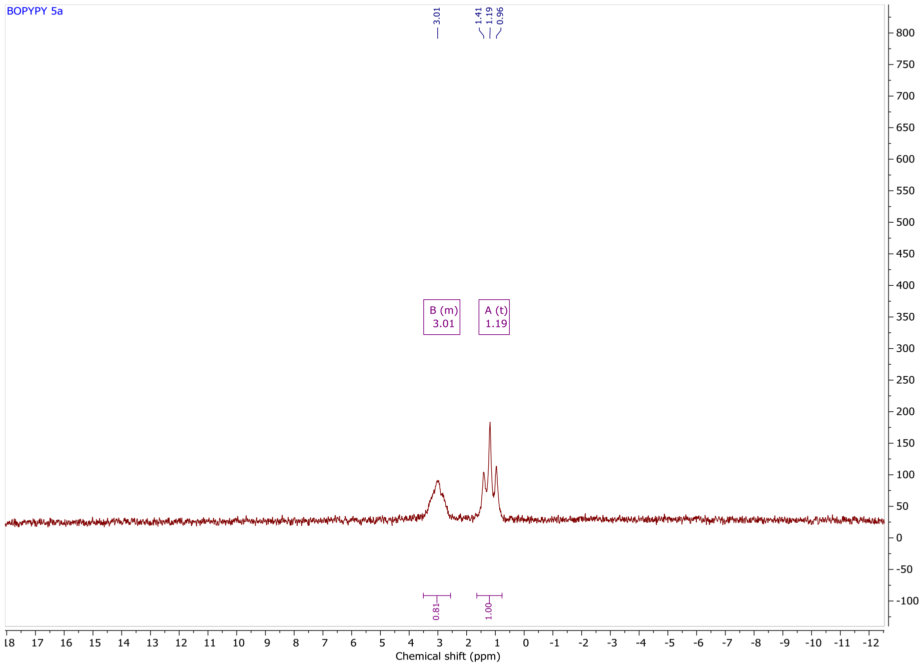

Figure S 33. ^{11}B NMR Spectrum

BOPYPY 5b

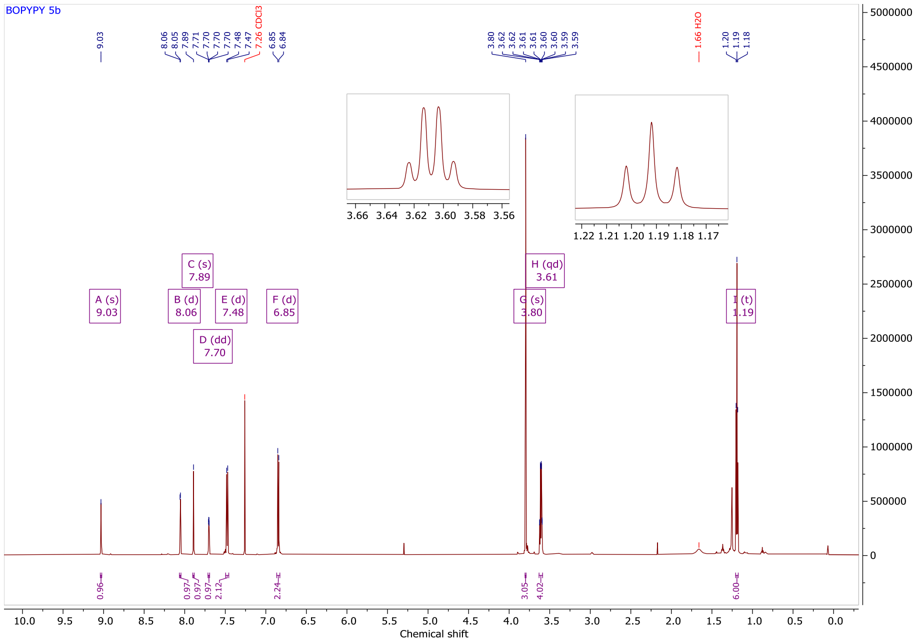

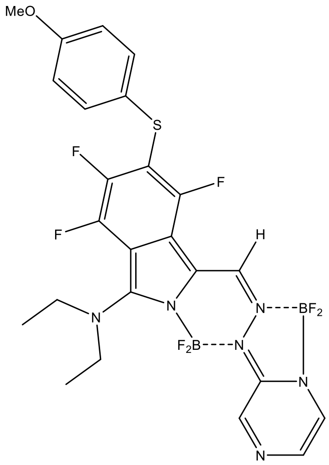

Figure S 34. ^{1}H NMR Spectrum

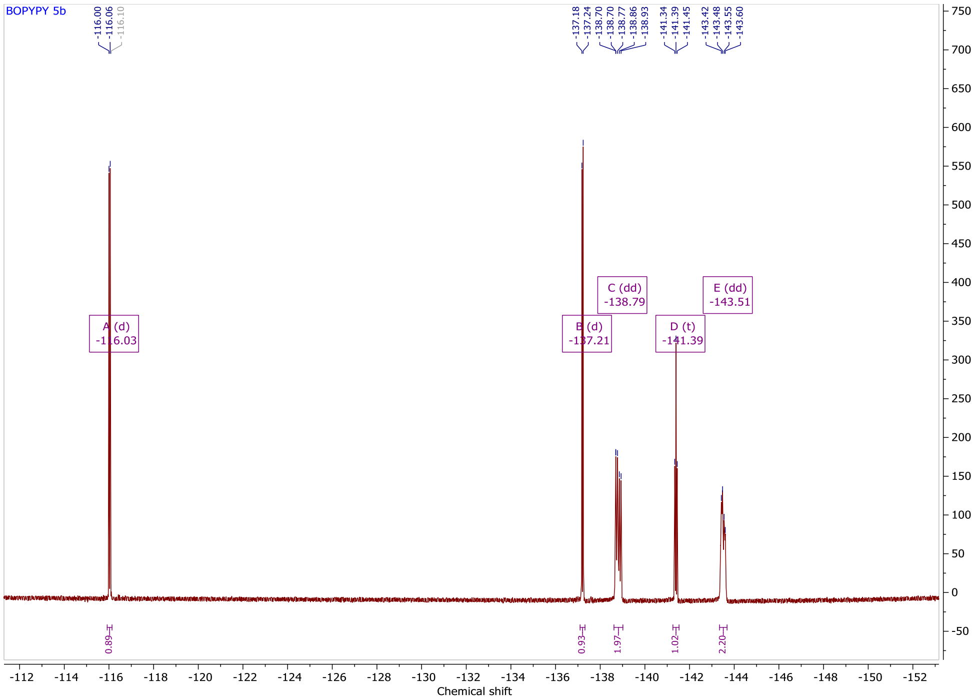

Figure S 35. ^{19}F NMR Spectrum

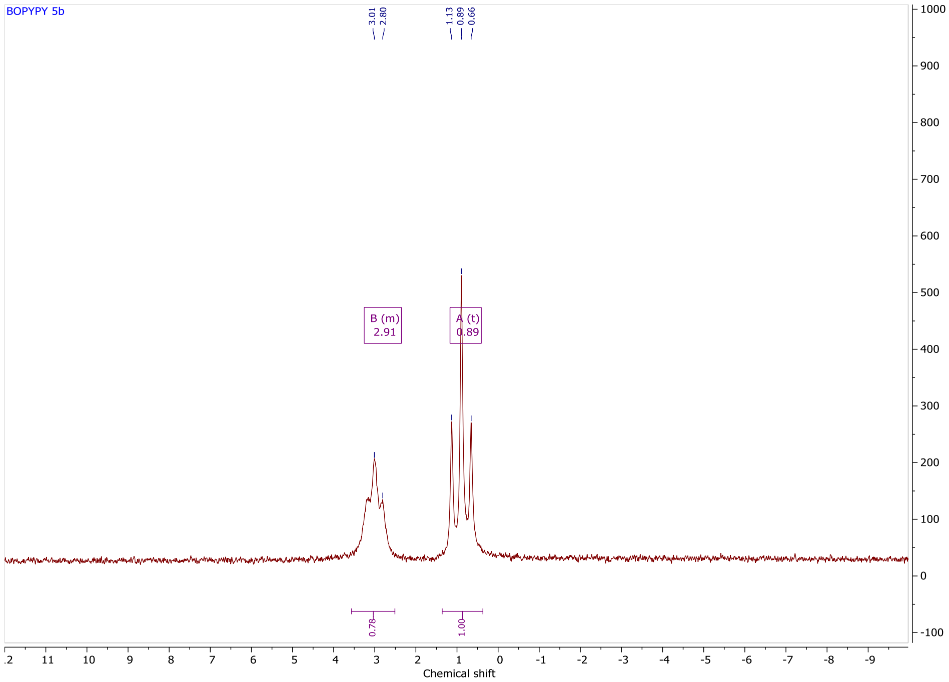

Figure S 36. ^{11}B NMR Spectrum

BOPYPY 5c

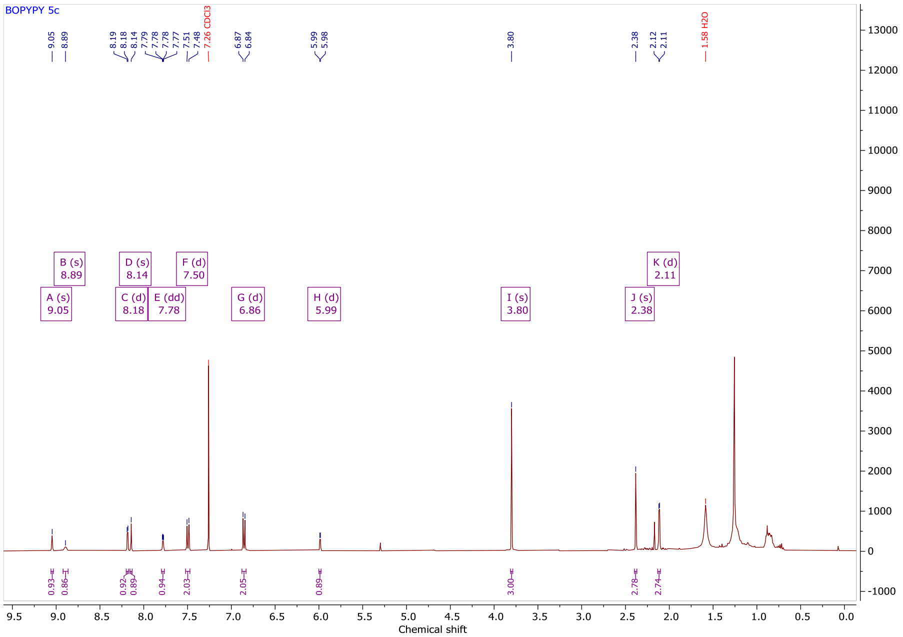

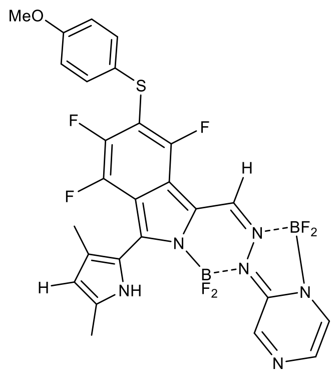

Figure S 37. ^{1}H NMR Spectrum

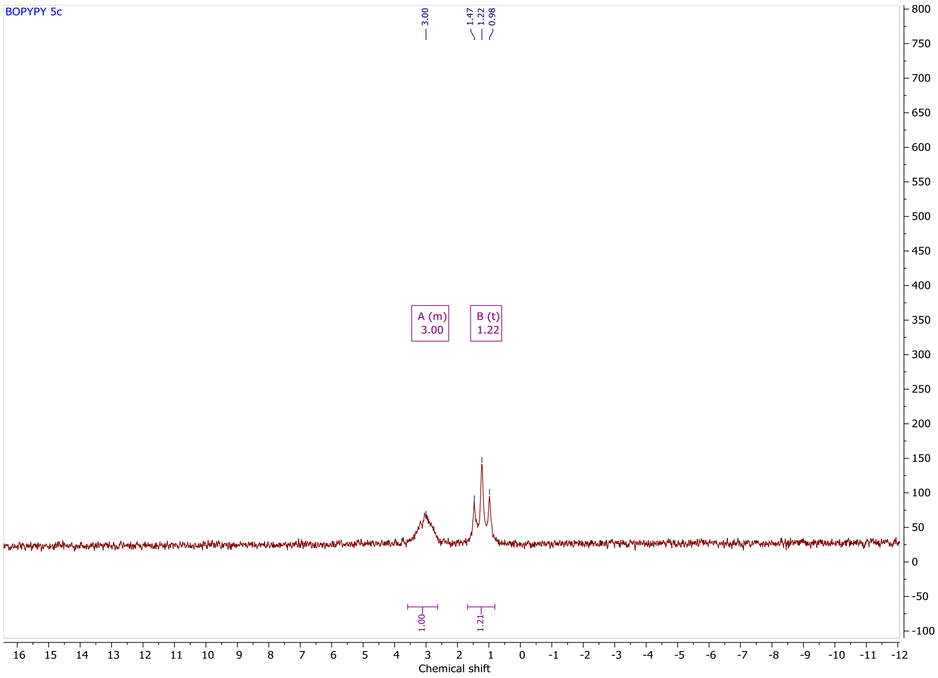

Figure S 38. ^{11}B NMR Spectrum

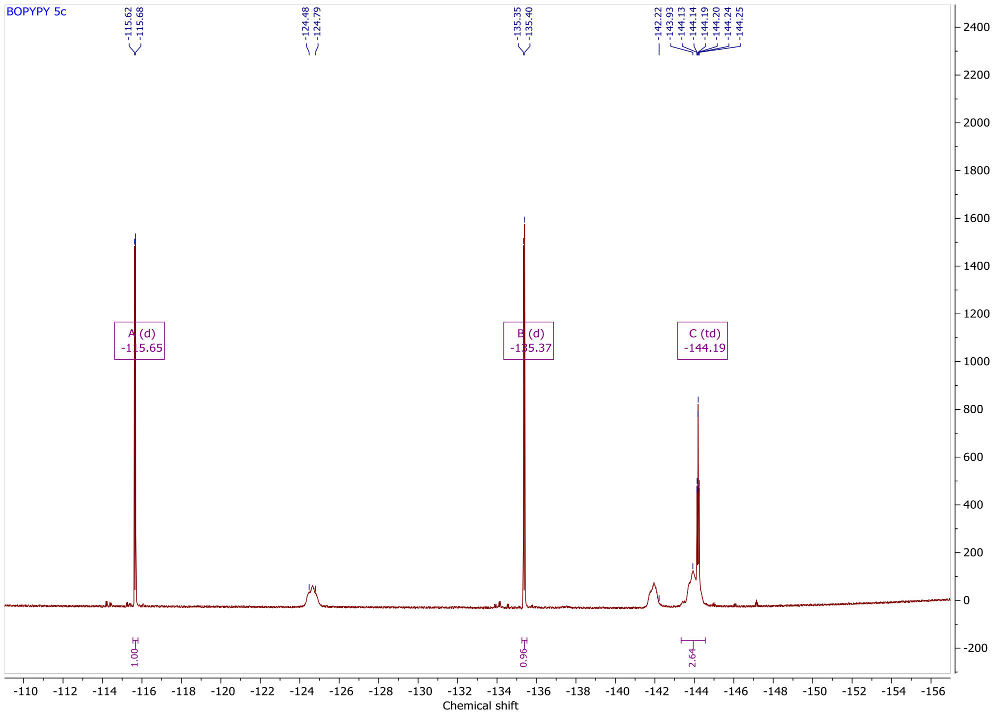

Figure S 39. ^{19}F NMR Spectrum

BOPYPY 6

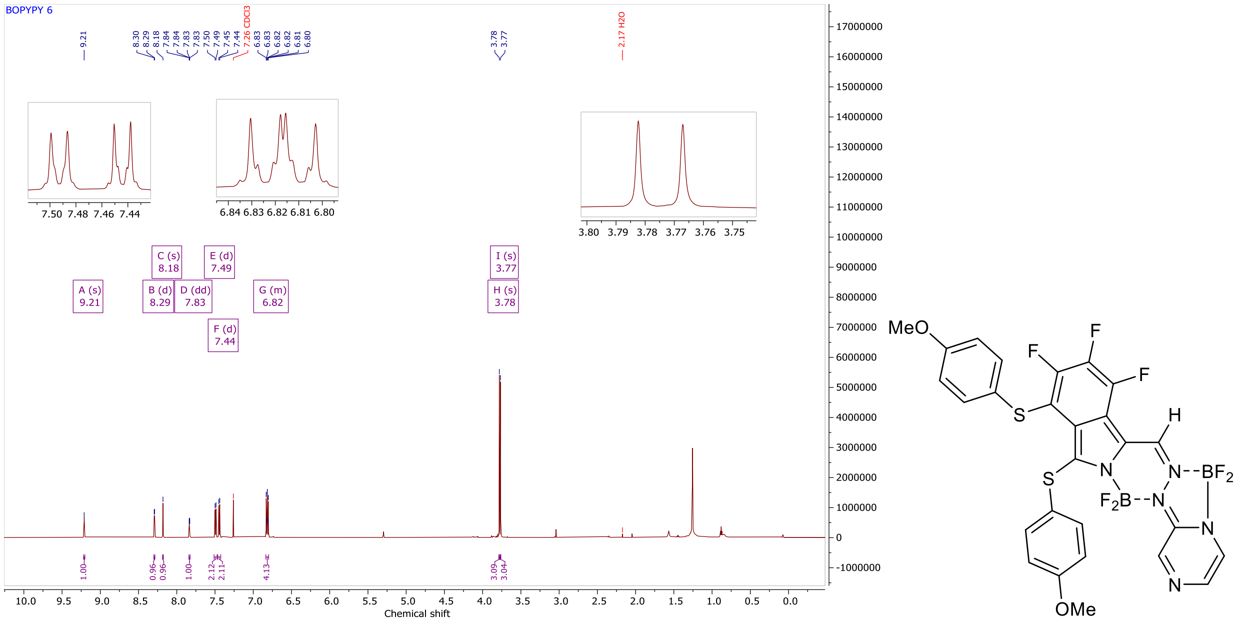

Figure S 40. ^{1}H NMR Spectrum

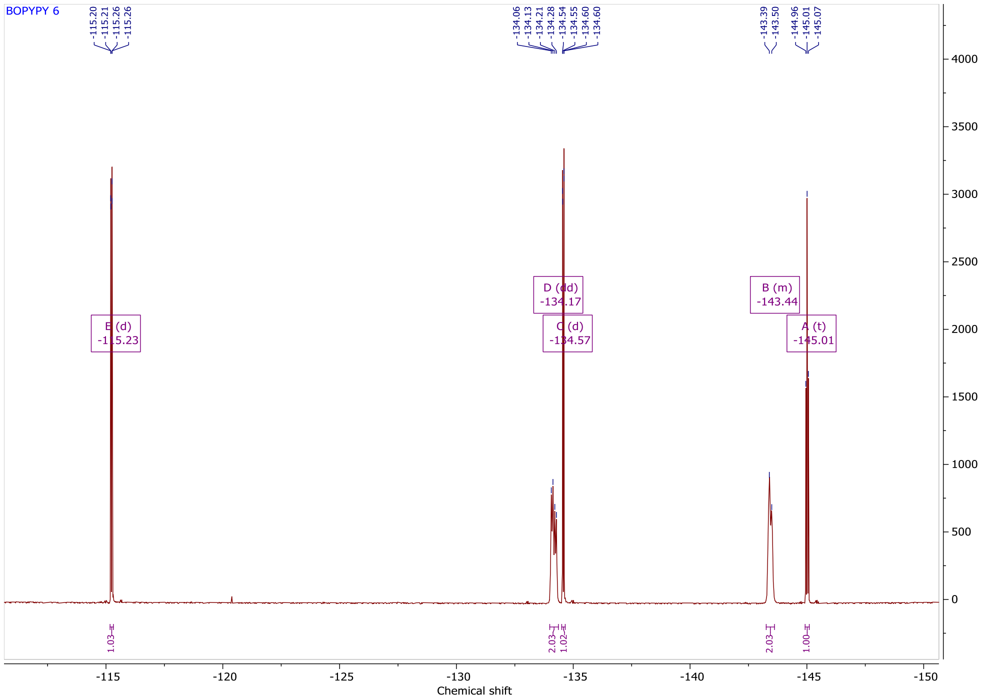

Figure S 41. ^{19}F NMR Spectrum

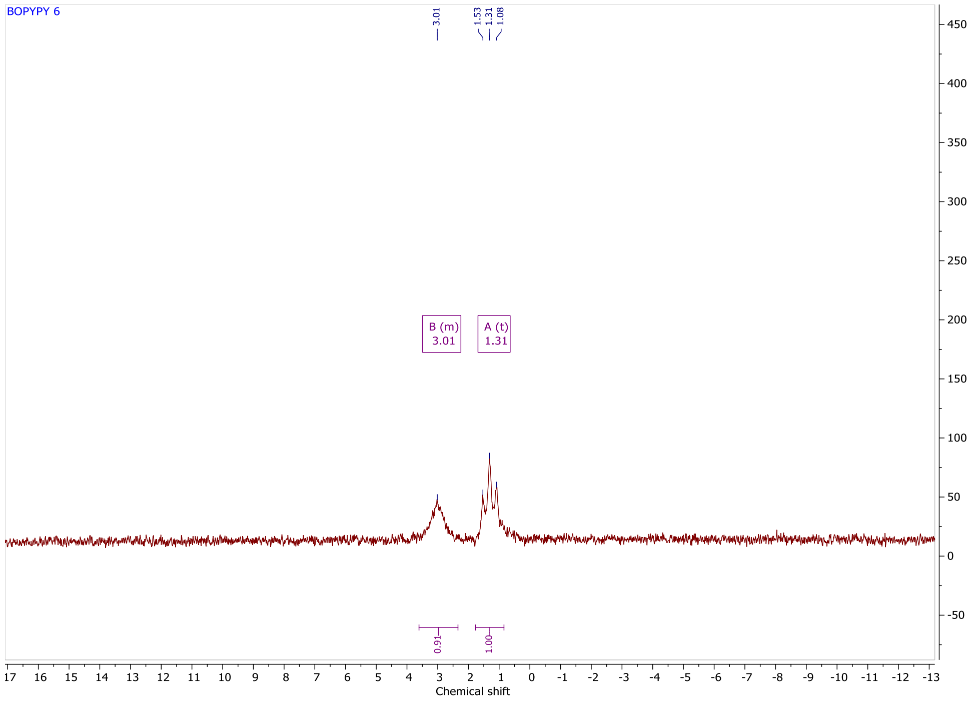

Figure S 42. ^{11}B NMR Spectrum

BOPYPY 7

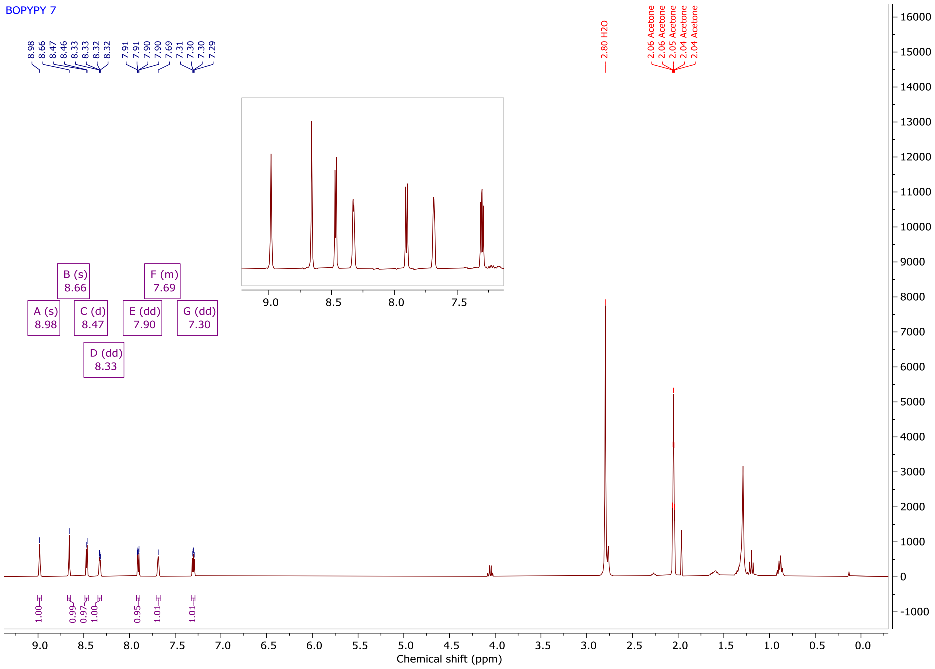

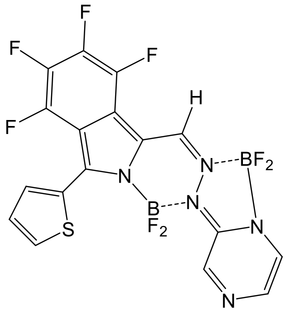

Figure S 43. ^{1}H NMR Spectrum

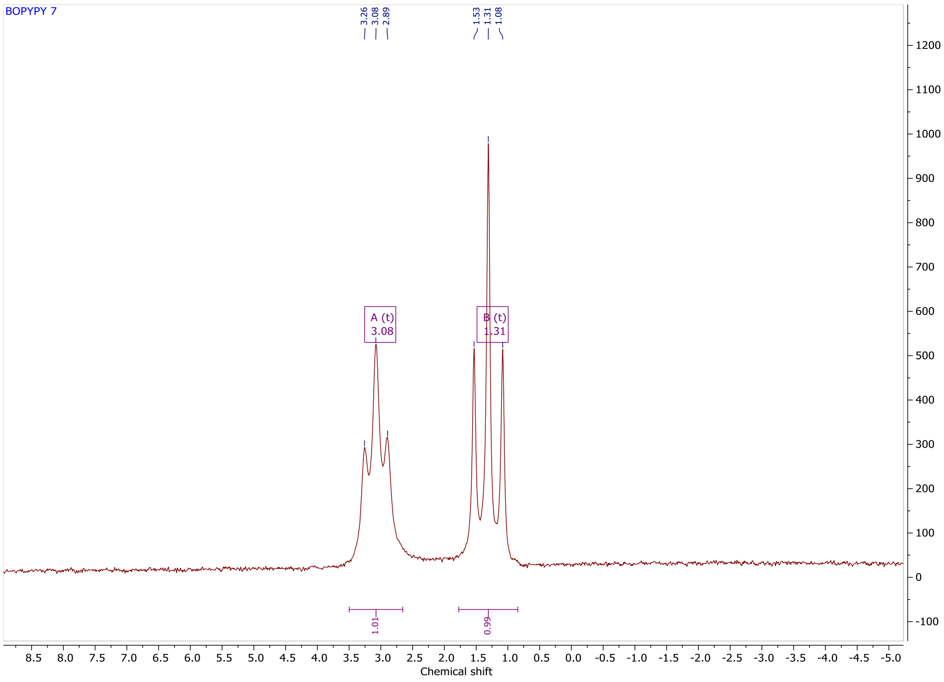

Figure S 44. ^{11}B NMR Spectrum

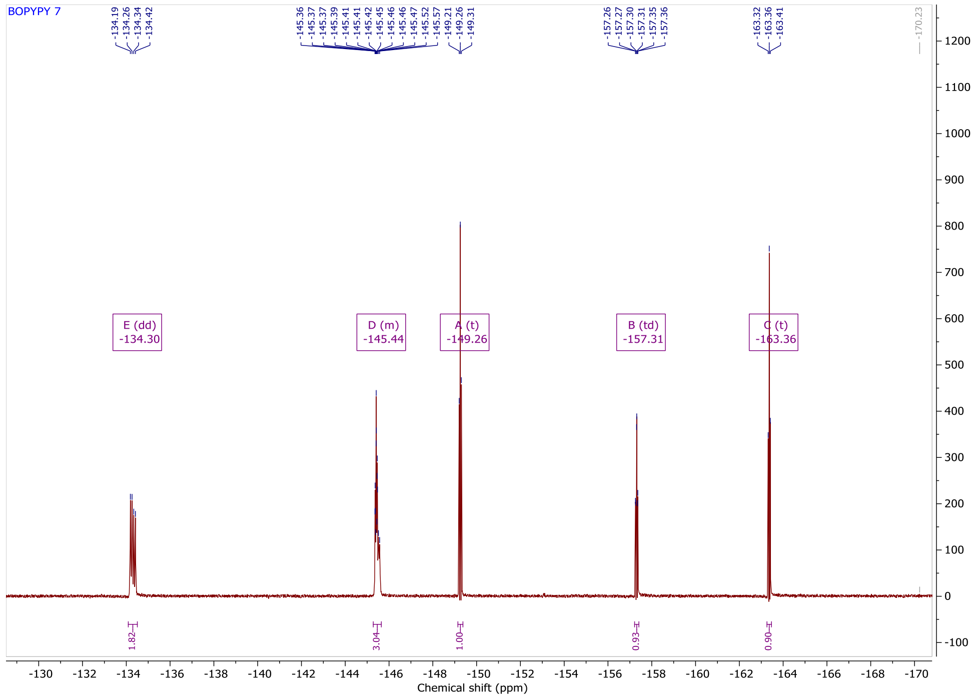

Figure S 45. ^{19}F NMR Spectrum

BOPYPY 8

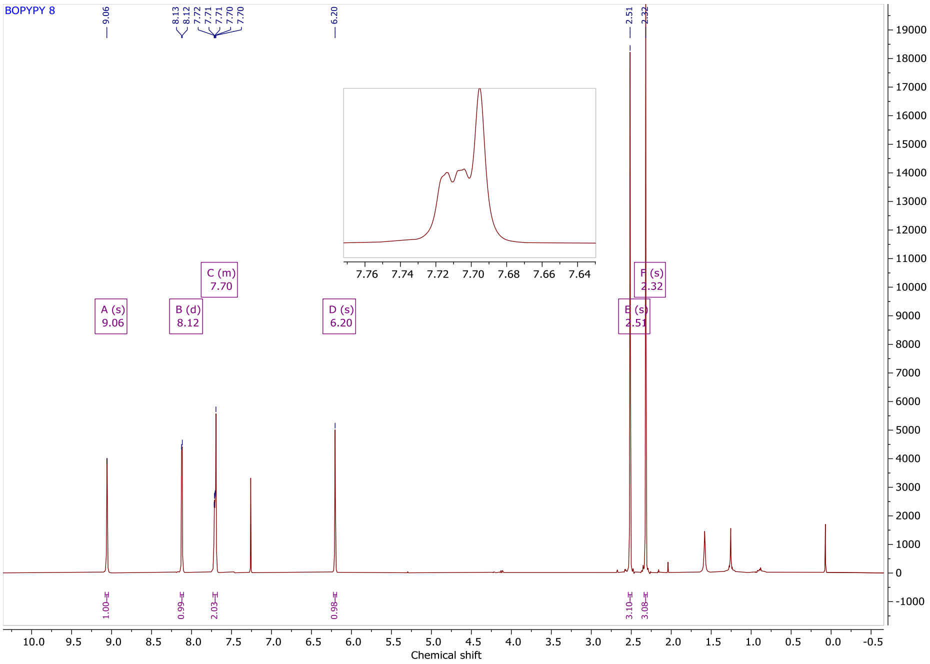

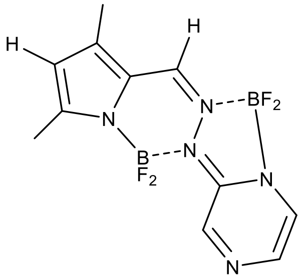

Figure S 46. ^{1}**H NMR Spectrum**

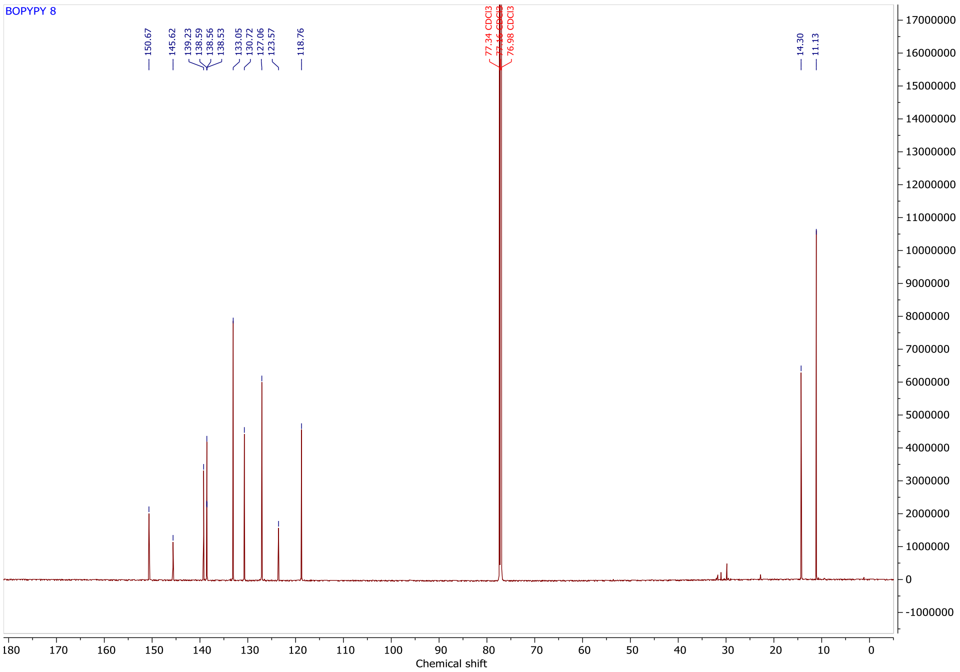

Figure S 47. ^{13}**C NMR Spectrum**

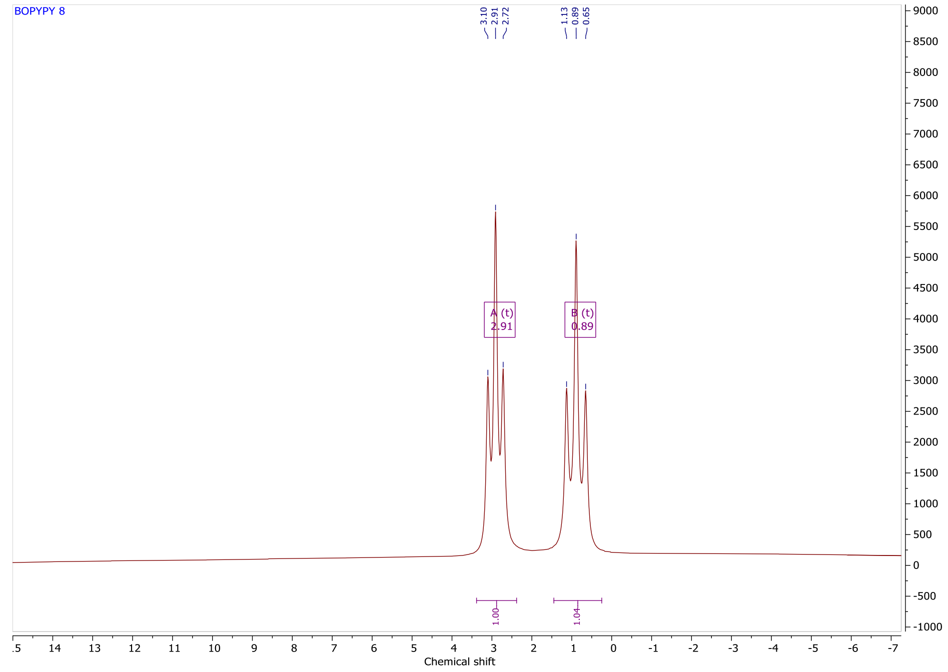

Figure S 48. ^{11}B NMR Spectrum

2. 2D NMR SPECTRA

BOPYPY 3

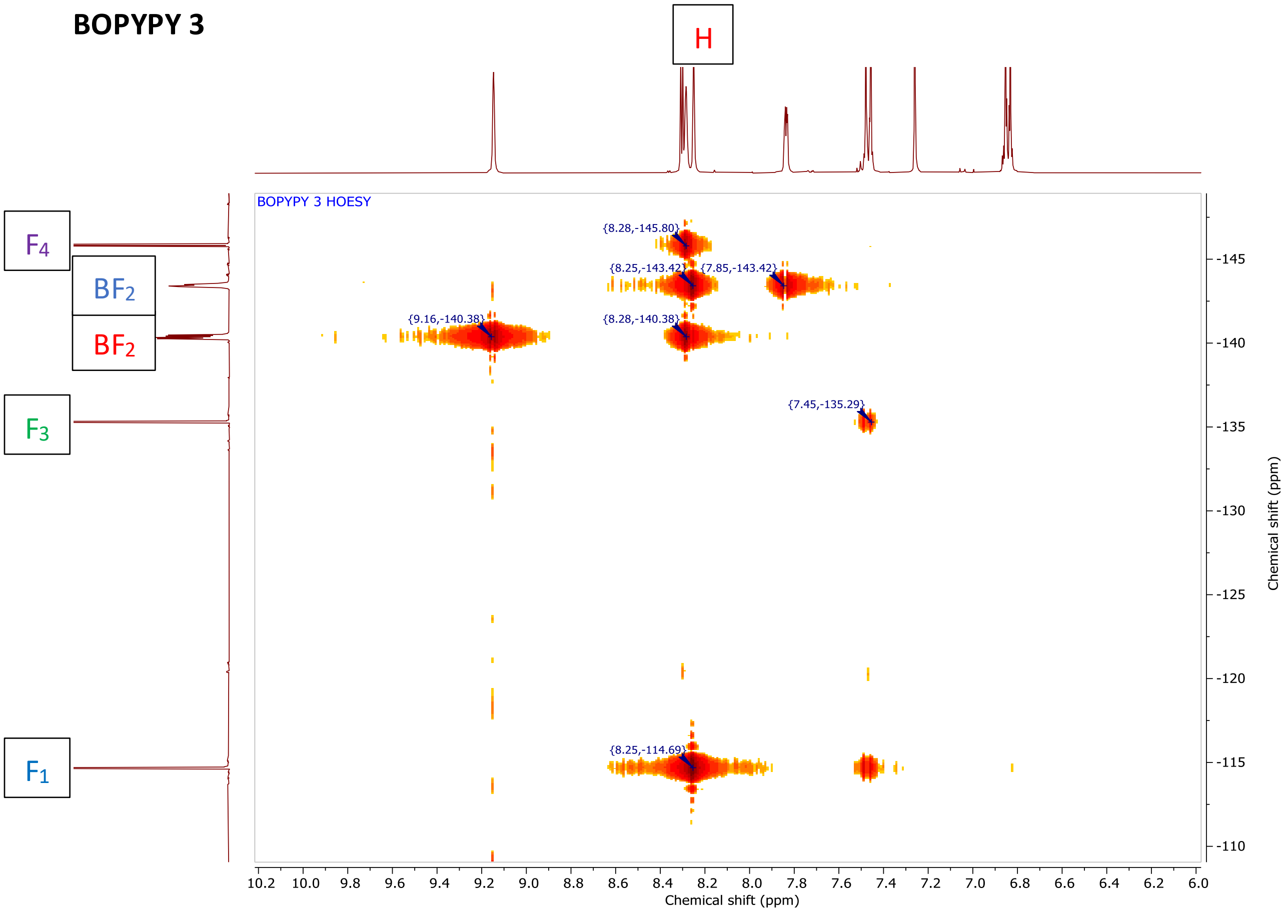

Figure S 49. 2D ^{1}H – ^{19}F HOESY Spectrum

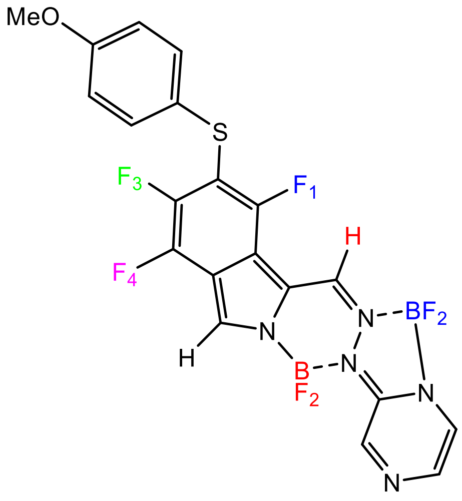

BOPYPY 5a

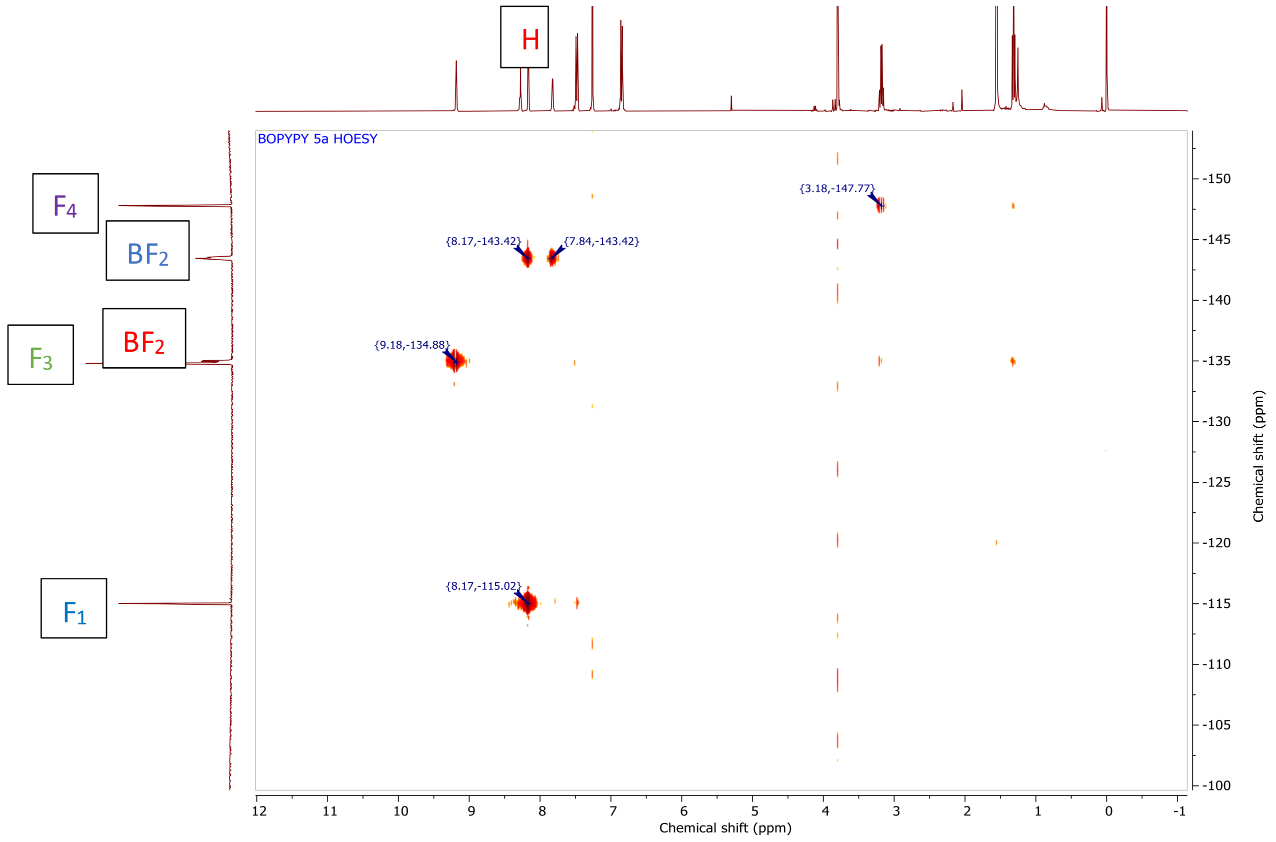

Figure S 50. 2D ^{1}H – ^{19}F HOESY Spectrum

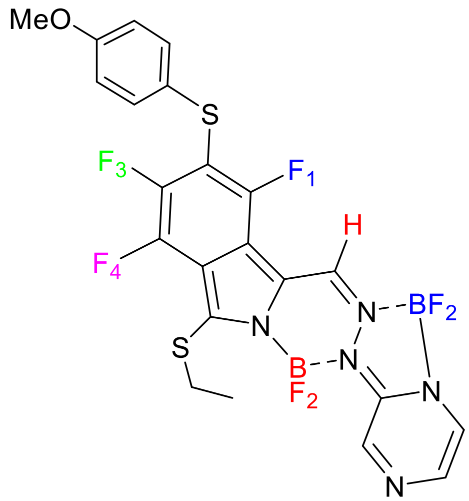

BOPYPY 5b

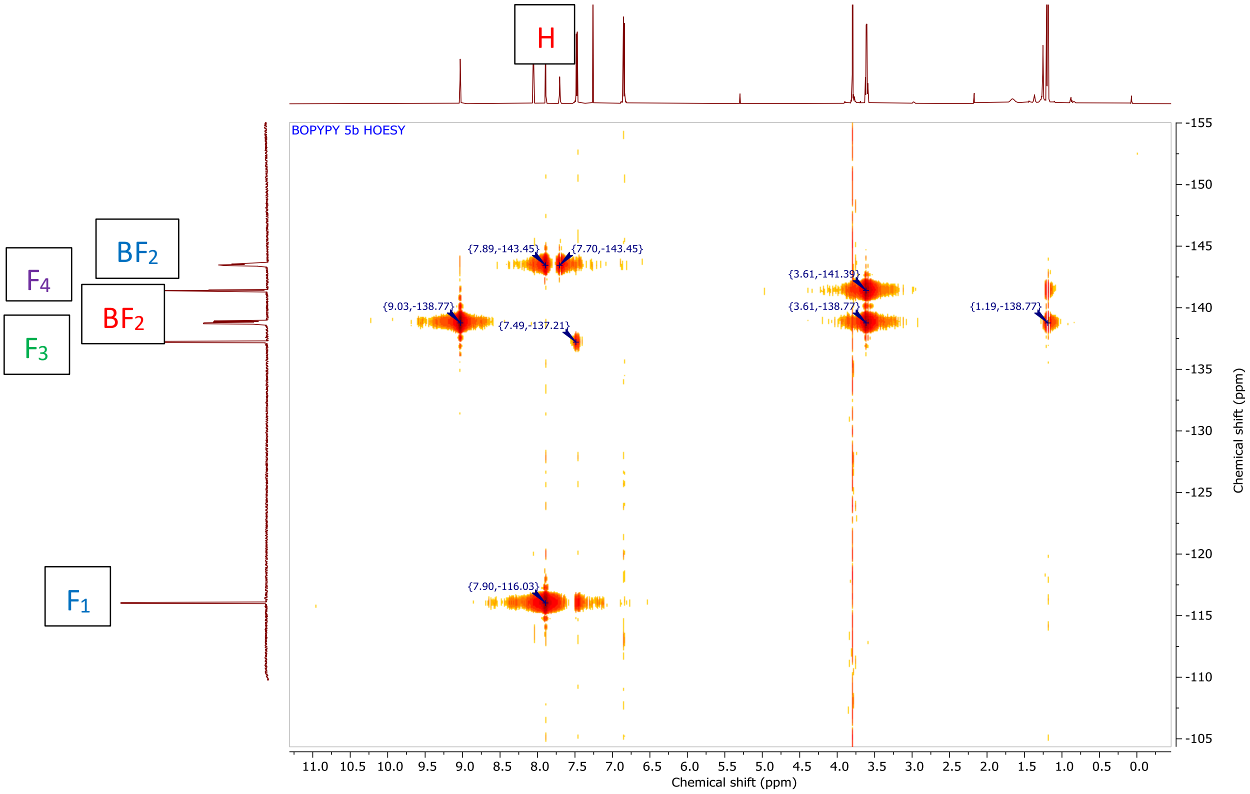

Figure S 51. 2D ^{1}H – ^{19}F HOESY Spectrum

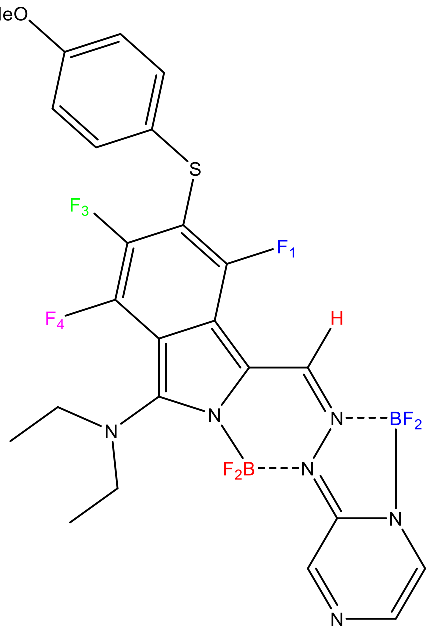

BOPYPY 6

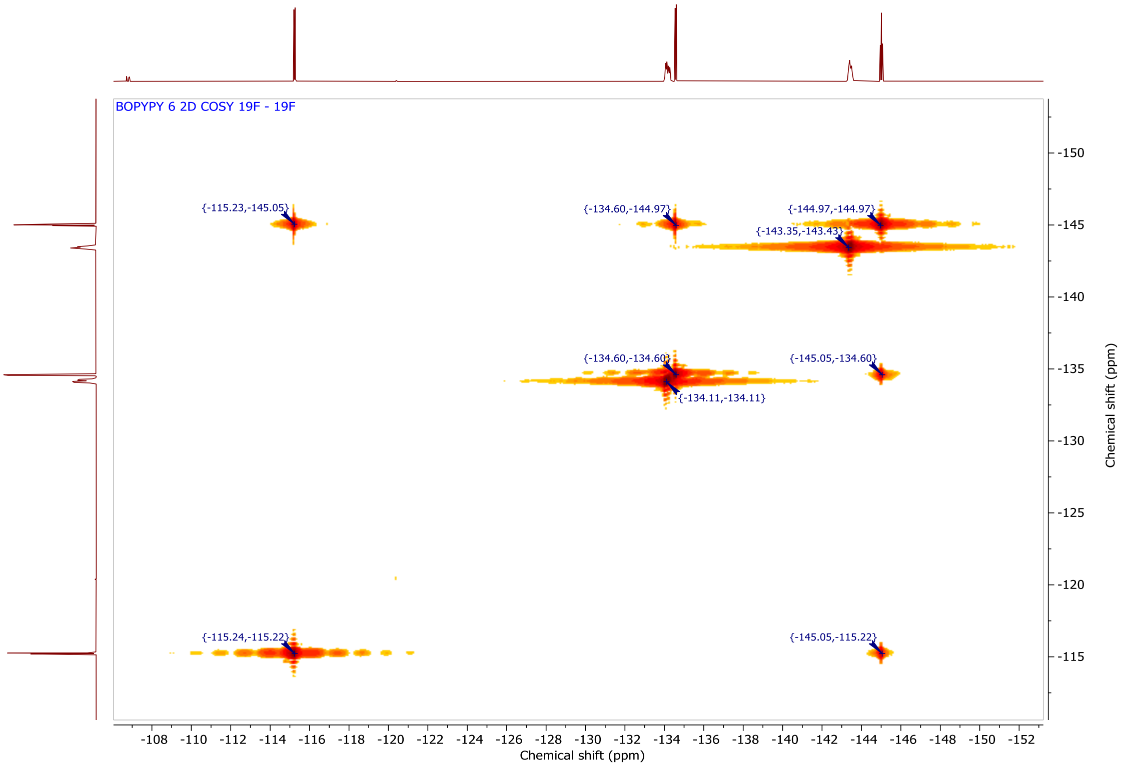

Figure S 52. 2D COSY ^{19}F – ^{19}F Spectrum

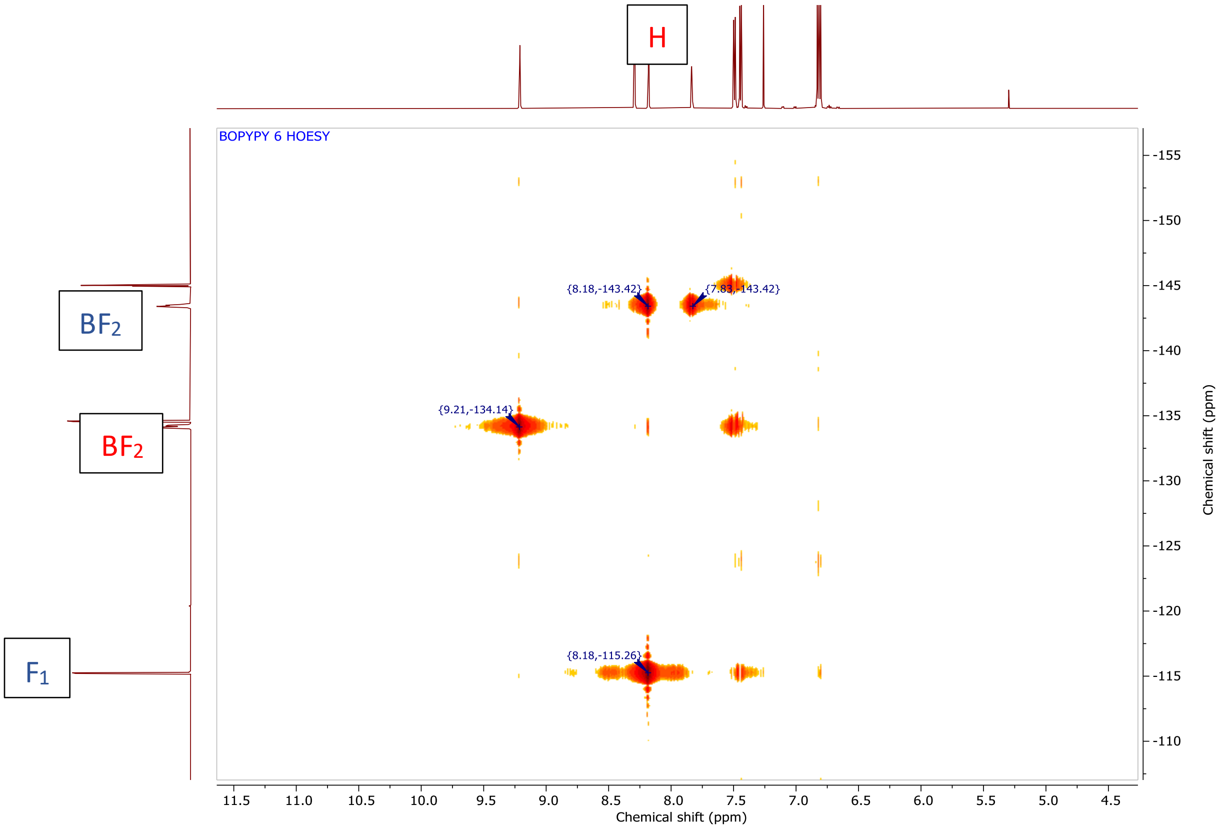

Figure S 53. 2D ^{1}H – ^{19}F HOESY Spectrum

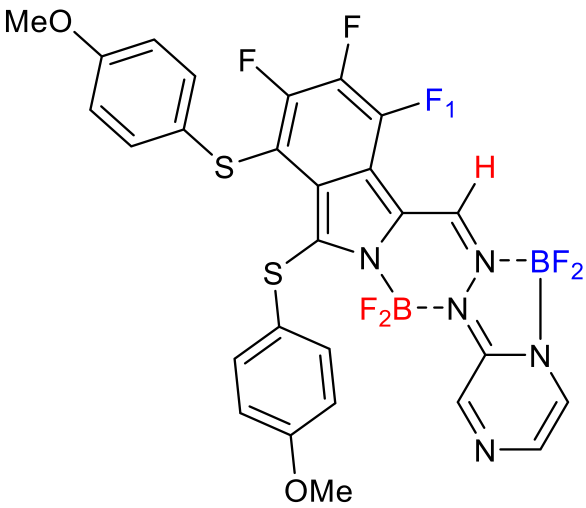

3. X-ray Crystallography structures

BOPYPY 8

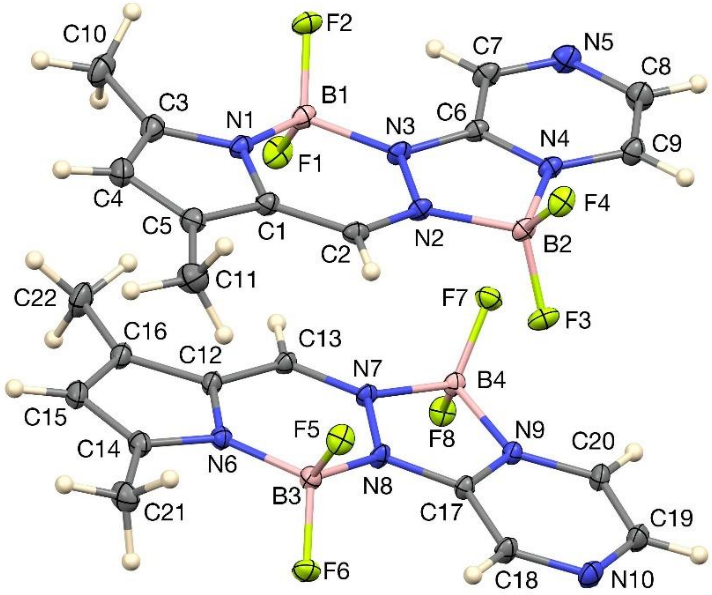

Figure S 54. X-ray crystal structures of BOPYPY 8 with 50% ellipsoids

BOPYPY 9

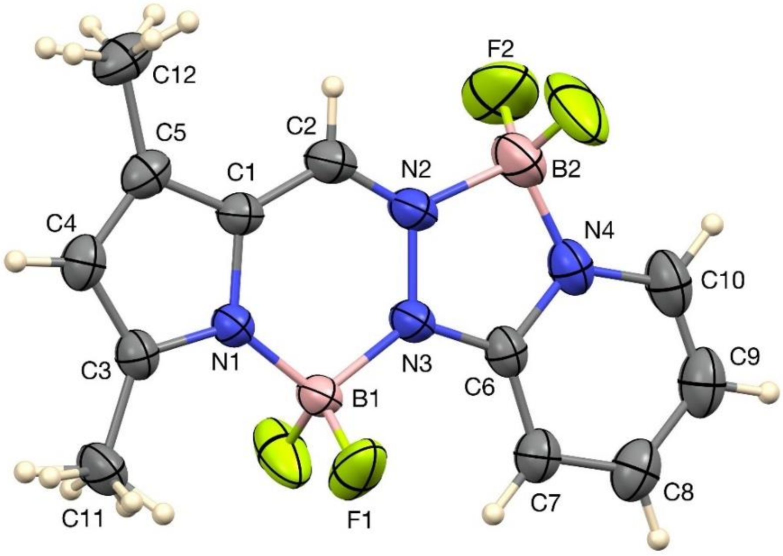

Figure S 55. X-ray crystal structures of BOPYPY 9 with 50% ellipsoids

4. Spectroscopic data

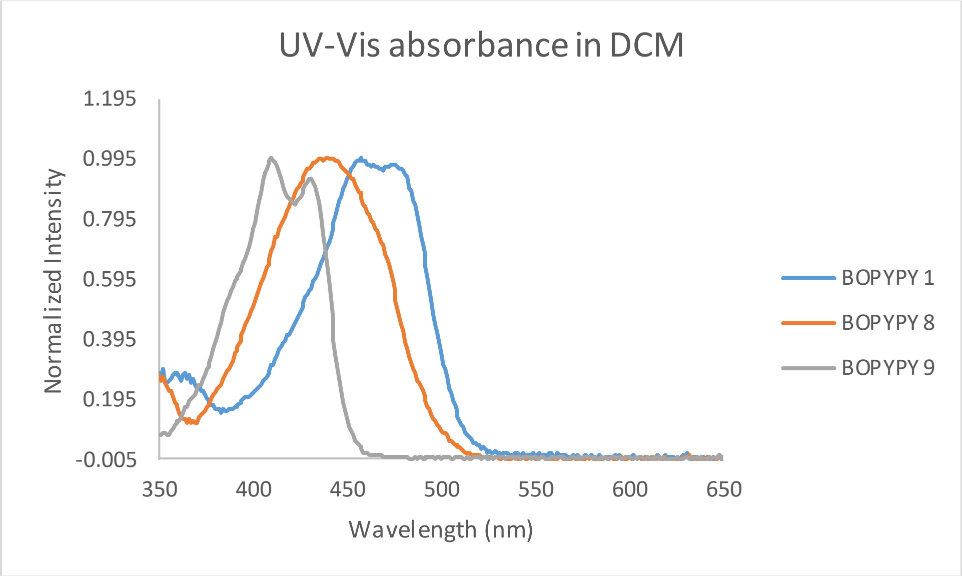

Figure S 56. Absorbance spectra for BOPYPYs 1, 3 and 8 compared to BOPYPY 9

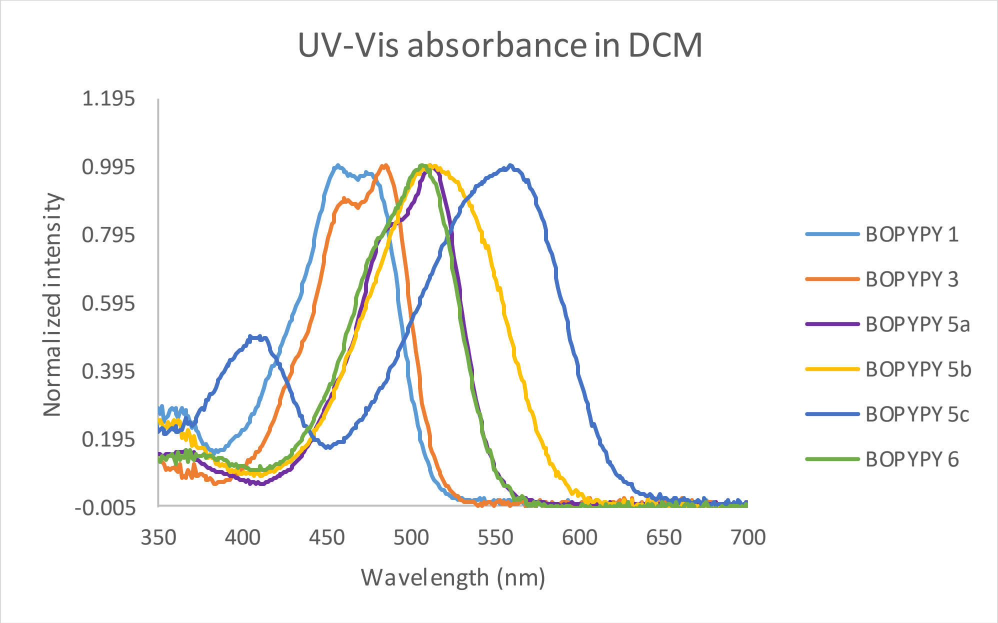

Figure S 57. Absorbance spectra for BOPYPYs 1, 3 and disubstituted BOPYPYs

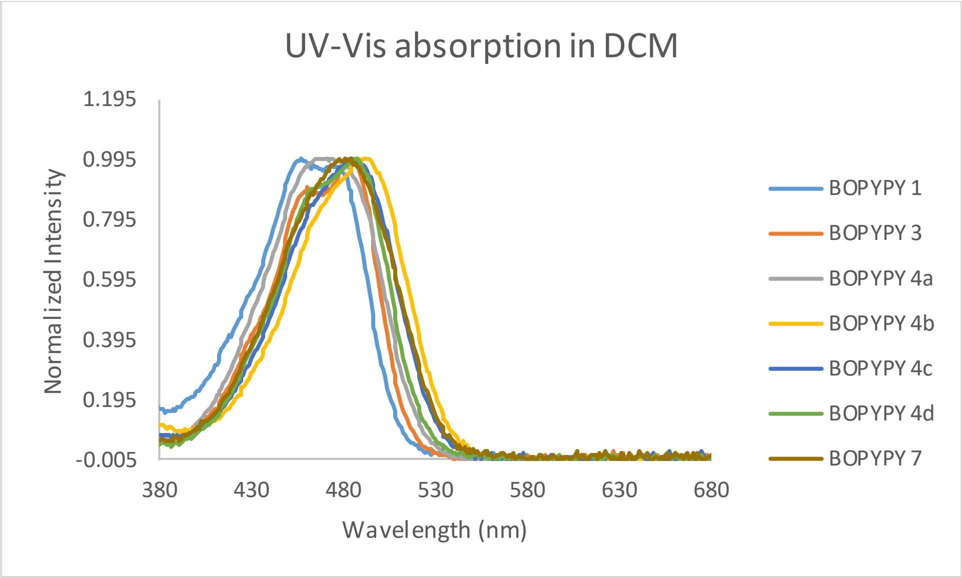

Figure S 58. Absorption spectra for BOPYPYs 1, 3 and monosubstituted BOPYPYs

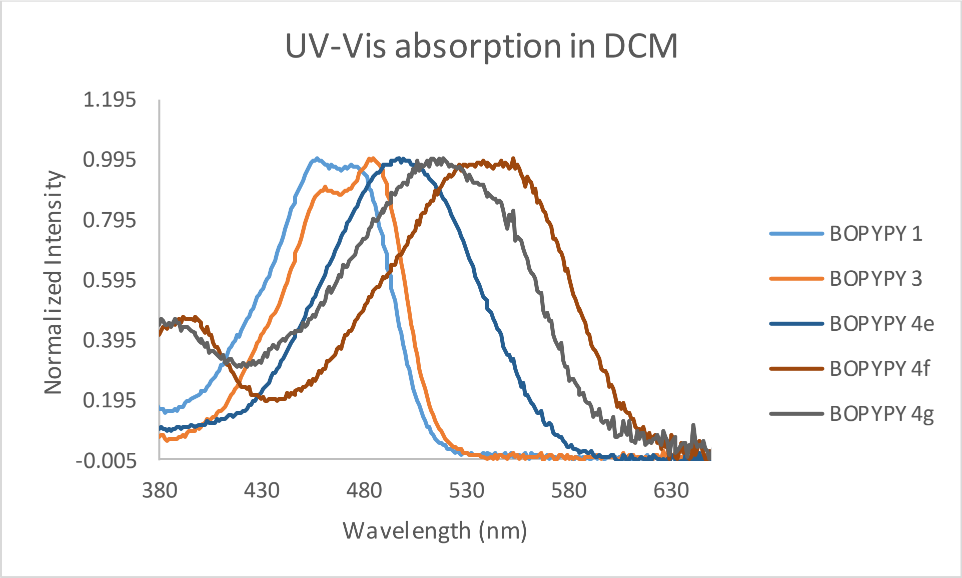

Figure S 59. Absorption spectra for BOPYPYs 1, 3 and monosubstituted BOPYPYs

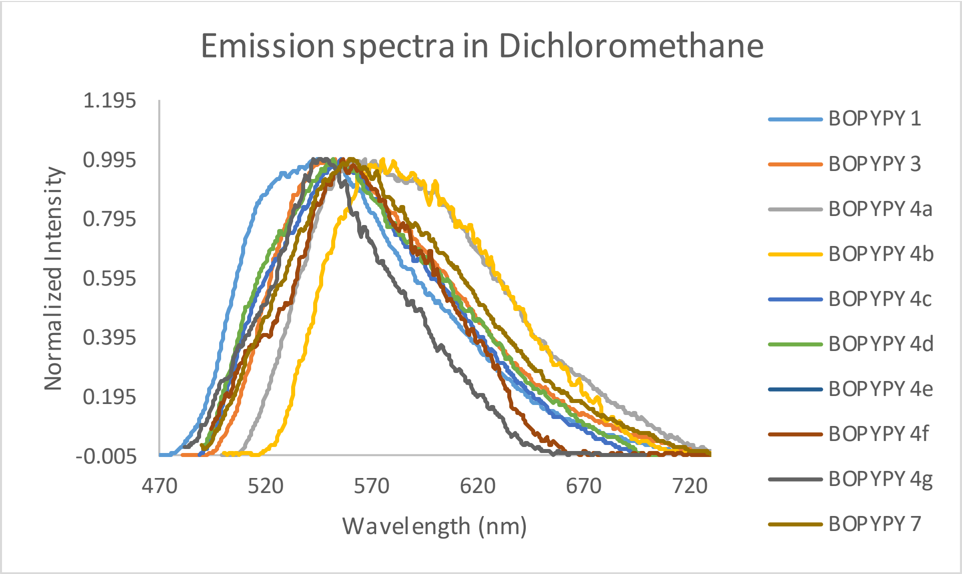

Figure S 60. Emission spectra for BOPYPYs 1 and 3 compared to monosubstituted BOPYPYs

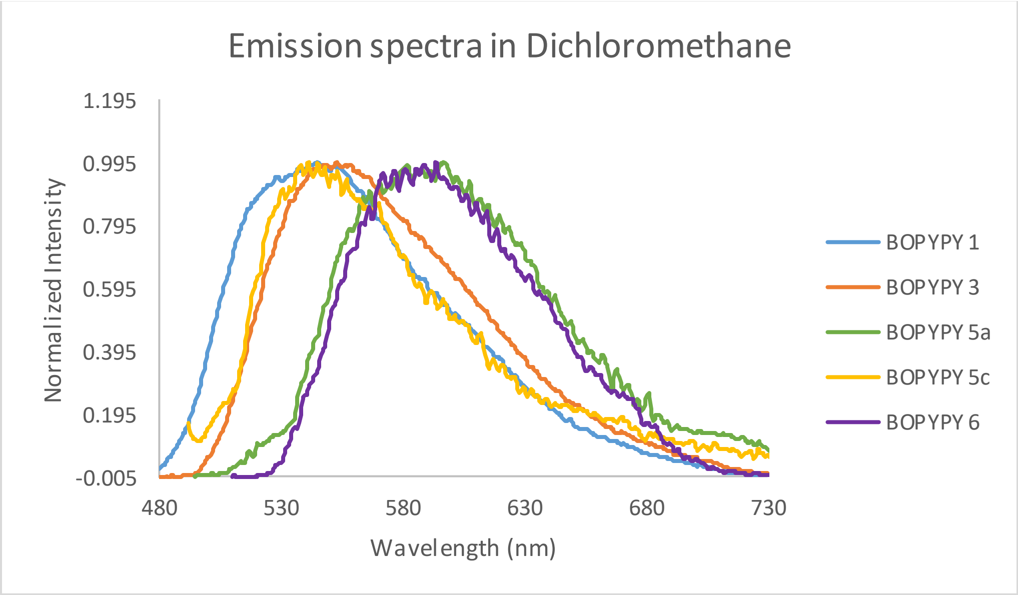

Figure S 61. Emission spectra for BOPYPYs 1, 3 and disubstituted BOPYPYs

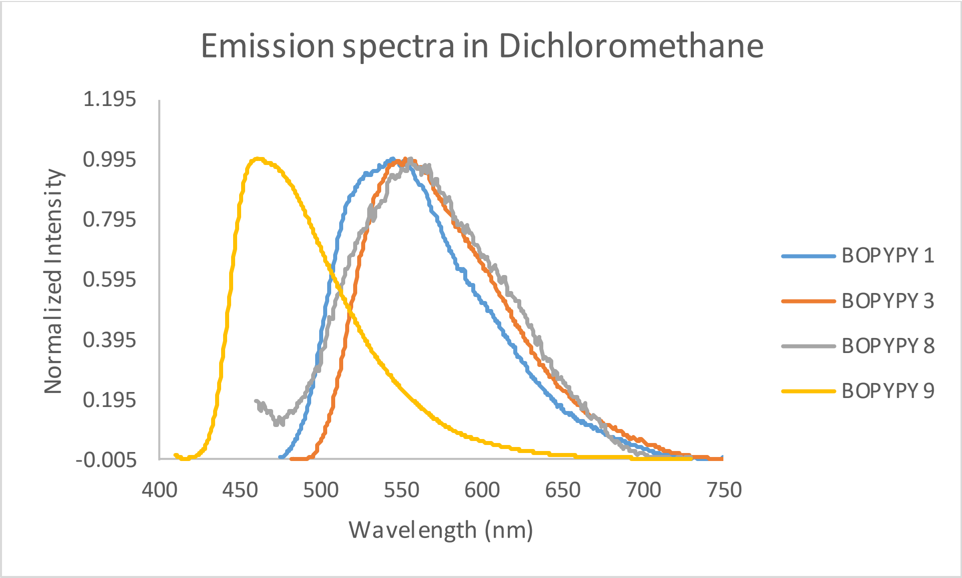

Figure S 62. Emission spectra for BOPYPYs 1, 3 and 8 compared to BOPYPY 9

5. Computational studies data

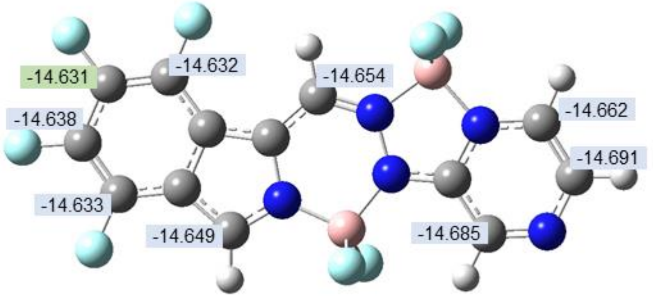

Figure S 63. ωB97XD/6-311++G(d,p) Molecular Electrostatic Potentials (MESP, a.u.) at the carbon nuclei for BOPYPY 1. The MESPs of the sites most susceptible to nucleophilic substitution are indicated in green.

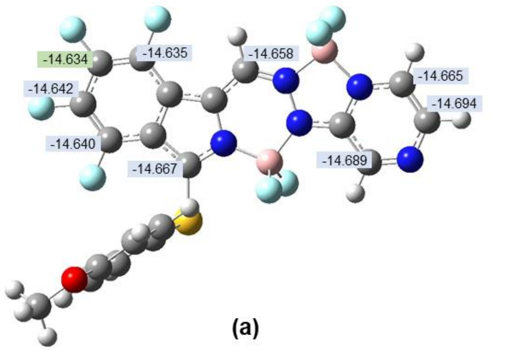

(a)

BOPYPY **4b**

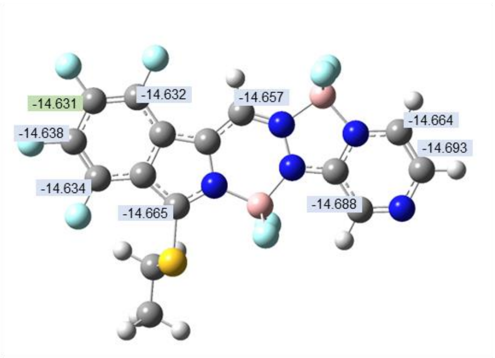

(b)

BOPYPY **4d**

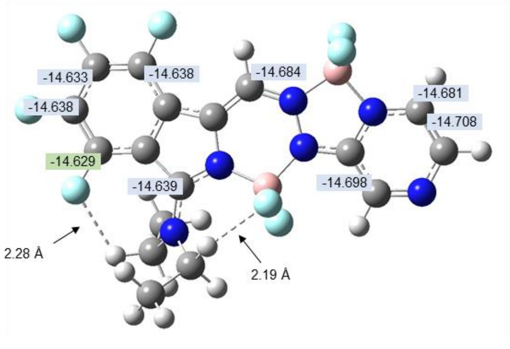

(c)

BOPYPY **4e**

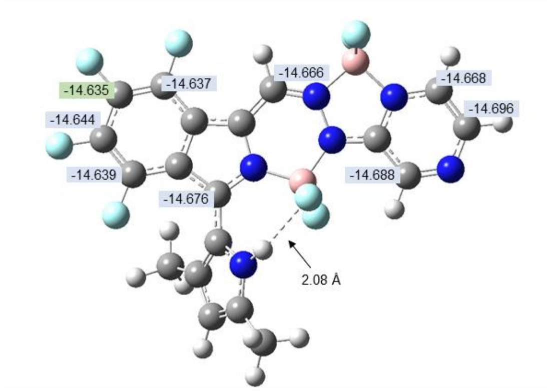

(d)

BOPYPY **4g**

Figure S 64. ωB97XD/6-311++G(d,p) Molecular Electrostatic Potentials (MESP, in a.u.) at the carbon nuclei for a) BOPYPY 4b, b) BOPYPY 4d, c) BOPYPY 4e, d) BOPYPY 4g. The MESPs of the sites most susceptible to nucleophilic substitution are indicated in green.

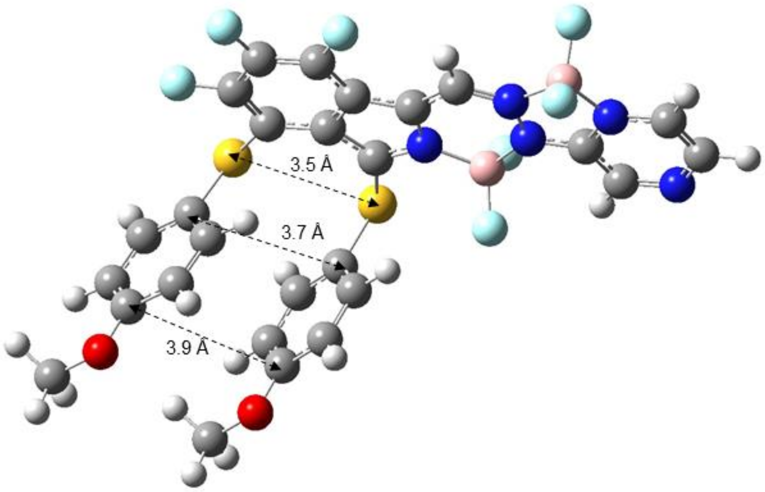

Figure S 65. ωB97XD/6-311++G(d,p) optimized geometry of BOPYPY 6.

Table S 1. TD-DFT M06-2X/6-31+G(d,p) calculated spectroscopic, electronic, and structural properties of the ground and excited states of the series of BOPYPYs studied. All parameters are calculated in DCM. The leading transition is $S_0 \rightarrow S_1$ all cases.

Compound	λ_{abs} (nm)	Oscillator Strength	λ_{em} (nm)	Stokes shift (nm)	HOMO (eV)	LUMO (eV)	HOMO-LUMO gap (eV)	$R_{NN}(S_1)$-$R_{NN}(S_0)$ (Å)
**1**	397	0.936	470	73	-7.31	-2.27	5.04	0.048
**3**	410	1.085	484	74	-7.19	-2.29	4.89	0.043
**4a**	412	1.060	489	77	-7.06	-2.16	4.90	0.049
**4b**	423	0.956	510	87	-7.09	-2.30	4.79	0.037
**4c**	426	0.967	507	81	-7.07	-2.30	4.77	0.038
**4d**	416	0.916	498	82	-7.17	-2.29	4.88	0.042
**4e**	438	0.870	530	92	-6.66	-1.95	4.71	0.049
**4f**	472	0.820	568	96	-6.58	-2.21	4.37	0.032
**4g**	463	0.805	559	96	-6.67	-2.23	4.44	0.033
**5a**	428	0.966	509	81	-7.10	-2.35	4.76	0.043
**5b**	451	0.914	545	94	-6.59	-1.99	4.61	0.047
**5c**	475	0.826	573	98	-6.61	-2.26	4.35	0.032
**6**	427	0.985	510	83	-7.05	-2.31	4.74	0.033
**7**	424	0.909	524	100	-7.10	-2.30	4.79	0.039
**8**	376	0.770	463	87	-7.27	-1.93	5.34	0.058
**9**	361	0.843	423	62	-7.10	-1.57	5.53	0.050

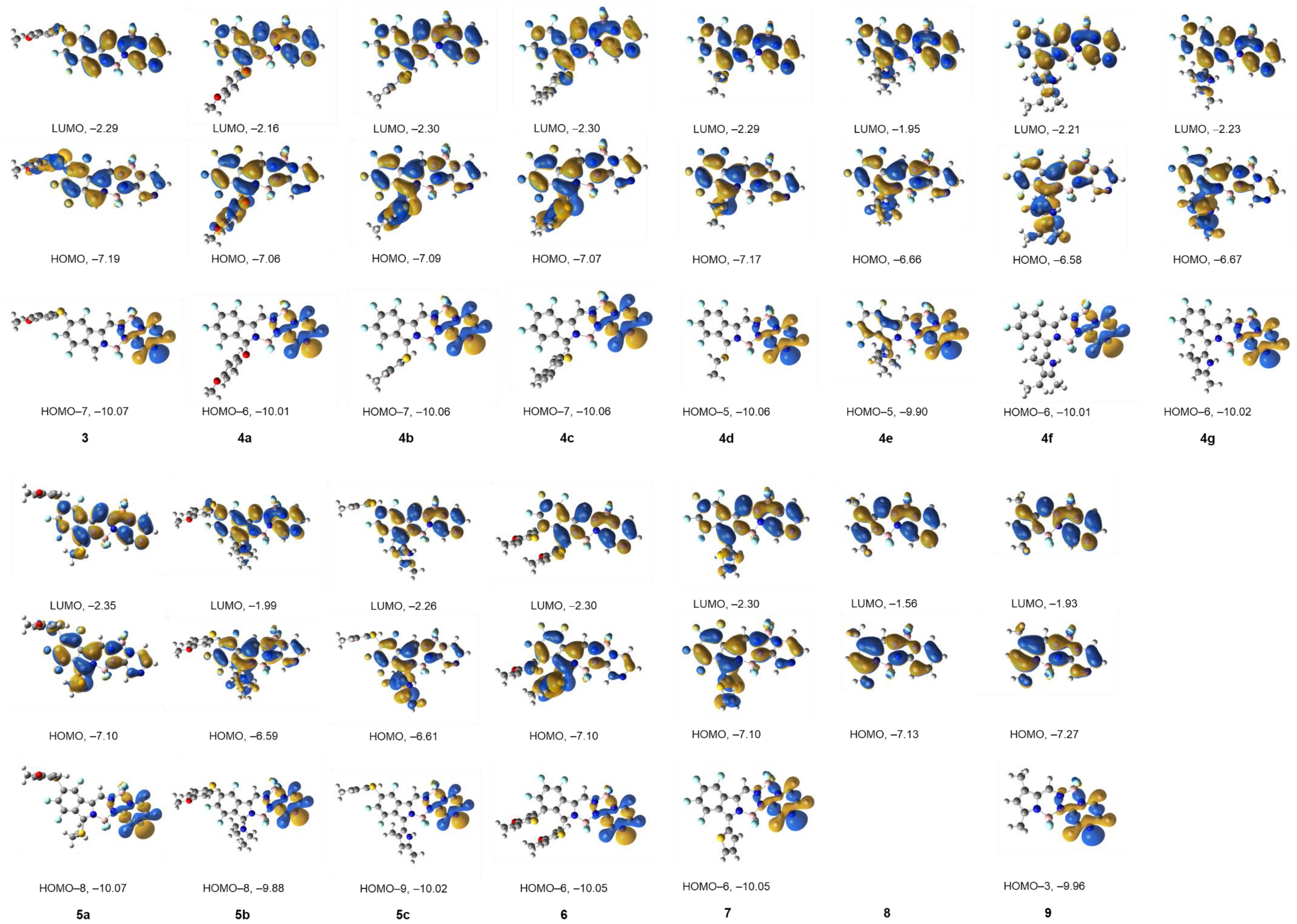

Figure S 66. Frontier orbitals for the series of the BOPPY and BOPYPYs

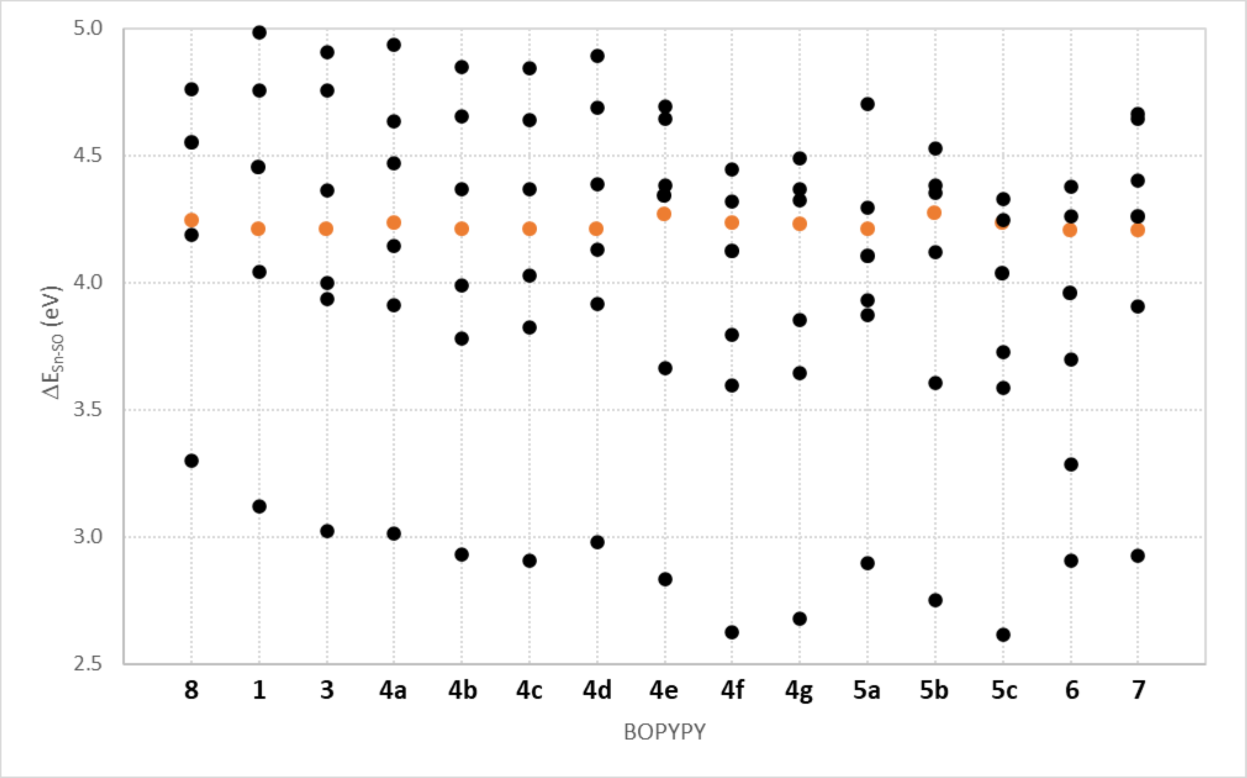

Figure S 67. Energies of the lowest-lying excited states (compared to the energy of S_0) for the series of BOPYPYs studied. The dark state is shown in orange.

6. HRMS Data

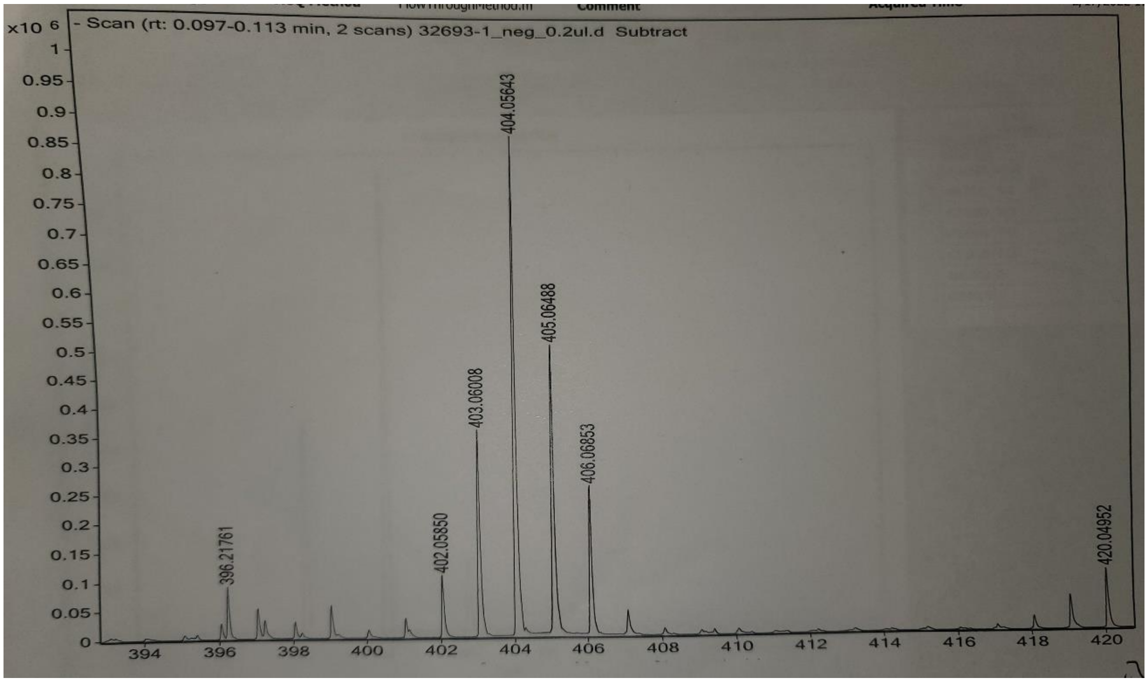

Figure S 68. ESI TOF spectrum for BOPYPY 1

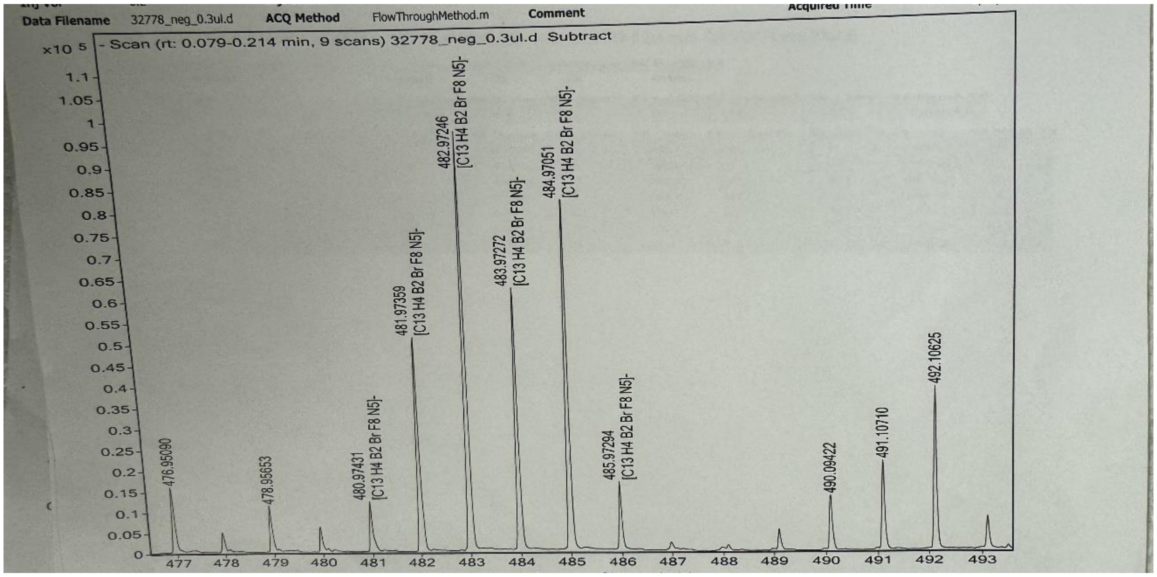

Figure S 69. ESI TOF spectrum for BOPYPY 2

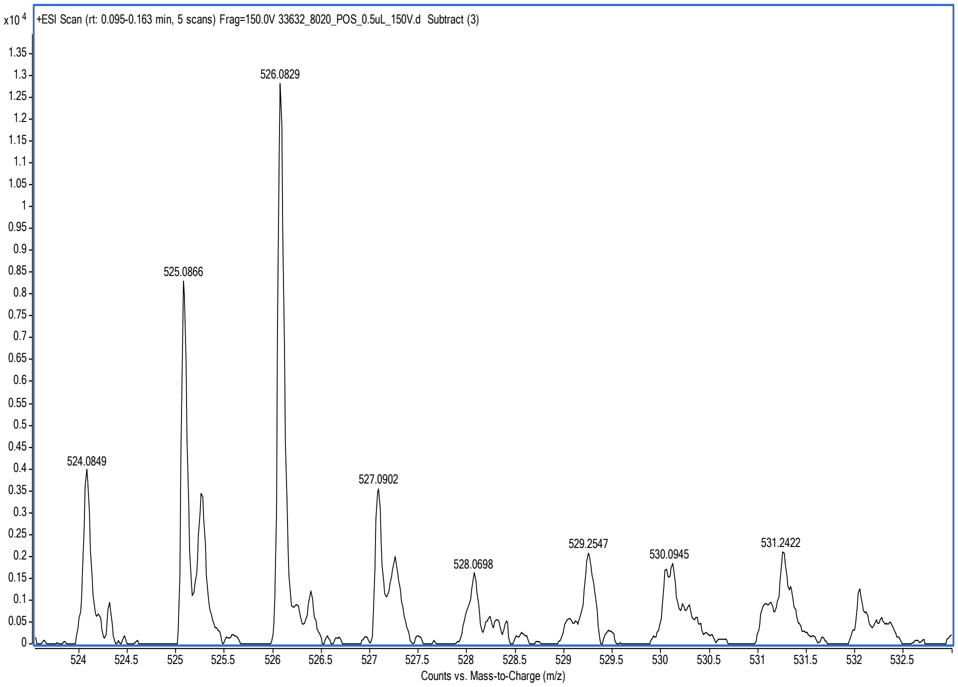

Figure S 70. ESI TOF spectrum for BOPYPY 3

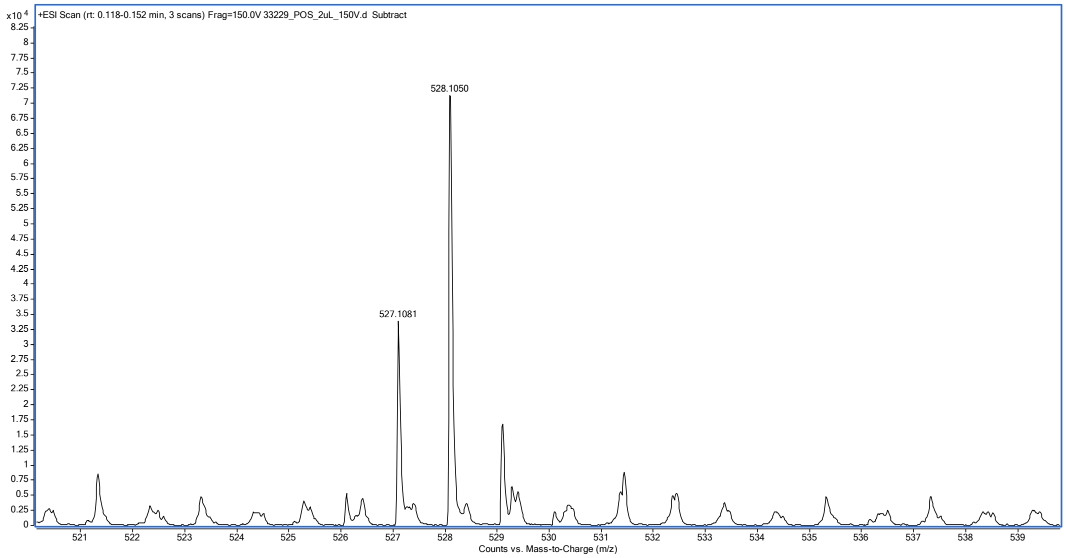

Figure S 71. ESI TOF spectrum for BOPYPY 4a

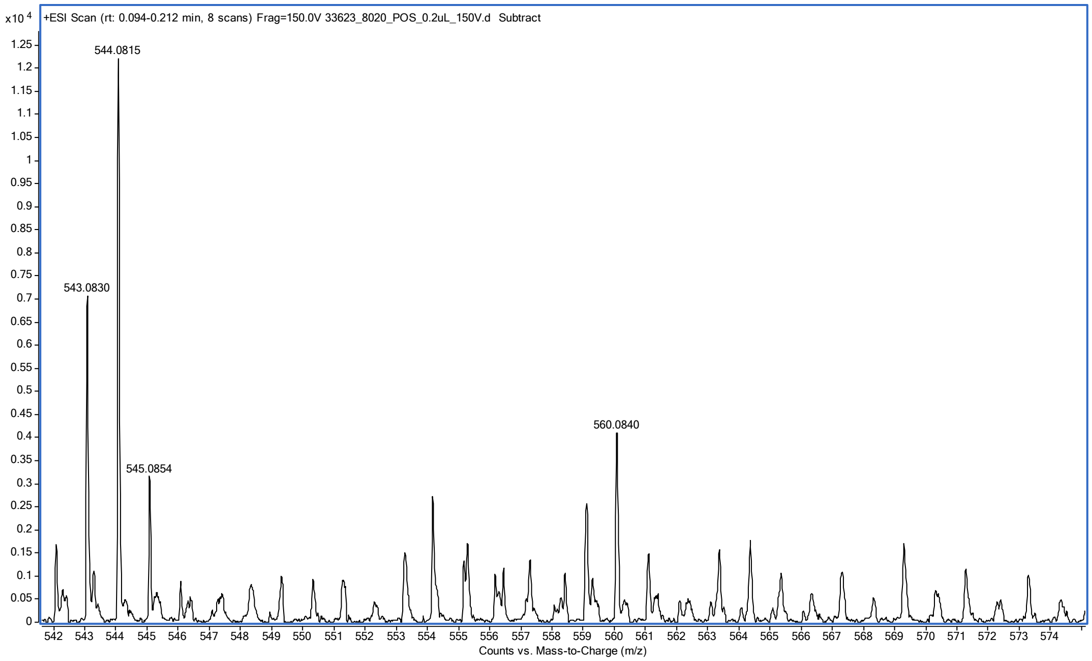

Figure S 72. ESI TOF spectrum for BOPYPY 4b

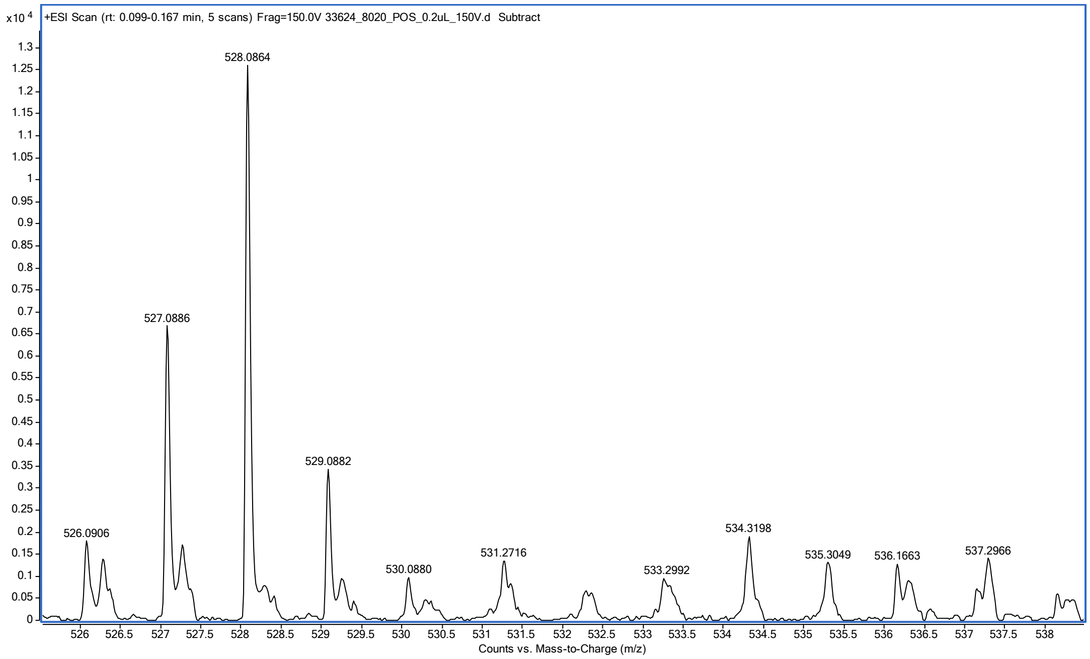

Figure S 73. ESI TOF spectrum for BOPYPY 4c

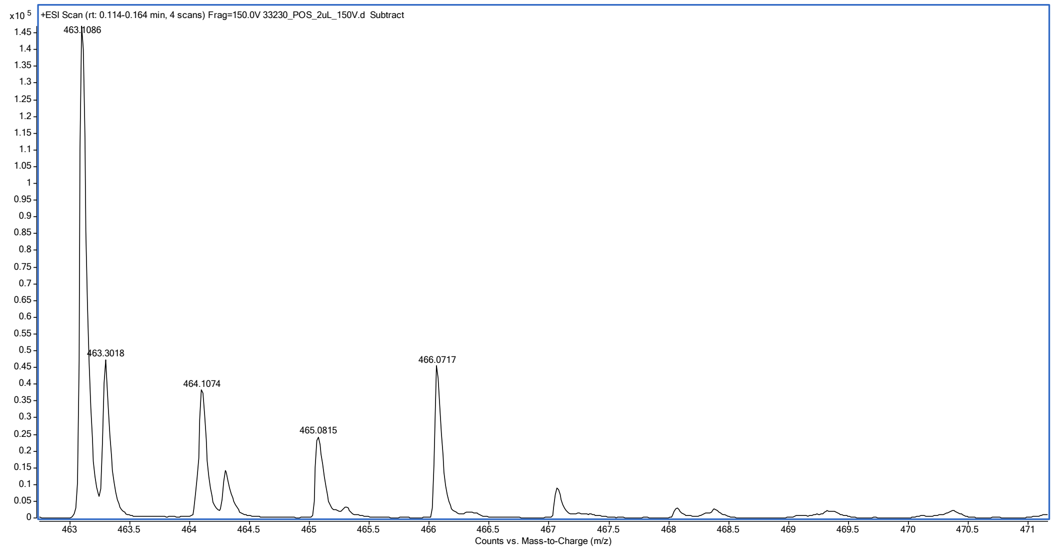

Figure S 74. ESI TOF spectrum for BOPYPY 4d

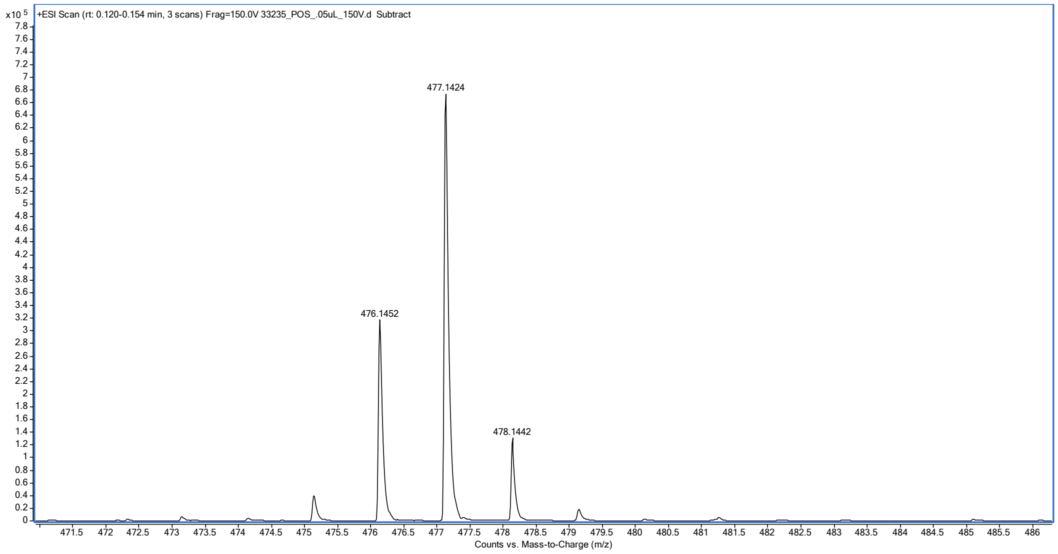

Figure S 75. ESI TOF spectrum for BOPYPY 4e

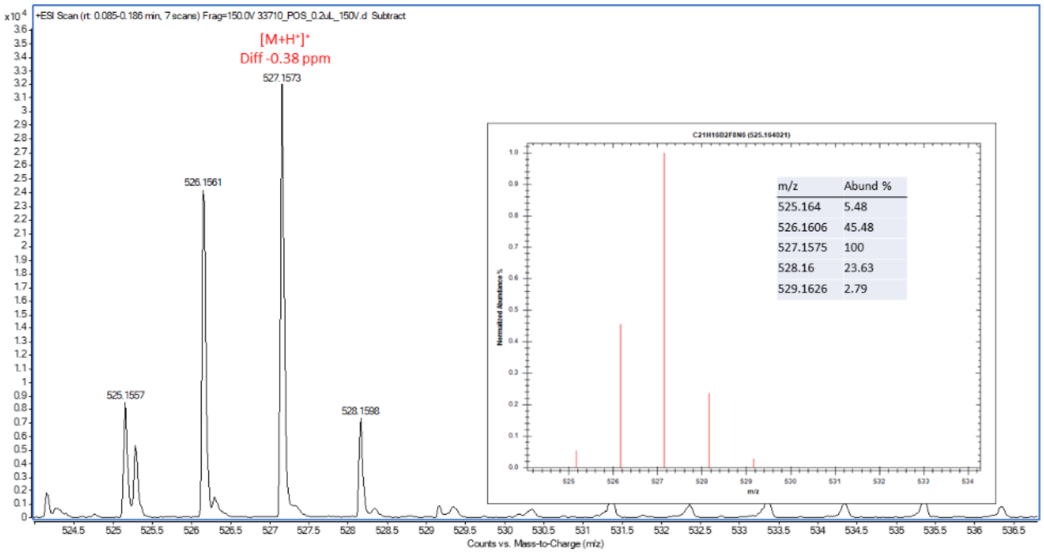

Figure S 76. ESI TOF spectrum for BOPYPY 4f

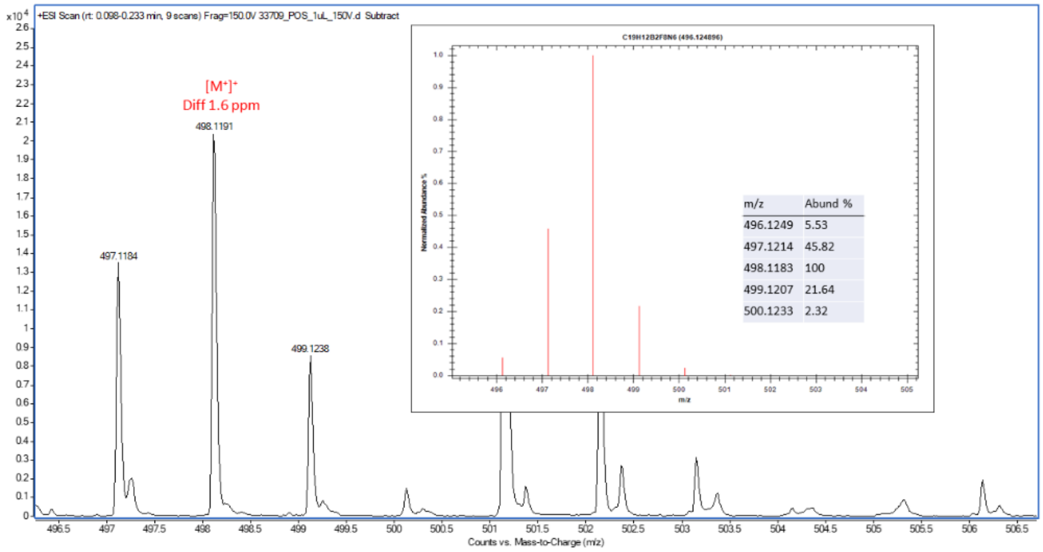

Figure S 77. ESI TOF spectrum for BOPYPY 4g

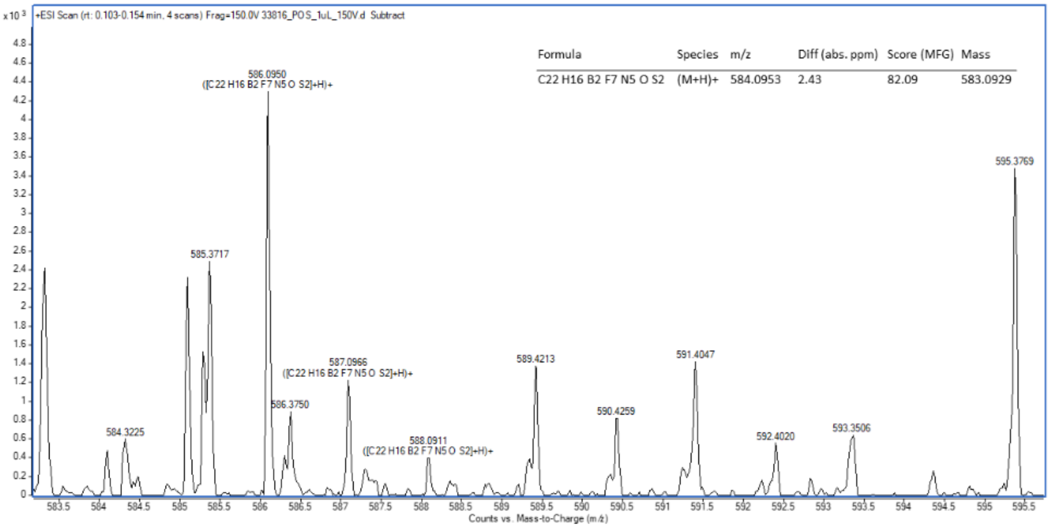

Figure S 78. ESI TOF spectrum for BOPYPY 5a

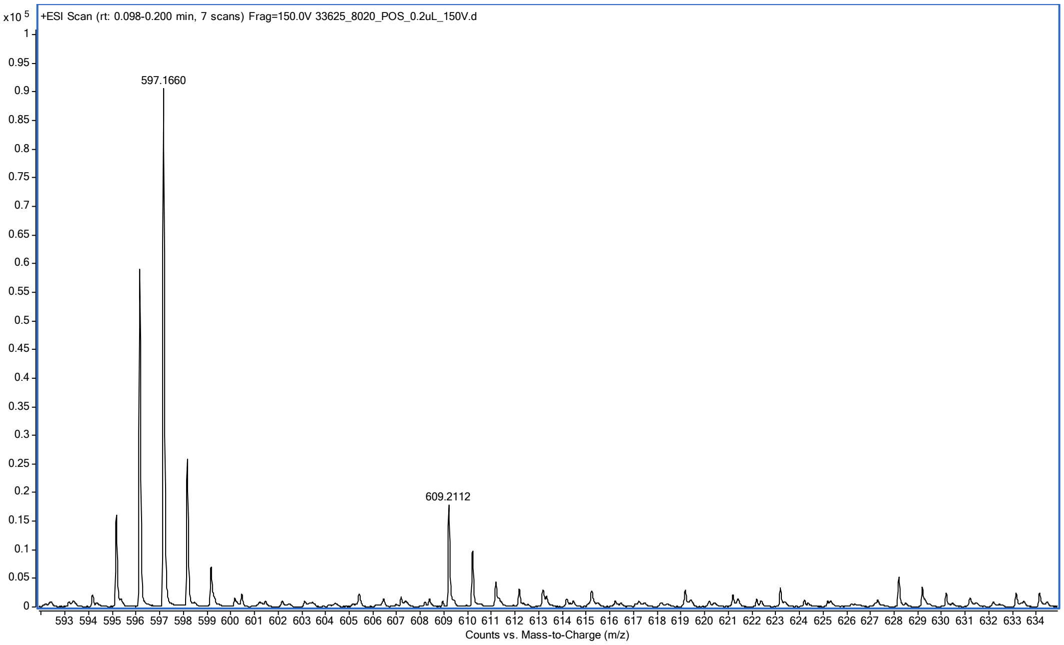

Figure S 79. ESI TOF spectrum for BOPYPY 5b

DCTB

Spectrum in in the 160-2000 m/z range

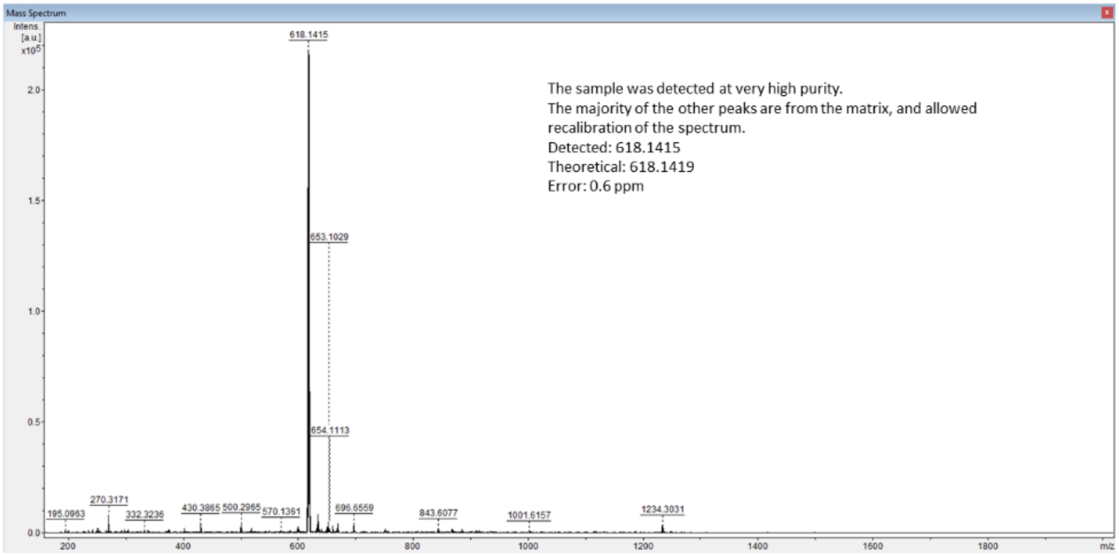

Figure S 80. RapifleX MALDI spectrum for BOPYPY 5c

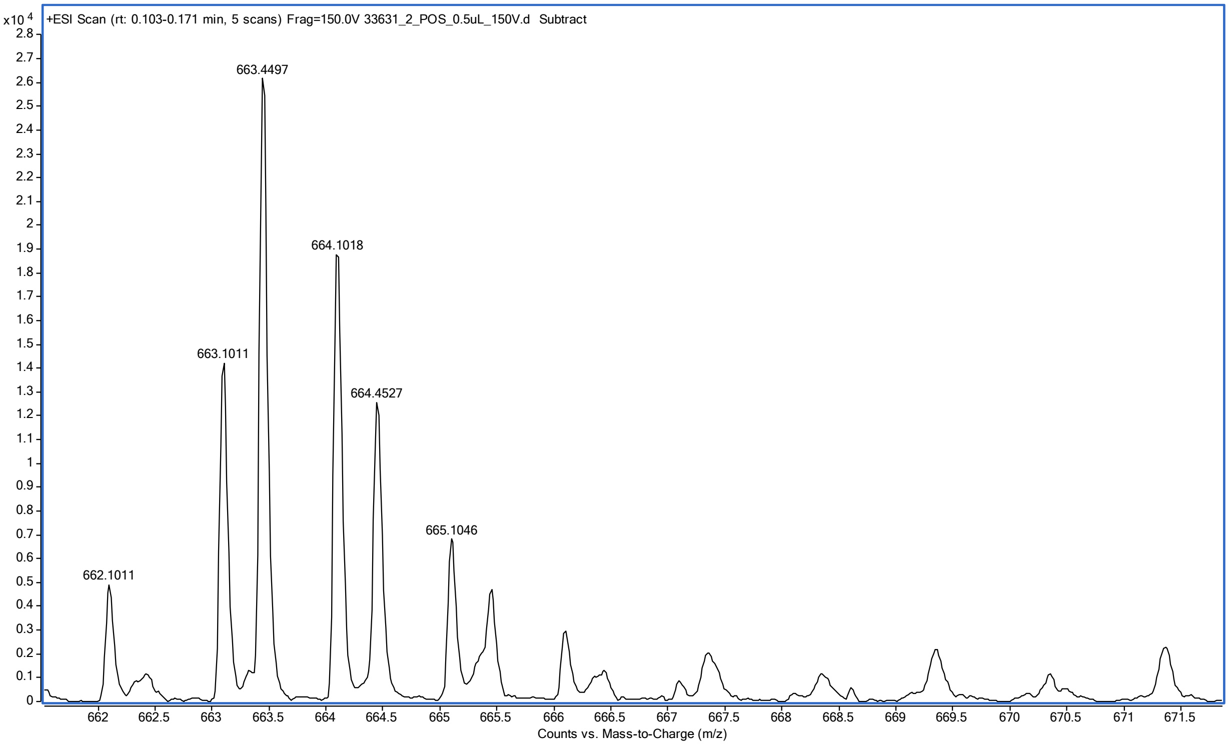

Figure S 81. ESI TOF spectrum for BOPYPY 6

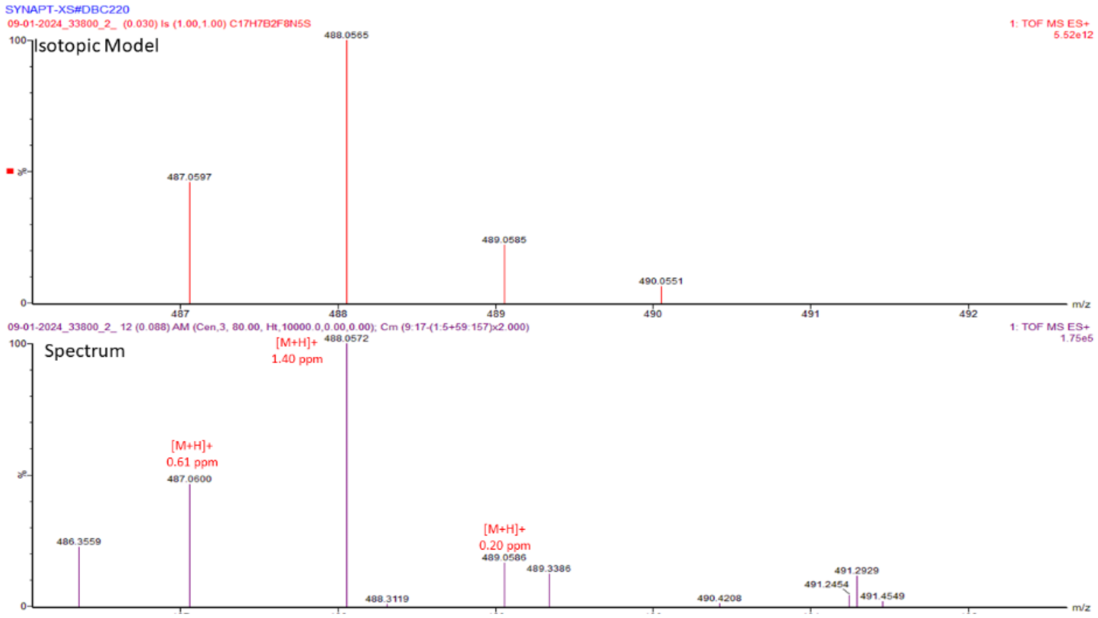

Figure S 82. Synapt XS spectrum for BOPYPY 7

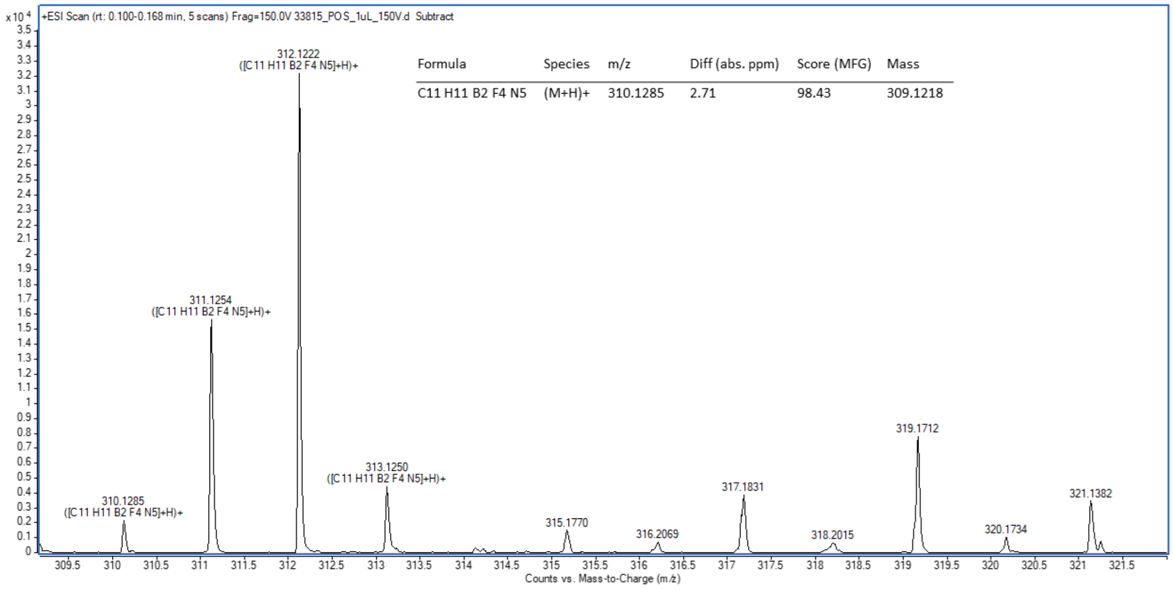

Figure S 83. ESI TOF spectrum for BOPYPY 8
